# Detection of an anti-angina therapeutic module in the effective population treated by a multi-target drug Danhong injection: a randomized trial

**DOI:** 10.1038/s41392-021-00741-x

**Published:** 2021-09-01

**Authors:** Jun Liu, Dan-Dan Li, Wei Dong, Yu-Qi Liu, Yang Wu, Da-Xuan Tang, Fu-Chun Zhang, Meng Qiu, Qi Hua, Jing-Yu He, Jun Li, Bai Du, Ting-Hai Du, Lin-Lin Niu, Xue-Jun Jiang, Bo Cui, Jiang-Bin Chen, Yang-Gan Wang, Hai-Rong Wang, Qin Yu, Jing He, Yi-Lin Mao, Xiao-Fang Bin, Yue Deng, Yu-Dan Tian, Qing-Hua Han, Da-Jin Liu, Li-Qin Duan, Ming-Jun Zhao, Cui-Ying Zhang, Hai-Ying Dai, Ze-Hua Li, Ying Xiao, You-Zhi Hu, Xiao-Yu Huang, Kun Xing, Xin Jiang, Chao-Feng Liu, Jing An, Feng-Chun Li, Tao Tao, Jin-Fa Jiang, Ying Yang, Yao-Rong Dong, Lei Zhang, Guang Fu, Ying Li, Shu-Wei Huang, Li-Ping Dou, Lan-Jun Sun, Ying-Qiang Zhao, Jie Li, Yun Xia, Jun Liu, Fan Liu, Wen-Jin He, Ying Li, Jian-Cong Tan, Yang Lin, Ya-Bin Zhou, Jian-Fei Yang, Guo-Qing Ma, Hui-Jun Chen, He-Ping Liu, Zong-Wu Liu, Jian-Xiong Liu, Xiao-Jia Luo, Xiao-Hong Bin, Ya-Nan Yu, Hai-Xia Dang, Bing Li, Fei Teng, Wang-Min Qiao, Xiao-Long Zhu, Bing-Wei Chen, Qi-Guang Chen, Chun-Ti Shen, Yong-Yan Wang, Yun-Dai Chen, Zhong Wang

**Affiliations:** 1 Institute of Basic Research in Clinical Medicine, China Academy of Chinese Medical Sciences, Beijing, China; 2 Department of Cardiology, Chinese PLA General Hospital, Beijing, China; 3 Department of Cardiology, Dongfang Hospital Affiliated to Beijing University of Chinese Medicine, Beijing, China; 4 Department of Geratology, Peking University Third Hospital, Beijing, China; 5 Department of Cardiology, Xuan Wu Hospital, Capital Medical University, Beijing, China; 6 Department of Cardiology, Guang’anmen Hospital, China Academy of Chinese Medical Sciences, Beijing, China; 7 Department of Cardiology, First Affiliated Hospital of Henan University of Chinese Medicine, Zhengzhou, Henan China; 8 Department of Cardiology, Wuhan University Renmin Hospital, Wuhan, Hubei China; 9 Department of Cardiology, Wuhan University Zhongnan Hospital, Wuhan, Hubei China; 10 Affiliated Zhongshan Hospital of Dalian University, Dalian, Liaoning China; 11 Department of Cardiology, Second Affiliated Hospital to Hunan University of Chinese Medicine, Changsha, Hunan China; 12 Department of Cardiology, First Affiliated Hospital to Changchun University of Chinese Medicine, Changchun, Jilin China; 13 Department of Cardiology, First Affiliated Hospital to Shanxi Medical University, Taiyuan, Shanxi China; 14 Department of Cardiology, Affiliated Hospital of Shanxi University of Chinese Medicine, Xianyang, Shanxi China; 15 Department of Cardiology, Changsha Central Hospital, Changsha, Hunan China; 16 Department of Cardiology, Hubei Provincial Hospital of Traditional Chinese Medicine, Wuhan, Hubei China; 17 Department of Cardiology, Shanxi Provincial People’s Hospital, Xi’an, Shanxi China; 18 Department of Cardiology, Shanxi Province Hospital of Traditional Chinese Medicine, Xi’an, Shanxi China; 19 Department of Cardiology, Xi’an City Hospital of Traditional Chinese Medicine, Xi’an, Shanxi China; 20 Department of Cardiology, Shanghai Tongji Hospital, Shanghai, China; 21 Department of Cardiology, Shanghai Municipal Hospital of Traditional Chinese Medicine, Shanghai, China; 22 Department of Cardiology, The First Hospital of Changsha, Changsha, Hunan China; 23 Department of Cardiology, Xinhua Hospital of Zhejiang Province, Hangzhou, Zhejiang China; 24 Department of Cardiology, Second Affiliated Hospital of Tianjin University of Traditional Chinese Medicine, Zengchan Dao, Tianjin China; 25 Department of Chinese medicine, Shanghai Tenth People’s Hospital, Shanghai, China; 26 Department of Cardiology, Chongqing City Hospital of Traditional Chinese Medicine, Chongqing, China; 27 Department of Cardiology, Third People’s Hospital of Chongqing, Chongqing, China; 28 Department of Cardiology, First Affiliated Hospital of Heilongjiang University of Traditional Chinese Medicine, Harbin, Heilongjiang China; 29 Department of Cardiology, Second Affiliated Hospital of Heilongjiang University of Traditional Chinese Medicine, Harbin, Heilongjiang China; 30 Department of Cardiology, Jilin Province People’s Hospital, Changchun, Jilin China; 31 Department of Cardiology, Chengdu Second People’s Hospital, Chengdu, Sichuan China; 32 China Academy of Chinese Medical Sciences, Beijing, China; 33 Institute of Chinese Meteria Medica, China Academy of Chinese Medical Sciences, Beijing, China; 34 Beijing Genomics Institute (Shenzhen), Shenzhen, Guangdong China; 35 School of Public Health, Southeast University, Nanjing, Jiangsu China; 36 Changzhou Hospital of Traditional Chinese Medicine, Changzhou, Jiangsu China

**Keywords:** Cardiology, Drug development, Target identification, Clinical trials

## Abstract

It’s a challenge for detecting the therapeutic targets of a polypharmacological drug from variations in the responsed networks in the differentiated populations with complex diseases, as stable coronary heart disease. Here, in an adaptive, 31-center, randomized, double-blind trial involving 920 patients with moderate symptomatic stable angina treated by 14-day Danhong injection(DHI), a kind of polypharmacological drug with high quality control, or placebo (0.9% saline), with 76-day following-up, we firstly confirmed that DHI could increase the proportion of patients with clinically significant changes on angina-frequency assessed by Seattle Angina Questionnaire (ΔSAQ-AF ≥ 20) (12.78% at Day 30, 95% confidence interval [CI] 5.86–19.71%, *P* = 0.0003, 13.82% at Day 60, 95% CI 6.82–20.82%, *P* = 0.0001 and 8.95% at Day 90, 95% CI 2.06–15.85%, *P* = 0.01). We also found that there were no significant differences in new-onset major vascular events (*P* = 0.8502) and serious adverse events (*P* = 0.9105) between DHI and placebo. After performing the RNA sequencing in 62 selected patients, we developed a systemic modular approach to identify differentially expressed modules (DEMs) of DHI with the Z_summary_ value less than 0 compared with the control group, calculated by weighted gene co-expression network analysis (WGCNA), and sketched out the basic framework on a modular map with 25 functional modules targeted by DHI. Furthermore, the effective therapeutic module (ETM), defined as the highest correlation value with the phenotype alteration (ΔSAQ-AF, the change in SAQ-AF at Day 30 from baseline) calculated by WGCNA, was identified in the population with the best effect (ΔSAQ-AF ≥ 40), which is related to anticoagulation and regulation of cholesterol metabolism. We assessed the modular flexibility of this ETM using the global topological D value based on Euclidean distance, which is correlated with phenotype alteration (*r*^2^: 0.8204, *P* = 0.019) by linear regression. Our study identified the anti-angina therapeutic module in the effective population treated by the multi-target drug. Modular methods facilitate the discovery of network pharmacological mechanisms and the advancement of precision medicine. (ClinicalTrials.gov identifier: NCT01681316).

## Introduction

Chronic stable angina is a main manifestation in approximately half of all patients who present with coronary artery disease (CAD), which is a leading cause of death worldwide.^1^ With the realization that CAD involves perturbations of large complex biological networks,^2^ future success in managing CAD may shift from “reactive medicine”, which generally treats individual targeted items, e.g., LDL-C, to “proactive P4 medicine” (predictive, preventive, personalized, and participatory), in which disease mechanisms and systems biology allow for a multitargeted and patient-centric approach.^3–5^ However, for complex CAD interventions, particularly through polypharmacological drugs with multi-targets, it is especially difficult to detect the specific targets for the matched population. In the network pharmacology, the multi-targets on CAD or angina have been identified from the differentially expressed genes^6,7^ to the subnetwork with correlative genes.^8–10^ Therefore, in our study, the certain correlation between CAD and polypharmacological drug treatment should be evaluated with modular pharmacology beyond network pharmacology, which can connect the biological network to the pharmacological network based on the minimal functional unit modules.^11^ Modular flexibility can be adapted to different disease conditions in different people, and the various modular units themselves can be customized in a variety of ways to accommodate different interventions.^12^ Subsequently, it is possible to identify the effective therapeutic module of a multi-target drug in the responded population.

Danhong injection (DHI, made by Shandong Danhong Pharmaceutical Co. Ltd., with quality control via high-performance liquid chromatography (HPLC) fingerprinting (Supplementary Fig. S1) for over 90% similarity among the batches used in this trial) is a polycomponent drug, which contains 5 main compounds including Danshensu sodium, protocatechualdehyde, p-coumaric acid, rosmarinci acid, salvianolic acid B (Supplementary Fig. S2), exacted from two kind of herbal medicine, *Danshen* and *Honghua*. It is widely used in China due to its safety and efficacy in treating cardiovascular diseases.^13,14^ DHI was reported to have multiple pharmacological effects with multiple targets on CAD,^14^ including promoting endothelial progenitor cells (EPCs) mobilization via upregulating the expression of Akt, eNOS and MMP-9,^15^ attenuating atherosclerosis and macrophage lipid accumulation by promoting the activation of PI3K/AKT insulin signaling pathway,^16^ reducing the platelet aggregation^17^ as well as reducing oxidative stress and maintaining mitochondrial integrity via the Keap1/Nrf2/JNK pathway.^18^ Meanwhile, the main compounds, as Danshensu sodium, and salvianolic acids were also reported with polypharmacological effects against platelet aggregation and thrombus formation, promoting fibrin degradation, protecting against myocardial ischemia, and improving the microcirculation.^19,20^ Furthermore, a number of clinical studies also demonstrated that DHI might be an effective and safe treatment option to prevent angina attacks in the management of CAD.^13,21^ However, prior clinical studies generally provided low-quality evidence, and the molecular features of the targeted populations remained to be uncovered. Therefore, in this study, we conducted a well-designed, randomized, multicenter, double-blind, placebo-controlled trial with a prespecific selected 62-case RNA sequencing study to investigate the effect, safety and effective therapeutic module of DHI among patients with chronic stable angina.

## Results

### Danghong Injection could improve the angina-specific health status with favorable safety

**Fig. 1:**
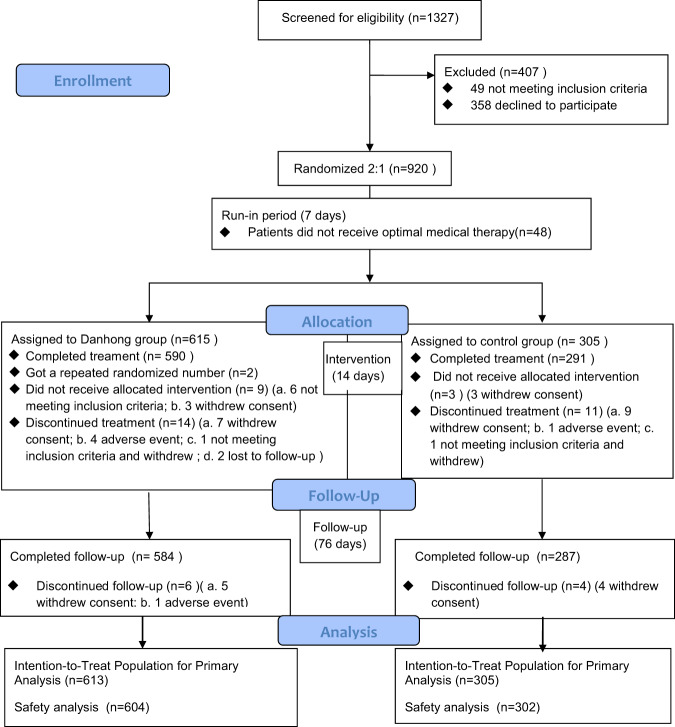
The flow chart of the trial

From December 2012 through October 2016, we screened 1327 patients at 31 centers in China; 615 eligible patients were randomly assigned to the DHI group, while 305 were assigned to the control group. Ultimately, 918 patients diagnosed with CAD using coronary angiography or computed tomography angiography were included in the “intention-to-treat” population for efficacy analysis, comprising 613 in the DHI group and 305 in the control group, since 2 in the DHI group received duplicate random numbers (Fig. 1). The distributions of demographic and clinical characteristics were reasonably well balanced between the two groups (Supplementary Table S1). The concomitant medications between the two groups during the 90 days showed no significance (*P* > 0.05) (Supplementary Table S2).

**Fig. 2:**
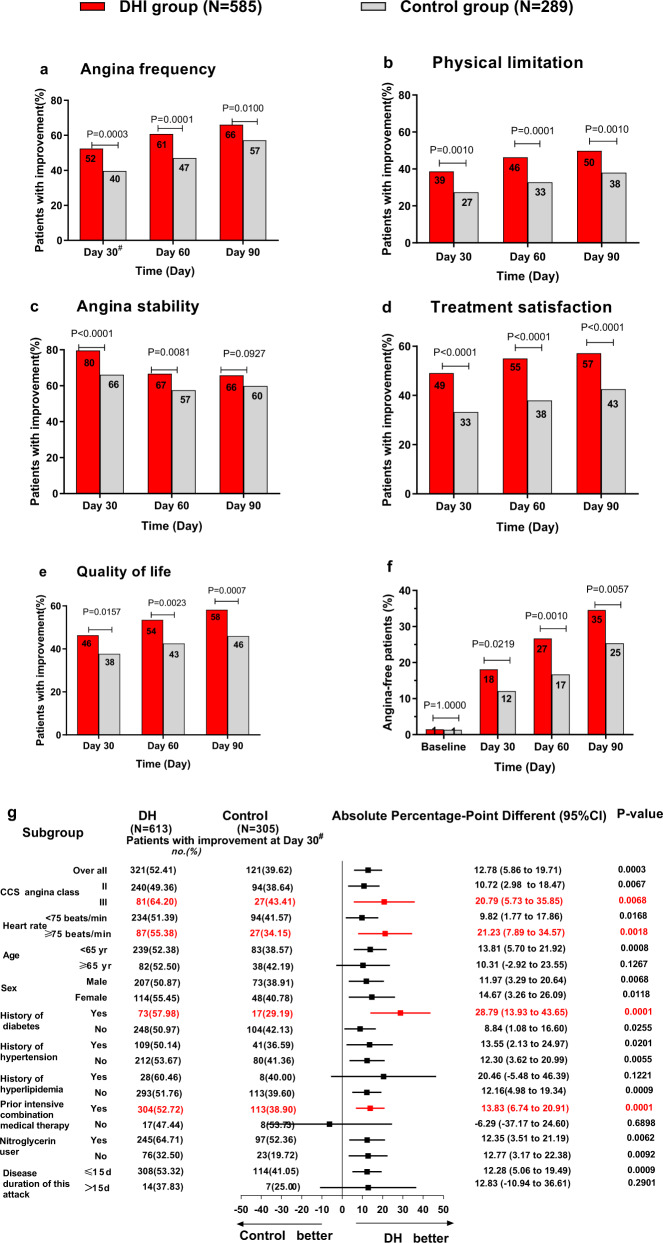
Changes in Seattle Angina Questionnaire scores. **a**–**e** Patients with clinically significant improvements from baseline in their Seattle Angina Questionnaire scores; **f** Freedom from angina over time as assessed by the angina frequency scale of the Seattle Angina Questionnaire (scored out of 100); **g** Forest plot of the primary outcome in prespecified subgroups (at Day 30). “#” in Figs. 1a and 1g indicates that the primary outcome was calculated from the imputed data, with *n* = 613 in the DHI group and *n* = 305 in the control group

Significant differences were observed in the proportions of patients with clinically significant changes on the domain “angina frequency” in Seattle Angina Questionnaire (SAQ-AF)^22^ at Day 30 (DHI vs. placebo: 52.41% vs. 39.62%; *P* = 0.0003; difference and its 95% CI: 12.78% (5.86%, 19.71%)), Day 60 (60.85% vs. 47.04%; *P* = 0.0001; difference and its 95% CI: 13.82% (6.82%, 20.82%)) and Day 90 (66.10% vs. 57.14%; *P* = 0.0100; difference and its 95% CI: 8.95% (2.06%, 15.85%)) (Fig. 2a and Supplementary Table S3). According to the three sensitivity analyses, the results of unadjusted and adjusted for center effects showed center factor did not affect the treatment effects, and the results were all robust to departure from missing at random missing at random or to use the mixed-effect model with repeated measures method (Supplementary Table S4). Compared with the placebo group, an increased proportion of patients in the DHI group had clinically significant improvements in physical limitation (PL), treatment satisfaction (TS), and quality of life (QL) from Day 30 through Day 90 (*P* < 0.01), but in angina stability (AS), these differences were no longer significant at Day 90 (*P* = 0.0927) (Fig. 2b–e and Supplementary Table S3). We also found that 18.2% of patients became free of angina (i.e., had a SAQ-AF score of 100) in the DHI group during the first 30 days, with continuous improvement to 34.59% thereafter at Day 90, which was significantly higher than the proportion in the control group from Day 30 through Day 90 (*P* = 0.0219–0.0057) (Fig. 2f and Supplementary Table S5). Moreover, the results of the subgroup analysis were generally consistent with those obtained from the entire study population (Supplementary Fig. 2g). In all domains of the SAQ, the scores were consistently higher in the DHI group than in the control group at all posttreatment time points (*P* < 0.0001 ~ *P* = 0.0193) (Supplementary Table S6, Fig. S3).

**Fig. 3:**
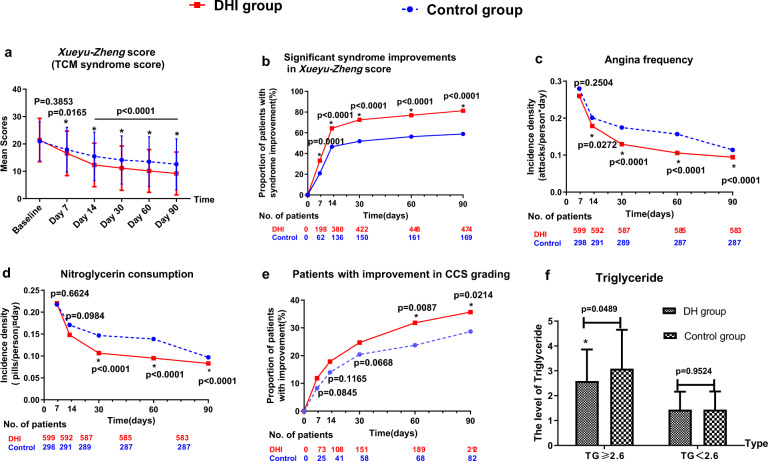
Changes in other secondary outcomes between the two groups. **a** Comparison of mean *Xueyu-Zheng* scores at each time point. **b** The proportion of patients with significant improvements in *Xueyu-Zheng* scores (%). **c** Changes in the incidence density of angina (attacks/person-day) according to patients’ diaries. **d** Changes in the incidence density of nitroglycerin consumption according to patients’ diaries. **e** The proportion of patients with normal ECG recordings. **f** The proportion of patients with improved CCS grades

More dramatic improvements in the *Xueyu-Zheng* score^23^ were observed in the DHI group than in the control group from Day 7 (*P* = 0.0165) to Day 90 (*P* < 0.0001) (Fig. 3a and Supplementary Table S7). We also found that the proportion of patients with significant improvements in the *Xueyu-Zheng* score was significantly higher in the DHI group than in the control group from Day 7 to Day 90. (*P* ≤ 0.0001, Fig. 3b and Supplementary Table S7). According to the patients’ diaries, the change in the cumulative incidence density of angina was significantly greater in the DHI group than in the placebo group, beginning from the second week (Days 8–14) (DHI vs. placebo: 0.18 attacks/person-day vs. 0.20 attacks/person-day, *P* = 0.0272) until Day 90 (*P* < 0.0001, Fig. 3c and Supplementary Table S8), and a similar trend was observed in the cumulative consumption density of nitroglycerin from Day 15 to Day 90 (*P* < 0.0001) (Fig. 3d and Supplementary Table S8). The proportion of patients with improvement in Canadian Cardiovascular Society (CCS) class also increased in both groups, but significant differences between the two groups were observed at Day 60 and Day 90 (*P* = 0.0087 and 0.0214, respectively, Fig. 3e and Supplementary Table S9). Significant differences in the mean triglyceride level at Day 14 were observed between the two groups in the population with a triglyceride level ≥2.6 mmol/L (*P* = 0.0489, Fig. 3f). However, no significant differences were found between the two groups regarding the proportion of patients with normal electrocardiogram (ECG) recordings (Supplementary Table S9), total cholesterol, high-density lipoprotein (HDL) cholesterol, low density lipoprotein (LDL) cholesterol, population-wide triglyceride, hypersensitive C-reactive protein (hs-CRP), C-reactive protein (CRP), or platelet aggregation rate (PAR) at Day 14 (*P* = 0.1222–0.9495) (Supplementary Table S10).

Among the 906 patients who received the study drug, 60 patients reported 93 adverse events (AEs) in the DHI group, and 36 patients reported 58 AEs in the control group; the incidence of AEs did not differ significantly between the two groups (*P* = 0.3597), and the same was true of the incidence of severe adverse events (SAEs) (*P* = 0.9105). In addition, 4 patients reported new-onset major vascular events within 90 days (0.5%) in the DHI group and 1 (0.3%) in the control group (*P* = 0.8502), and no severe or moderate hemorrhages or deaths were reported within 90 days (Supplementary Table S11).

### The changes of differentially expressed genes did not reflect the perturbation of the network by the multi-target drug

**Fig. 4:**
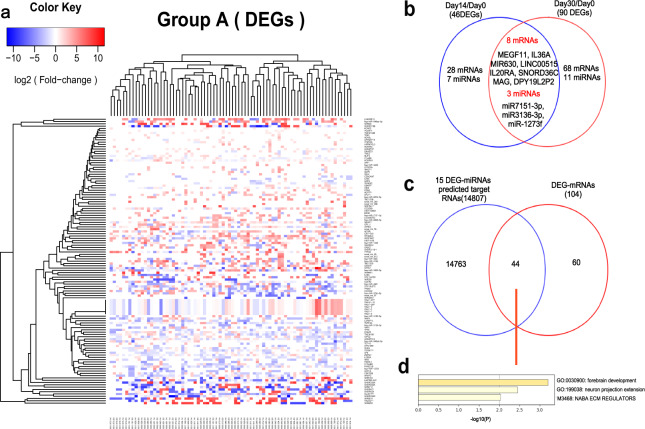
Differentially express genes targeted by Danhong Injection. **a** Heatmap of the gene expression pattern (miRNA and mRNA) of the DHI group. Red boxes represent the genes that were upregulated after treatment, while blue ones represent downregulated. **b** Venn diagram of the numbers of differentially expressed genes (DEGs) before and after treatment. The overlapping area represents the number of genes found to be differentially expressed both at Day 14 or Day 30 after treatment. **c** Venn diagram of 104 DEG-mRNAs and the predicted target mRNAs of 15 DEG-mRNAs. **d** The GO biological process categories of the 44 overlapping DEG-mRNAs and the predicted target mRNAs of 15 DEG-mRNAs

Sixty-two patients agreed to provide serum samples for RNA sequencing (28 from 301 Hospital and 34 from Xuanwu Hospital; their clinical characteristics are shown in Supplementary Table S12), including 41 from the DHI group and 21 from the control group. There were 125 differentially expressed genes (DEGs) (75 upregulated and 50 downregulated) in response to DHI treatment, of which 104 mRNAs and 21 non-coded miRNAs (Day 14 or Day 30) (Fig. 4a and Supplementary Table S13), compared with the control group. The enriched GO biological processes (GO-BPs) of these 104 mRNAs were not related to CAD (Supplementary Fig. S4). Eleven DEGs, including 8 mRNAs and 3 miRNAs, were significantly expressed on both Day 14 and Day 30 (Fig. 4b). However, none of these common DEGs was reported to be associated with stable angina. Furthermore, among the 21 miRNAs, 15 were mapped onto identified names via miRBase,^24^ and the 6 novel miRNAs were predicted by miRDeep2.^25^ We identified 14807 genes as the predicted target genes regulated by these 15 miRNAs via TargetScan Release 7.2,^26^ and 44 overlapped with the 104 mRNAs encoded by the DEGs (Fig. 4c); these 44 genes were identified by Metascape^27^ as related to forebrain development (GO: 0030900) and neuron projection extension (GO: 1990138) (Fig. 4d), meaning that they were not likely to be functionally related to stable angina. Here, we found that the changes of DEGs did not reflect the perturbation of the network targeted by DHI.

### The multi-target drug Danhong injection treated the angina through a 25-targeted-functional module (TFM) network

**Fig. 5:**
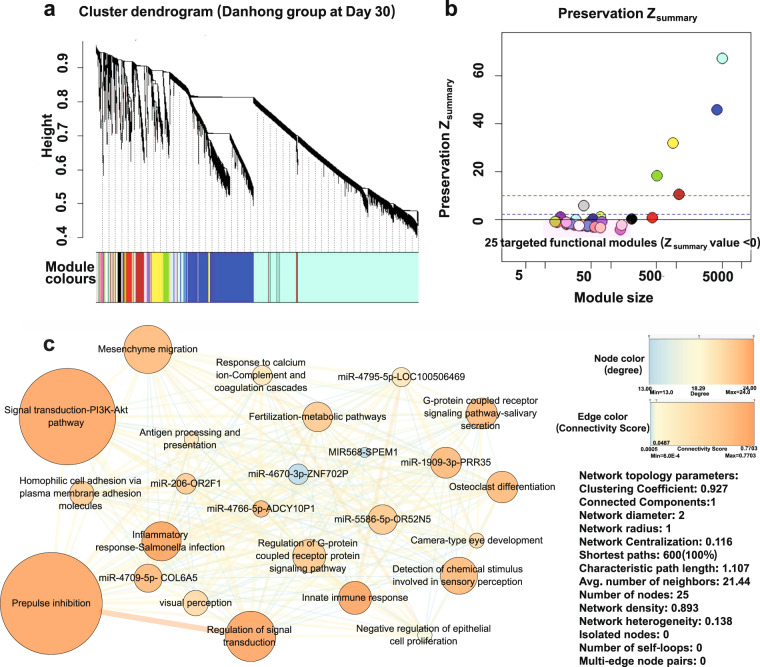
Targeted modular map by the multi-target drug Danhong Injection. **a** The hierarchic cluster tree (dendrogram) of DHI. Each major tree branch represents a module, and each module is labeled with a color below the dendrogram. **b** The distribution of modules by Z_summary_ value. There were 25 differentially expressed modules (DEMs) with negative Z_summary_ values (<0); these modules were considered the targeted functional modules (TFMs). **c** The 25-TFM map for DHI at Day 30 with the 25 TFMs as nodes and the connectivity score (CS) as the edge between each pair of modules. The node size indicates the number of genes in the corresponding TFM, and the color of the node shows the degree of the TFM in the network

Using WGCNA,^28^ we detected 38 modules from the coexpressed gene network in the DHI group at Day 30 (Fig. 5a), and then identified 25 differentially expressed modules (DEMs) with Z_summary_ values^29^ less than 0 for DHI compared to placebo (Fig. 5b and Supplementary Table S14), as the targeted modules of DHI. Then, the targeted module network of DHI was constructed, with 25 nodes (DEMs) and 268 edges (connectivity scores, CSs),^30^ which is a high-density small-world network (0.893) with a characteristic path length of 1.107 and a clustering coefficient of 0.927 (Fig. 5c and Supplementary Table S15), enriched for 88 GO-BPs and 16 KEGG pathways (Supplementary Fig. S5). Additionally, after determining the functions of the 25 targeted modules from the hub genes or the representative GO-BP and KEGG pathways, which were named “targeted functional modules” (TFMs), (Fig. 5c, Supplementary Tables S14, S16 and Fig. S5), we found that the “signal transduction-PI3K-Akt signaling pathway” TFM was correlated with anti-inflammation,^31^ regulation of endothelial cell function^32^ and atherosclerotic plaque stability^31^; the “*miR-206* & *OR2F1*” TFM is related to regulation of cholesterol metabolism^33^ and endothelial cell function.^34^

### Globle flexibility of the effective therapeutic module in different responsed populations

**Fig. 6:**
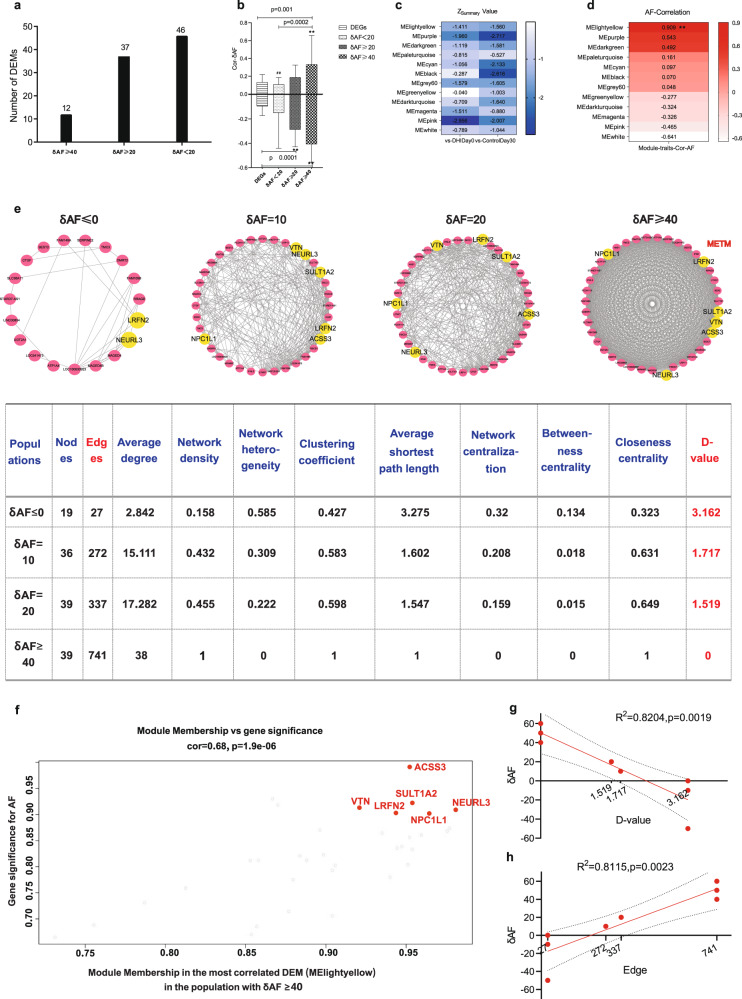
The effective target module detected in the responsive population. **a** The numbers of differentially expressed modules (DEMs) with negative Zsummary values (<0) according to WGCNA in the populations with different responses to DHI treatment at Day 30. ΔAF indicates the change in the Seattle Angina Questionnaire angina frequency scale (SAQ-AF) score from baseline (Day 0) to Day 30. **b** Comparison of the correlation with ΔAF among DEGs and DEMs in different populations. The significance of the difference between each pair of groups was calculated by ANOVA; ** indicates the notable statistical significance of the correlation with ΔAF between DEGs and the DEMs in the populations with ΔAF ≥ 40, and ## indicates the notable statistical significance of the correlation with ΔAF between the DEMs in the populations with ΔAF ≤ 0 and ΔAF ≥ 40. **c** Heatmap of the Zsummary value pattern of the 12 DEMs at Day 30 in the populations with ΔAF ≥ 40 compared with the baseline (Day 0) and the control group. **d** Heatmap of correlation with ΔAF for the 12 DEMs at Day 30 in the populations with ΔAF ≥ 40. **e** The modular flexibility of the effective therapeutic module in the populations with different responses to DHI treatment. The topological parameters are shown in the table. **f** The gene distribution in the effective therapeutic module shows the correlation between gene significance to AF and module membership. **g**, **h** The linear regression correlation between D value (**g**) or edge (**h**) and ΔAF

Twelve, 37, and 46 DEMs were identified in the populations with the best effect (ΔAF ≥ 40), a mild effect (40 > ΔAF ≥ 20) and no effect (ΔAF < 20), respectively (Fig. 6a, c and Supplementary Fig. S6). The correlation of 90 DEGs with ΔAF at Day 30 was somewhat weak, ranging from −0.4574 to 0.3416 (Supplementary Table S17), which was significantly weaker than the correlation of DEMs with ΔAF in the population with the best effect or a mild effect (Fig.  6b, d and Supplementary Fig. S7). The module with the highest correlation value (0.909, *p* = 0.0046) in the population with the best effect was identified as the most effective therapeutic module (METM) (Fig. 6d). This METM had 39 nodes, 741 edges and a high network density (Network density:1) (Fig. 6e) and was related to the negative regulation of blood coagulation (GO:0030195, *p* = 0.0184) (Supplementary Table S18). The topology of modules constructed with the genes in the METM was completely different among different populations (Fig. 6e). The D value, the global topological similarity to the METM, decreased as the effect increased (*r*^2^ = 0.8204, *p* = 0.0019, Fig. 6e, f), while the number of edges in the flexible modules increased with the effect (*r*^2^ = 0.8115, *p* = 0.0023, Fig. 6e, h). Six genes, *ACSS3, SULT1A2, VTN, LRFN2, NPC1L1*, and *NEURL3*, were identified as being highly significant to AF and having high module membership (Fig. 6f), which existed in the population with an effect (ΔAF ≥ 10) but not in the population with no effect (ΔAF ≤ 0) (Fig. 6e). It has been reported that genetic variation in *NPC1L1* is associated with a reduction in the risk of ischemic vascular disease, with a corresponding reduction in LDL cholesterol.^35^

## Discussion

In our study, we found that although 94.44% (867/918) patients had the prior optimal medical therapy for angina, they all had over moderate angina (CCS II-III), and even 189 patients encountered relatively severe angina (CCS III). Our study indicated that 14-day usage of DHI could significantly reduce the number of angina episodes and improve angina-specific health status for at least 90 days, particularly for patients with severe angina (CCS class III), faster heart rate (HR ≥ 75 beats/min) or diabetes (Fig. 2g), who are most prone to refractory angina. We confirmed the additional benefit and safety of DHI when added to optimal medical therapy in patients with symchronic stable angina. The effect of the DHI in improving patients’stable CAD–specific health status was similar to that of percutaneous coronary intervention in the first 3 months.^22^ In addition, DHI did not bring any additional safety risk as an add-on therapy for patients with symptomatic angina.

This study is the first rigorously designed randomized controlled trial to evaluate the efficacy and safety of DHI in the management of symptomatic chronic stable angina. In this study, the central online randomization system, double blinding, the use of a placebo control and the role of the independent DMC may prevent potential selection, performance and detection biases. Therefore, the integrity and reliability of the data validate the concept that DHI is a promising therapeutic option for the treatment of chronic stable angina, which is also generally consistent with the findings of a previous small-sample clinical study.^21^

Furthermore, we firstly sketched the 25-targeted functional modular map and then detected a flexible effective therapeutic module of DHI related to anticoagulation and the regulation of cholesterol metabolism^35^ with topological features that change with phenotype alteration, based on the analysis of DEMs rather than DEGs. We believe that the population with this module will achieve better drug effects than the population with any single DEG. The methodology of modular pharmacology used in our study was more suitable and efficient than network analysis of DEGs or pathways in uncovering the mechanism of multi-target drugs. As a module is generally considered a closely linked unit that performs biological functions at the network level, drug-induced network rewiring may lead to changes in modular structure.^36^ Thus, analysis of DEMs must be especially useful in clarifying the mechanism of multi-target drugs and detecting the effective therapeutic module underlying their effects.

Besides, although millennia-old Chinese medicine treats disease with many combination therapies involving diverse ingredients used in clinic practice, the robust evidence for their effects and the underlying mechanism is far from enough. In our study, we provides an applicable paradigm for the future study of the similar Chinese medicine product with multi-targets on a certain disease with therapeutic advantages. It will be helpful to confirm the effect, uncover the therapeutic mechanism and facilitate the modernization of Chinese medicine.

We are also aware of a possible weakness of the study. Only 4 new-onset major vascular events and no deaths were reported in the DHI group during the 90-day follow-up; this period may not have been long enough to detect these endpoints and confirm the benefits of DHI. Future studies should be designed with longer follow-up periods.

In conclusion, an initial short-term course of DHI along with optimal medical therapy relieved angina and improved self-assessed health status to a greater extent than an initial strategy of optimal medical therapy alone over a follow-up of approximately 90 days. The effect might correlate with an effective therapeutic module related to anticoagulation and the regulation of cholesterol metabolism. Our study provides a novel strategy to detect the effective therapeutic module of multi-target drugs based on modular flexibility in differentially responsive populations.

## Materials and methods

### Study design

This study was an adaptive-design, randomized, multicenter, double-blind, placebo-controlled trial. The trial protocol^23^ was approved by the central institutional review board from Chinese PLA General Hospital (IRB No. [2012] Pharmaceutical (025)). This trial complied with the principles of the Declaration of Helsinki. Informed written consent was obtained from all participants. Patients or the public were not involved in the design, or conduct, or reporting, or dissemination plans of our research.

### Participants

Symptomatic patients aged 18–70 years who were diagnosed with CAD and “*Xueyu-Zheng*”, a score of at least 15 on the Chinese Medicine Symptom Scale of “*Xueyu-Zheng*” for angina patients,^23^ and had CCS class II or III angina were enrolled in this study. Key exclusion criteria included a history of myocardial infarction within the past 3 months; severe complications such as liver or renal dysfunction, severe cardiopulmonary dysfunction, etc.; a history of epilepsy or cerebral hemorrhage; a history of drug-induced bleeding or hematopoietic disorder; patients who underwent a surgery within the past 4 weeks or those who had a hemorrhagic tendency.

### Interventions

Optimal medical therapy was given to all participants throughout the trial, including antiplatelet agents (aspirin and/or clopidogrel), lipid-lowering agents (atorvastatin or simvastatin) and anti-angina agents (β-blockers, long-acting nitrates, or calcium channel blockers). All of these basic treatments were recorded in detail on electronic case report forms (eCRFs). Participants in the DHI and control groups received a daily intravenous drip of 40 ml DHI and 40 ml placebo (0.9% normal saline), respectively, added to 250 ml 0.9% normal saline. All participants underwent 2 weeks of treatment and 76 days of follow-up. Patients could take a nitroglycerin tablet (0.5 mg per tablet, provided by Beijing Yimin Pharmaceutical Co., Ltd.) in the event of an angina attack, and they recorded the details regarding the angina attack and the usage of nitroglycerin in a patient diary. Other drugs with the same indications as DHI were not allowed to be used throughout the study period. All concomitant medications were recorded on the eCRFs.

### Outcome measurements

Angina-specific health status was assessed at baseline and on days 30, 60, and 90. Each assessment was performed using the SAQ, a 19-item questionnaire with 5 domains, namely, physical limitation (PL), angina stability (AS), angina frequency (AF), treatment satisfaction (TS), and quality of life (QL), each scored on a scale from 0 to 100, where a higher score indicates a better health status (13). A clinically significant change on the SAQ was defined as a difference of 8 points or more on PL, 25 or more on AS, 20 or more on AF, 12 or more on TS, or 16 or more on QL.^22^ The primary outcome was the proportion of patients with a clinically significant change in SAQ-AF at Day 30. The proportions of patients who experienced clinically significant changes in the other four SAQ domains were used as secondary outcome measures. Other secondary outcomes included the Chinese Medicine Symptom Scale of “*Xueyu-Zheng*”;^23^ frequency of angina attacks; consumption of short-acting nitrates; improvements in CCS class and electrocardiogram recordings; changes in biochemical indexes such as serum lipids, hs-CRP or CRP and PAR at Day 14; the incidence of new-onset major vascular events; overall mortality within 90 days; the frequency of adverse events (AEs); and the incidence of moderate or severe hemorrhages within 90 days, details are provided in the protocol.^23^

Another outcome measure, the change of total exercise duration (TED) in exercise tolerance test (ETT) from baseline to Day 14, was assessed in 290 patients until the first interim analysis. However, after the first interim analysis, the Data Monitoring Committee (DMC) recommended that the ETT test be omitted for better patient compliance and higher feasibility of the trial.

### Randomisation and blinding

Eligible participants were randomly assigned to either the DHI group or the control group in a 2:1 ratio, with a block size of 6, via a central randomization system (Brightech, Somerset, USA) This system automatically randomized patients and generated a randomization number with a message noting their assigned treatment. Randomization was stratified based on whether a patient received standard conventional therapy for more than 1 week prior to study initiation.

Since the color of DHI and 0.9% saline are different, the dropping bottles were wrapped in sealed shaded bags, and brown infusion devices were used for infusion. These procedures were implemented by two professional nurses who were required to sign a confidentiality agreement before study initiation and not to contact each other. One of the professional nurses was in charge of preparing the drugs in a special transfusion room and sealing the infusion bottles with shaded brown bags. The other nurse took the prepared drugs from the transfusion room to the infusion nurse and supervise the infusion process to ensure that the allocation of the drugs is blinded to the patients. The participants, outcome assessors, and statisticians were blinded to treatment allocation.

### Statistical analysis

Using EAST5.2, we initially estimated that at least 870 patients needed to be enrolled in the study in order to achieve 85% power to detect a 10% difference in the primary outcome between the DHI and control groups at Day 30,^22^ given a significance level of 5% and a dropout rate of 20%. Two interim analyses with O’Brien-Fleming^37^ stopping boundaries were performed to re-estimate the sample size after 288 and 582 patients completed the trial. The sample size was adjusted to 920 by DMC after the second interim analysis.

The primary outcome was analyzed according to the intention-to-treat principle. Missing data were handled by regression-based multiple imputation using the fully conditional method.^38^ Three sensitivity analyses (1 preplanned and two post hoc) were conducted for the primary outcome. The preplanned used control-based pattern model to evaluate sensitivity to missing data departure from the missing at random assumption. Two post hoc sensitivity analyses evaluated whether baseline imbalance in clinical site and a mixed-effect model with repeated measures could have diluted the estimates of treatment effect. Continuous variables were reported as the mean and standard deviation (SD), and categorical variables were reported as frequencies and percentages with 95% CIs. Statistical differences between groups were analyzed using Student’s *t*-test or the Wilcoxon rank-sum test for quantitative data and chi-squared test for categorical data. All reported *P* values were two-sided, and a *P* value less than 0.05 was considered to be statistically significant except in the primary outcome analyses, in which Bonferroni’s multiple comparison test was applied twice and *P* < 0.016 was considered significant. All calculations were performed using SAS 9.4 (SAS Institute Inc., USA).

### Analysis of RNA profile and targeted modules

The serum samples of patients from 301 Hospital and Xuanwu Hospital were sequenced using the Illumina HiSeq sequencing platform according to the manufacturer’s instructions and previous studies described.^39,40^ Significant differentially expressed genes (DEGs) were defined as genes with a *P* value <0.05 and at least a 1.5-fold change. The construction of the gene coexpression network for DHI at Day 30 and the identification of modules were implemented using WGCNA, an R package for weighted correlation network analysis.^28^ A module with a Z_summary_ value less than 0 compared with the control group was defined as a differentially expressed module (DEM).^29^ Furthermore, we reconstructed the targeted DEM network using the connectivity score (CS)^30^ among these modules. Each targeted DEM was assigned a certain function, using its hub genes with the highest degree or the most significant Gene Ontology (GO) and Kyoto Encyclopedia of Genes and Genomes (KEGG) pathways enriched using the Database for Annotation, Visualization and Integrated Discovery (DAVID) Bioinformatics Resources 6.8;^41^ these modules were designated as targeted functional modules (TFMs). To further determine the molecular features of the targeted population, we divided the populations in the DHI group at Day 30 into 3 groups according to the phenotype alteration, ΔAF, defined as the change in SAQ-AF at Day 30 from baseline: the group with the best effect (ΔAF ≥ 40), a group with a mild effect (40 > ΔAF ≥ 20) and a group with no effect (ΔAF < 20). Significant DEMs were defined as those with a correlation value (*r*) ≥ 0.3 and a *P* value <0.05 as calculated by WGCNA,^28^ and the most significant DEM (with the highest r and lowest P value) in the population with the best effect was considered the most effective therapeutic module (METM) of DHI. The flexibility of the METM in differentially responsive populations was assessed with the global topological parameter D, based on Euclidean distance.^42,43^

## Supplementary information

Supplement Appendix-Trial Protocol &Statistical Analysis Plan

Supplemental Material

## Data Availability

The data that support the findings of this study have been deposited in the CNSA (https://db.cngb.org/cnsa/) of CNGBdb with accession number CNP0000461.
