# Supplementary material for: Detection of an anti-angina therapeutic module in the effective population treated by a multi-target drug Danhong injection: a randomized trial
Source: Signal Transduct Target Ther. 2021 Sep 1;6:329. doi: 10.1038/s41392-021-00741-x (PMC8410855; doi:10.1038/s41392-021-00741-x)
Supplement: Supplementary file 1 — Supplement Appendix-Trial Protocol &Statistical Analysis Plan [file 41392_2021_741_MOESM1_ESM.pdf]

Protocol &Statistical Analysis Plan

This supplement contains the following items:

1. Original protocol (Page 1-36)
2. Final protocol (Page 37-79)
3. Summary of changes in the protocol (Page 80)
4. Original statistical analysis plan (Page 81-101)
5. Final statistical analysis plan (Page102-125)
6. Summary of changes in the SAP (Page 126)

Protocol (Original)

The Original Version: April 24, 2012

Danhong Injection for the treatment of chronic stable angina (Phase IV): an adaptive-design, randomized, multi-center, double-blind, placebo-controlled clinical trial

Sponsored by: China National Key Technologies R&D Program on "New Drug R&D" (2011ZX09304-07)

EXECUTIVE COMMITTEE CHAIR

Prof. Yong-Yan Wang, Academician of Chinese Academy of Engineering

Institute of Basic Research in Clinical Medicine, China Academy of Chinese Medical Sciences

No.16 Nanxiaojie, Dongzhimennei, Beijing, 100700, China.

Tel: 86-10-84046033

E-mail: wangyongyan2010@sina.cn

PRINCIPLE INVESTIGATORS

Prof. Zhong Wang, Ph.D (for grant)

Institute of Basic Research in Clinical Medicine, China Academy of Chinese Medical Sciences

No.16 Nanxiaojie, Dongzhimennei, Beijing, 100700, China.

Tel:86-10-64093305

Email: zhonw@vip.sina.com

Prof. Yun-Dai Chen, M.D. (for trial)

Department of Cardiology, Chinese PLA General Hospital

No. 28 Fuxing Road, Beijing, 100853, China.

E-mail: cyundai@medmail.com.cn

PARTICIPATING SITES AND INVESTIGATOR

1. **Chinese PLA General Hospital**

Prof. Yun-Dai Chen, M.D. (for trial)

Site Principal Investigator, Director of Department of Cardiology

Chinese PLA General Hospital

No. 28 Fuxing Road, Beijing, 100853, China

E-mail: cyundai@medmail.com.cn

Prof. Wei Dong

Co-investigator, Department of Cardiology

Chinese PLA General Hospital

No. 28 Fuxing Road, Beijing, 100853, China

E-mail: 301dongw@sina.com

Dan-Dan Li

Co-investigator, Department of Cardiology

Chinese PLA General Hospital

No. 28 Fuxing Road, Beijing, 100853, China

E-mail: Ametuofo980869@163.com

Yu-Qi Liu

Co-investigator, Department of Cardiology

Chinese PLA General Hospital

No. 28 Fuxing Road, Beijing, 100853, China

E-mail: 7399253@qq.com

2. Dongfang Hospital Affiliated to Beijing University of Chinese Medicine

Prof. Yang Wu, M.D.

Site Principal Investigator, Director of Department of Cardiology

Dongfang Hospital Affiliated to Beijing University of Chinese Medicine

No. 6, Zone 1 Fangxinyuan, Fangzhuang, Beijing, 100078, China

E-mail:drwuyang@163.com

Da-Xuan Tang, M.D.

Co-investigator, Department of Cardiology

Dongfang Hospital Affiliated to Beijing University of Chinese Medicine

No. 6, Zone 1 Fangxinyuan, Fangzhuang, Beijing, 100078, China

E-mail: tang_dax@163.com

3. Peking University Third Hospital

Prof. Fu-Chun Zhang

Site Principal Investigator, Director of Department of Cardiology

Peking University Third Hospital

No.49, Huayuan North Road, Beijing, 100191, China

E-mail: Zhang.fuchun @medmail.com.cn

4. Xuan Wu Hospital, Capital Medical University

Prof. Qi Hua, M.D.

Site Principal Investigator, Director of Department of Cardiology

Xuan Wu Hospital, Capital Medical University

No.45, Changchun Street, Beijing, 100053, China

E-mail: huaqi5371@medmail.com.cn

5. Guang'anmen Hospital, China Academy of Chinese Medical Sciences

Prof. Jun Li, M.D.

Site Principal Investigator, Director of Department of Cardiology

Guang'anmen Hospital, China Academy of Chinese Medical Sciences

No.5, Beixiange, Beijing, 100053, China

Email: 13051458913@163.com

6. The First Affiliated Hospital of Henan University of T.C.M.

Prof. Ting-Hai Du

Site Principal Investigator, Director of Department of Cardiology

The First Affiliated Hospital of Henan University of T.C.M.

No. 19, Renmin Road, Zhengzhou, 450000, Henan, China

Email: dth2010@163.com

DATA MONITORING COMMITTEE (DMC)

Prof. Chun-Ti Shen (Chair), Changzhou TCM Hospital Affiliated to Nanjing University of Chinese Medicine

Prof. Qi-Guang Chen (Member, independent statistician), Southeast University

Prof. Hua-Qiang Zhang (Member), Jiangsu Provincial Commission of Health and Family Planning

Dr. Hong-Wu Wang (Member), Tianjin University of Traditional Chinese Medicine

Dr. Xiao-Chun Sun (Member), Beijing Kelunrui Pharmaceutical Technology Co. Ltd.

Prof. Cheng-Shu Wang (Member), Zhongda Hospital Southeast University

Dr. Hai-Xia Dang (Member), China Academy of Chinese Medical Sciences

Dr. Jun Liu (Member), Institute of Basic Research in Clinical Medicine, China Academy of Chinese Medical Sciences

PROTOCOL STATISTICIANS

Prof. Bi-Wei Chen, Ph.D., School of Public Health, Southeast University

Dijia Qiao 87, Nanjing,210009, Jiangsu,China

E-mail: drchenbw@126.com

TABLE OF CONTENTS

EXECUTIVE COMMITTEE CHAIR - 36 -
PRINCIPLE INVESTIGATORS - 36 -
PARTICIPATING SITES AND INVESTIGATOR - 37 -
DATA MONITORING COMMITTEE (DMC) - 45 -
PROTOCOL STATISTICIANS - 45 -
PROTOCOL SYNOPSIS - 49 -
1. BACKGROUND INFORMATION - 52 -
2. OBJECTIVES - 53 -
3. STUDY DESIGN - 53 -
3.1 Overview - 53 -
3.2 Sample size - 53 -
3.3 Randomization - 55 -
3.4 Blinding - 55 -
3.5 Interim analysis - 55 -
4. SUBJECTS - 56 -
4.1 Diagnostic criteria - 56 -
4.2 Inclusion Criteria - 58 -
4.3 Exclusion Criteria - 58 -
4.4 Withdrawal/dropout criteria - 59 -
4.5 Criteria for study termination - 60 -
4.6 Criteria for subject discontinuation - 60 -
5 INTERVENTION - 61 -
5.1 Optimal medical therapy - 61 -
5.2 Treatment regimens - 62 -
5.3 Duration and follow-up - 62 -
5.4 Concomitant medications - 62 -
6 OUTCOMES MEASUREMENTS - 63 -
6.1 Primary outcome - 63 -
6.2 Secondary Outcomes - 63 -
6.3 Other Pre-specified Efficacy Outcomes - 66 -
6.4 Safety Outcomes - 66 -
6.5 Study Schema - 67 -
7 ADVERSE EVENTS - 68 -
7.1 Definition - 68 -
7.2 Recording - 69 -
7.3 Severity of an AE - 69 -
7.4 Evaluation the correlation between AEs and study drugs - 70 -
7.5 Handling of SAEs - 70 -
7.6 Opening and handling of emergency envelops - 71 -
7.7 Follow-up of unresolved AEs - 71 -
8. DATA COLLECTION AND MANAGEMENT - 71 -
8.1 Establishment of Three Committees - 71 -
8.2 Data Management - 72 -

8.3 Data Report - 72 -
8.4 Data Auditing and Blinding Review - 73 -
8.5 Database Locking - 73 -
9. STATISTICAL CONSIDERATIONS - 74 -
9.1 Study Populations - 74 -
9.2 Statistical Analyses - 74 -
9.3 Statistical Analysis Plan (SAP) - 75 -
10 ETHICAL ASPECTS - 75 -
REFERENCES - 76 -

PROTOCOL SYNOPSIS

TITLE:

Danhong Injection for the treatment of chronic stable angina (Phase IV): an adaptive-design, randomized, multi-center, double-blind, placebo-controlled clinical trial

DESIGN:

A multi-center, randomized, parallel-controlled, double-blind, adaptive clinical trial (superiority design)

NUMBER OF SITES:

31 sites

SAMPLE SIZE:

A total of 870 study participants with angina pectoris was planned initially.

POPULATION:

Patients aged 18 to 70 years who suffered from chronic stable angina with a clinical diagnosis of “Xueyu-Zheng” (Blood Stasis Syndrome), CCS angina class Ⅱ or Ⅲ were eligible for inclusion in the study.

RANDOMIZATION：

Randomization to Danhong Injection (DHI) group or control group (0.9% normal saline as placebo) will be in a 2:1 ratio

REGIMEN:

Optimal medical therapy (OMT) was given to all participants throughout the trial, including anti-platelet agents (aspirin, 75-100 mg, once a day, and/or clopidogrel, 75 mg, once a day), lipid-lowering agents

(atorvastatin, 10-20 mg, once a day, or simvastatin, 20-40 mg, once a day) and anti-angina agents (β-blockers, long-acting nitrates, or calcium channel blockers).

DHI group: OMT + DHI (40 ml, qd) plus 0.9% normal saline (250 ml, ivgtt, qd)

Control group: OMT + placebo of DHI (0.9% normal saline, 40ml, qd) plus 0.9% normal saline (250 ml, ivgtt, qd)

TREATMENT DURATION:

14 days

FOLLOWING UP

76 days after treatment

OUTCOME MEASURES:

Efficacy:

The primary efficacy endpoint of this study is:

Proportions of patients who had a clinically significant change on the angina-frequency scale in the Seattle Angina Questionnaire (SAQ) at Day 30

Secondary efficacy endpoints are:

(1).The total score of symptoms in a questionnaire related to traditional Chinese medicine;

(2). The proportion of patients who experience clinically significant changes in the other four Seattle Angina Questionnaire domains;

(3) The frequency of anginal attacks;

(4) Angina grade, according to the Canadian Cardiovascular Society (CCS) Angina Grading Scale;

(5) Consumption of short-acting nitrates;

(6) Changes in electrocardiogram results;

(7) Changes in serum lipid levels;

(8) Changes in high-sensitivity C-reactive protein levels;

(9) Changes in the platelet aggregation rate.

(10) Incidence of new-onset major vascular events within 90 days.

Other pre-specified efficacy endpoints are:

(1) Changes in total exercise duration (TED) during exercise tolerance testing (ETT). from baseline to Day 14 in 290 patients selected after first interim analysis;

(2) Changes in time to 1mm st-segment depression during exercise tolerance testing (ETT) from baseline to Day 14 in 290 patients selected after first interim analysis;

(3) Changes in the micro-RNA and mRNA profiles of 60 patients selected at the Chinese PLA General Hospital and Xuanwu Hospital Capital Medical University.

Safety:

(1) Overall mortality within 90 days;

(2) Incidence of severe haemorrhages within 90 days;

(3) Incidence of moderate haemorrhages within 90 days;

(4) Incidence of adverse and serious adverse events.

1. BACKGROUND INFORMATION

Stable ischemic heart disease is a leading cause of death worldwide, and nearly 58% of patients with coronary artery disease were suffering from chronic stable angina[1]. Current treatment options including including pharmacotherapy (as organic nitrates, β-blockers, calcium channel antagonists), revascularization, lifestyle management and several alternative procedures, aims to reduce the risk of mortality and morbid events and to reduce symptoms[2]. For patients, it is often the latter that is of greater concern[3]. Despite these multiple treatment options[2], a high proportion of patients with stable angina remains symptomatic and their quality of life is impaired[4,5]. Moreover, several observational studies have shown that angina symptoms such as physical limitation and angina frequency are predictive indicators of mortality and acute coronary syndrome (ACS) hospitalizations[6-8]. Therefore, we need more therapies for patients with chronic angina in order to prevent the episode of the angina for the improvement of their quality of life.

Danhong injection (DHI), which is extracted from Danshen (Radix Salviae miltiorrhizae) and Honghua (Flos carthami), is widely used to treat the coronary heart disease. DHI, the profile of which has been analyzed using HPLC, is approved as a Chinese medicinal product for the treatment of various ischemic diseases by the State Food and Drug Administration of China (SFDA) in 2002. Some clinical studies have demonstrated that Danhong injection might be an effective and safe treatment option for the management of coronary heart disease[9,10.] However, the methodological quality of most previous studies was assessed to be, in general, low[10]. No critically appraised evidence, such as a well-designed randomized controlled trial, is available to provide a high level of evidence to justify the clinical use and recommendation of DHI. In this study, we will conduct a randomized, multicentre, double-blind, placebo-controlled trial to investigate the efficacy and safety of DHI in patients with

chronic stable angina.

2. OBJECTIVES

The objective of this study is mainly to evaluate the effect of DHI in preventing the episode of chronic stable angina and improving the angina-specific quality of life, and the safety of DHI.

3. STUDY DESIGN

3.1 Overview

This study is a three-stage adaptive-design, randomized, multicentre, double-blind, placebo-controlled trial. The number of subjects is initially estimated to be 870 and randomly assigned to be 582 in DHI group and 288 in the control group. Both groups received optimal medical therapy as recent guideline advocated. Participants in the DHI group are treated by DHI (40 ml, qd) plus 0.9% normal saline (250 ml, ivgtt, qd), while participants in the control group are treated by DHI placebo (0.9% normal saline, 40ml, qd) plus 0.9% normal saline (250 ml, ivgtt, qd).

3.2 Sample size

According to the previous study[11], the proportion of patients who had clinically significant change defined as at least 20-point improvement in Seattle Angina Questionnaire angina frequency (SAQAF) score was 30%, after standard conventional therapy for a month. In this trial, we adopt an adaptive design by using the statistical sample size calculation software EAST5.2. It is hypothesized that an increase of at least 10% is of clinical significance for the DHI group; therefore, the number of subjects is initially estimated to be 726 (one-sided test, $\alpha=0.05$, $\beta=0.15$). To allow for a 20% dropout rate, a total of 870 patients will be recruited. As patients will be randomized into the DHI group or control group in

a ratio of 2:1, the number of participants in the DHI group is 582 and that in the control group is 288. According to the adaptive design, the sample size may be adjusted based on the results of two interim analyses, which will be carried out after one third (288) and two thirds (582) of patients have completed the trial, respectively.

3.3 Randomization

All eligible patients who consent to participation will be randomized into either the Danhong injection or the placebo group in a 2:1 ratio. Randomization will be conducted using a clinical information management system (Brightech, Somerset, USA). This system automatically randomizes patients and generates a randomization number with a message noting their assigned treatment. In addition, randomization will be stratified based on whether a patient received standard conventional therapy for more than 1 week prior to study initiation.

3.4 Blinding

Participants and research personnel will be blinded to Danhong injection therapy or placebo treatment group assignments until the study has concluded. Because the colour of Danhong injection and 0.9 % saline are different,the dropping bottles will be wrapped in sealed shaded bags, and brown infusion devices will be used for infusion. These procedures will be implemented by two professional nurses who will be required to sign a confidentiality agreement before study initiation and not to contact each other. One of the professional nurses will be in charge of preparing the drugs in a special transfusion room and sealing the infusion bottles with shaded brown bags. The other nurse will take the prepared drugs from the transfusion room to the infusion nurse and supervise the infusion process to ensure that the allocation of the drugs is blinded to the patients (the shaded brown bags will not be unwrapped during infusion and will be checked for integrity after infusion).

3.5 Interim analysis

Because this is an adaptive trial, two interim analyses are prospectively planned, and these will be performed in a blinded manner after one-third and two-thirds of the patients, respectively, have completed the trial. The statistical results of the interim analyses will be relayed to the data monitoring committee, who will decide on the re-estimation of sample size and determine whether any subsequent modifications must be made to the trial protocol. Because the two interim analyses may lead to an increased possibility of a type I error, we will apply the Lan-DeMets alpha spending function with an O'Brien-Fleming boundary to adjust the results.

4. SUBJECTS

4.1 Diagnostic criteria

4.1.1 Diagnostic criteria in Western Medicine

The diagnostic criteria for chronic stable angina are determined according to the Chinese Guidelines for the Diagnosis and Treatment of Chronic Stable Angina (2007)[2], the ACC/AHA Guideline Update for the Management of Patients With Chronic Stable Angina (2002)[12], and the European Society of Cardiology Guidelines for the Management of Stable Angina Pectoris[13].

Stable angina is characterized by transient episodes of chest pain precipitated by exercise or by other situations resulting in an increased myocardial oxygen demand. The pain usually disappears rapidly with rest or with sublingual nitroglycerin. The stable angina has 1 month's duration or more.

Patients must fulfill one of the following conditions:

(1) a history of myocardial infarction and ST-T changes;

(2) stenosis of more than 50 % in at least one major epicardial coronary artery, as shown by coronary angiography or computed tomography Angiography;

(3) coronary heart disease confirmed by radionuclide angiocardiography.

4.1.2 Grading of angina pectoris

According to the "Canadian Cardiovascular Society (CCS) grading of angina pectoris", the severity of angina can be graded as follows[14]:

Class I: Ordinary physical activity does not cause angina, such as walking and climbing stairs. Angina with strenuous or rapid or prolonged exertion at work or recreation.

Class II: Slight limitation of ordinary activity. Walking or climbing stairs rapidly, walking uphill, walking or stair climbing after meals, or in cold, in wind or under emotional stress, or only during the few hours after awakening. Walking more than two blocks on the level and climbing more than one flight of ordinary stairs at a normal pace and in normal conditions.

Class III: Marked limitation of ordinary physical activity. Walking one or two blocks on the level and climbing one flight of stairs in normal conditions and at normal pace.

Class IV: Inability to carry out any physical activity without discomfort; angina may be present at rest.

4.1.3. Diagnostic criteria in Traditional Chinese medicine

Traditional Chinese medicinediagnostic criteria are determined according to the Guidelines for Clinical Research of New Drugs of Traditional Chinese Medicine (2002)[15].

We will choose the patients with the a clinical diagnosis of "Xueyu Zheng" (blood stasis syndrome), which is defined as a score of at least 15 on the Chinese Medicine Symptom Scale of "Xueyu Zheng" for angina patients.

The Chinese Medicine Symptom Scale of "Xueyu Zheng" includes the following items:

(1) chest pain (0–10);

(2) chest distress (0–10);

(3) palpitation (0–5);

(4) purple or dark lips (0–5);

(5) purple or dark tongue (0–5);

(6) unsmooth pulse (0–5).

If the patient has a certain above symptom, corresponding 10 or 5 score will be in this symptom; otherwise, 0 for the symptom. The total score of at least 15 will be determined as “Xueyu Zheng”.

4.2 Inclusion Criteria

(1). Female or male inpatients.

(2). Age: 18–70 years.

(3). Patients with a clinical diagnosis of chronic stable angina.

(4). Patients with a clinical diagnosis of “Xueyu Zheng” (blood stasis syndrome).

(5). Patients with moderate angina pectoris, which is defined as Grade II or III on the Canadian Cardiovascular Society Angina Grading Scale.

(6). Patient is willing to voluntarily participate and to sign a written informed consent document.

4.3 Exclusion Criteria

(1). Women who are pregnant, lactating, having a positive pregnancy test, or having a menstrual period at baseline.

(2). Women with childbearing potential disagree with using contraception during the treatment period.

(3). Patients with severe complications that would complicate the condition, as assessed by the investigator, including liver or renal dysfunction, severe cardiopulmonary dysfunction, pulmonary hypertension, chronic obstructive pulmonary disease, a history of epilepsy or cerebral haemorrhage.

(4). Patients were angina-free during the run-in period without taking any drug.

(5). Patients experienced myocardial infarction or who were classified as Grade IV on the Canadian Cardiovascular Society Angina Grading Scale within the preceding 3 months.

(6). Patients with chest pain that is caused by any other disease (e.g., acute myocardial infarction, severe neurosis, menopausal syndrome or hyperthyroidism).

(7). Patients with a history of drug-induced bleeding or a history of bleeding caused by warfarin.

(8). Patients with a history of haematopoietic disorder.

(9). Patients have had surgery within the previous 4 weeks or who have a haemorrhagic tendency.

(10). Patients who are participating in other trials or who have participated in other trials within the past 3 months.

(11). Patients with a history of allergy or with a known or suspected allergy to the study drug.

(12). Patients with a known or suspected history of alcohol or drug abuse within the past 2 years.

(13). Patients with a mental disorder.

(14). Patients who are unable to participate in the study, as judged by the investigator.

(15). Family members or relatives of the study centre staff.

4.4 Withdrawal/dropout criteria

(1) Those who did not fulfill the eligibility criteria but were included by mistake;

(2) Those who fulfilled the eligibility criteria but did not take any study drug after randomization;

(3) Those with poor compliance that might interfere with the efficacy and safety evaluation;

(4) Those who experienced serious AEs, complications or special physiological changes, making it inappropriate to continue with the trial;

(5) Individual cases that were unblinded prematurely;

(6) Those who discontinued the trial voluntarily;

(7) Those who used any disallowed concomitant medication, especially those that had an obvious effect on the study drug and might interfere with the efficacy and safety evaluation;

(8) Those who discontinued or lost to follow-up for any other reasons or died during the trial;

(9) Those with incomplete data that might interfere with the efficacy and safety evaluation.

For all withdrawal/dropout cases, the potential reasons must be recorded. Patients who experienced any AE during the trial must be included in the statistical analysis of adverse drug reactions. Those who have taken the study drug for at least 1 week should be included in the statistical analysis of drug efficacy.

4.5 Criteria for study termination

Circumstances that may warrant termination include, but are not limited to:

(1) Identification of unexpected, significant, or unacceptable risk to subjects;

(2) Poor efficacy or no efficacy of the study drug;

(3) Major mistakes in the trial protocol;

(4) Insufficient adherence to protocol requirements;

(5) Upon sponsor's request (e.g. economic or administrative reasons).

4.6 Criteria for subject discontinuation

(1) Any allergic reaction or AE occurs such that continued participation in the study would not be in the best interest of the subject at the discretion of the treating physician;

(2) Worsening of clinical conditions occurs such that continued participation in the study would not be in the best interest of the subject at the discretion of the treating physician; considered as ineffective cases;

(3) Subjects are free to withdraw from participation in the study at any time upon request.

In any case, every effort must be made to determine why patients discontinued the study treatment prematurely, which may include but are not limited to poor confidence in efficacy, occurrence of AEs, SAEs, or other serious complications, worsening of symptoms such that emergent intervention is required. As for those who withdrew from participation in the study or lost to follow-up, it is essential to investigate the potential reasons via telephone. Investigators should record in detail when and how the last dose was administered, the efficacy and safety parameters at and after discontinuation, the relationship between discontinuation or withdrawal and the study drug, and the potential impact of discontinuation cases on the final conclusion of the study. Moreover, case report form (CRF) of withdrawal/dropout cases should also be completed and their original data should also be documented and stored in a secure manner.

5 INTERVENTION

5.1 Optimal medical therapy

Optimal medical therapy will be provided to all of the included participants throughout the trial, in strict accordance with the Chinese Guidelines for the Diagnosis and Treatment of Chronic Stable Angina (2007) [2].

Optimal medical therapy includes:

(1) Antiplatelet agents: aspirin (75-100 mg, once per day) or clopidogrel (if the patient is intolerant to aspirin). Patients with a history of percutaneous coronary intervention will be prescribed both of these agents.

(2) Lipid-lowering agents (statins): atorvastatin (10-20 mg, once per day) or simvastatin (20-40 mg, once per day).

(3) Anti-angina agents: β-blockers (metoprolol 50-200 mg, once per day, or analogous agents);

long-acting nitrates (isosorbide mononitrate 40-60 mg, once per day); or calcium channel blockers (amlodipine 5-20 mg, once per day).

(4) Patients with diabetes and hypertension will be advised to take angiotensin-converting enzyme inhibitors or angiotensin-receptor blockers (e.g., lisinopril 10-20 mg, once per day, or losartan 50 mg, once per day) as a secondary preventive measure for chronic stable angina.

All of these basic treatments will be recorded in detail in the patients' medical records as well as in their e-CRFs.

Participants who received optimal medical therapy for more than 1 week prior to study initiation will be randomized directly into one of the groups; otherwise, a run-in period of 1 week of optimal medical therapy will be performed before randomization.

5.2 Treatment regimens

DHI group: Participants will be treated using Danhong injection (40 ml per day) plus 0.9 % normal saline (250 ml intravenously per day).

Control group: Participants will be treated using placebo (0.9 % normal saline, 40 ml per day) plus 0.9 % normal saline (250 ml intravenously per day).

5.3 Duration and follow-up

All of the included patients will undergo a 2-week treatment regimen and a 76-day follow-up period.

5.4 Concomitant medications

(1) Sublingual nitroglycerin tablet (0.5 mg per tablet, provided by Beijing Yimin Pharmaceutical Co., Ltd.)is permitted in case of an angina attack, and this dose can be repeated approximately every 5 minutes until the angina is relieved. However, if the angina persists after three doses, the patient should

be transported to hospital immediately for further medical treatment. Patients will be asked to record the details of administration times, the number of doses and the dosages of nitroglycerin in a patient diary, which will be collected by the investigators at a subsequent study visit.

(2) Other WM medications targeting the treatment of angina or CHD will be disallowed during the trial.

(3) Participants are not allowed to take any other Chinese herbal medicines or Chinese patent medicines that have the effect of promoting blood circulation or removing blood stasis throughout the study period.

(4) If other medication or therapy is required to treat the concomitant diseases, then the name of the drug or therapy, actual dosage, dosing frequency and start/stop time should be well-documented.

6 OUTCOMES MEASUREMENTS

6.1 Primary outcome

Angina-specific health status will be assessed at baseline, Day 30, 60, and 90. Each assessment will be performed with the use of the Seattle Angina Questionnaire (SAQ), a 19-item self-administered questionnaire that measures 5 domains of CAD-related health status: physical limitation(PL), angina frequency(AF), treatment satisfaction(TS), and disease perception/quality of life(DP). The total score ranges from 0 to 100; and higher scores indicate better health status.[11]

The primary outcome is the proportion of patients who have a clinically significant change in Seattle Angina Questionnaire angina frequency (SAQAF) score at Day 30.

A clinically significant change in each scale of SAQ was defined as a difference of 8 points or more on the physical-limitation scale, 25 or more on the angina-stability scale, 20 or more on the angina-frequency scale, 12 or more on the treatment-satisfaction scale, and 16 or more on the

quality-of-life scale, respectively.[11]

6.2 Secondary Outcomes

6.2.1 The total score of symptoms in a questionnaire related to traditional Chinese medicine .

The efficacy evaluation scale of Chinese Medicine Symptom in “Xueyu Zheng” (Table 1) will be used to assess the condition of patients with Xueyu Zheng at each visit (Day 0, Day 7, Day 14, Day 30, Day 60, Day 90).

Table 1 The efficacy evaluation scale of Chinese Medicine Symptom in “Xueyu Zheng”

Symptom	Visual Analogue Scale*	Score
Chest pain	0-----------------------------------10	
Chest distress	0-----------------------------------10	
Palpitation	0-----------------------------------5	
Purple or dark lips	0-----------------------------------5	
Purple or dark tongue	0-----------------------------------5	
Unsmooth pulse	No--0 Yes---5	

* According to the severity of the symptoms, the more severe the symptom is, the higher the score be determined. If the symptom is disappeared, the score will be recorded as 0.

The mean total score of symptoms in a questionnaire of *Xueyu Zheng* will be analyzed and compared between two groups at each visit. Moreover, the proportions of patients with syndrome improvement in *Xueyue Zheng* between the two groups will be recorded at each visit. Significant syndrome improvement is defined as at least 30% reduction in the *Xueyue Zheng* score[15].

6.2.2 The proportion of patients who experience clinically significant changes in the other four Seattle Angina Questionnaire domains (physical limitation, angina stability, treatment satisfaction, and disease perception/quality of life)[11].

At Day 30, 60, 90, investigators will observe the proportions of patients in each treatment group who were angina-free. Angina-free is defined as a score of 100 in the angina frequency score on the SAQ.

6.2.3 The frequency of angina attacks.

The patients will be asked to record their angina attacks in the diary. At each visit (Day 7, 14, 30, 60, 90), the investigator will collect the diary and record it onto the electronic case report forms (e-CRFs).

6.2.4 Angina grade, according to the Canadian Cardiovascular Society (CCS) Angina Grading Scale.

At each visit, the angina grade of each patient will be evaluated according to the Canadian Cardiovascular Society (CCS) Angina Grading Scale. The proportion of patients who have the improvement of the Canadian Cardiovascular Society (CCS) Angina Grading Scale will be compared between two groups at each visit (Day 7, 14, 30, 60, 90), which is defined that the CCS grade change to Grade I or decrease 1 grade.

6.2.5 Consumption of short-acting nitrates.

The patients will be asked to record their usage of short-acting nitrates in the diary. At each visit (Day 0, 7, 14, 30, 60, 90), the investigator will collect the diary and record it onto the e-CRF.

6.2.6 Changes in electrocardiogram results.

The 12-lead electrocardiogram (ECG) will be tested at each visit (Day 0, 7, 14, 30, 60, 90). The result will be evaluated by two independent ECG doctors. The proportion of patients with normal ECG recordings will be compared between the two groups at each visit. The "normal ECG" in our trial is defined as including (1) normal sinus rhythm (each P wave is followed by a QRS and P wave rate 60 - 100 bpm with <10% variation); (2) normal P waves (height < 2.5 mm in lead II and width < 0.11 s in lead II); (3) normal PR interval (0.12 to 0.20 s); (4) normal QRS complex (< 0.12 s duration, no pathological Q waves and no evidence of left or right ventricular hypertrophy); (5) normal QT interval (0.42 s); (6) normal ST segment without any elevation or depression; (7) normal T wave; and (8) normal U wave.

6.2.7 Changes in serum lipid levels at Day 14.

6.2.8 Changes in high-sensitivity C-reactive protein levels at Day 14.

6.2.9 Changes in the platelet aggregation rate at Day 14.

6.3 Other Pre-specified Efficacy Outcomes

(1) Changes in total exercise duration (TED) during exercise tolerance testing (ETT) from baseline to Day 14 in 290 patients selected after first interim analysis;

(2) Changes in time to 1mm st-segment depression during exercise tolerance testing (ETT) from baseline to Day 14 in 290 patients selected after first interim analysis;

(3) Changes in the micro-RNA and mRNA profiles of 60 patients selected at the Chinese PLA General Hospital and Xuanwu Hospital Capital Medical University.

6.4 Safety Outcomes

6.4.1 Incidence of new-onset major vascular events within 90 days.

Major vascular events include death induced by cardiac or cerebral vascular disorders , myocardial infarction, stroke, transient ischemic attack (TIA), coronary intervention (including stent thrombosis), peripheral vascular intervention, hospitalization for unstable angina, and acute heart failure (HF) events.

6.4.2 Overall mortality within 90 days.

6.4.3 Incidence of severe haemorrhages within 90 days.

Severe haemorrhages includes fatal bleeding, primary intracranial hemorrhage, post-traumatic symptomatic intracranial hemorrhage, or bleeding requiring transfusion, infusion, use of vasoconstrictor drugs and surgical intervention.

6.4.4 Incidence of moderate haemorrhages within 90 days.

Moderate haemorrhages includes bleeding requiring infusion but do not reach severe bleeding, absolute reduction in hemoglobin/hematocrit, severe disability induced by bleeding, intraocular hemorrhage with severe visual field defects, etc.

6.4.5 Incidence of adverse and serious adverse events (Section 7).

6.5 Study Schema

Period	Run-in period	Treatment period			Follow-up period		
Day	-7	0	7	14	30	60	90
Informed consent	×	×					
Inclusion/exclusion criteria	×	×					
Medical history	×	×					
Medical examination	×						
Combined disease treatment	×	×	×	×	×	×	×
Outcomes							
SAQ		×			×	×	×
Symptoms questionnaire of TCM		×	×	×	×	×	×
Frequency of anginal attack per week	×	×	×	×	×	×	×
CCS grade		×	×	×	×	×	×
Consumption of short-acting nitrates	×	×	×	×	×	×	×
ECG		×	×	×	×	×	×
Serum lipids		×		×			
Hs-CRP		×		×			
Platelet aggregation rate		×		×			
New-onset major vascular events							×
Overall mortality							×
Incidence of severe hemorrhages							×
Incidence of moderate hemorrhages							×
AEs and SAEs			×	×	×	×	×
ETT (if necessary after 1st interim analysis)		×		×			
Profiles of micro-RNA in 60 patients in certain centers		×		×			×
Profiles of mRNA in 60 patients in certain centers		×		×			×

7 ADVERSE EVENTS

7.1 Definition

Adverse event (AE): An AE is any untoward medical occurrence in a patient or clinical investigation subject administered a pharmaceutical product and that does not necessarily have a causal

relationship with this treatment. An AE can therefore be any unfavorable and unintended sign (including an abnormal laboratory finding), symptom, or disease temporally associated with the use of a medicinal (investigational) product, whether or not related to the medicinal (investigational) product. Pre-existing conditions which worsen during a study are also to be reported as AEs.

Serious Adverse Event (SAE): A SAE is any experience that suggests a significant hazard, contraindication, side effect or precaution. It is any AE that at any dose fulfils at least one of the following criteria:

– is fatal;

– is life-threatening;

– required in-patient hospitalization or prolongation of existing hospitalization;

– results in persistent or significant disability/incapacity;

– is a congenital anomaly/birth defect;

– is medically significant or requires intervention to prevent one or other of the outcomes listed above.

Adverse drug reaction (ADR): An ADR is any unintended harm or injury associated with the use of given medications at a normal dosage during normal use. In clinical trials of any new drug or any new usage of a certain drug, any unintended harm or injury that has a causal relationship to the use of the drug should also be considered as an ADR.

7.2 Recording

For all AEs encountered during the clinical study, the following must be assessed and recorded on the adverse events form of the e-CRF: intensity, relationship to study drug, action taken regarding study drug, and outcome to date. All AEs, especially those for which the relationship to test drug is not

“unrelated”, should be followed up until they have returned to baseline status or stabilized. The follow-up procedures could be performed in clinic visits or during hospitalization, or via home visit, telephone or mail, depending on the severity of AEs.

7.3 Severity of an AE

Mild: events require minimal or no treatment and do not interfere with the subject’s daily activities.

Moderate: events result in a low level of inconvenience or concern with the therapeutic measures. Moderate events may cause some interference with functioning.

Severe: events interrupt a subject’s usual daily activity and may require systemic drug therapy or other treatment. Severe events are usually incapacitating.

7.4 Evaluation the correlation between AEs and study drugs

Based on the current 6-level evaluation standard of the China National Center for Adverse Drug Reaction Monitoring, according to the answers to 5-iterm questionnaire, the correlation between AEs and study drugs can be categorized as to definitely related, probably related, possibly related, unlikely related, to be evaluated, and unable to evaluate (Table 2) . The adverse drug reaction was determined as "definitely related", "probably related", " possibly related".

Table 2 The assessment of the causality between adverse events and observed drug

<table>
<tr><th rowspan="2">Item*</th><th colspan="6">Index</th></tr>
<tr><th>1= definitely related</th><th>2= probably related</th><th>3= possibly related</th><th>4= unlikely related</th><th>5= to be evaluated</th><th>6= unable to evaluate</th></tr>
<tr><td>A reasonable timing relationship[1]</td><td>+</td><td>+</td><td>+</td><td>-</td><td rowspan="5">Additional detailed information to be evaluated</td><td rowspan="5">The necessary information for the evaluation is not available</td></tr>
<tr><td>Belonging to the Known ADR of the drug [2]</td><td>+</td><td>+</td><td>±</td><td>-</td></tr>
<tr><td>Relief after stopping the drug[3]</td><td>+</td><td>+</td><td>± ?</td><td>± ?</td></tr>
<tr><td>Recurrent AE after re-administration[4]</td><td>+</td><td>?</td><td>?</td><td>?</td></tr>
<tr><td>Suspected adverse reaction could be explained with other certain reasons[5]</td><td>-</td><td>-</td><td>± ?</td><td>± ?</td></tr>
</table>

*The 5-iterm questionnaire to assess the causality between adverse events and observed drug is listed in the items 1 to 5, detailed as following:

1. Is there a reasonable relationship between the time of starting the drug and the time of occurrence

of the suspected adverse reaction?

2. Is the suspected adverse drug reaction consistent with the types of adverse reaction known to the drug?

3.Could the suspected adverse reaction be alleviated or disappeared after stopping the drug or reducing the dosage?

4. Dose the same adverse event occur again after given the same drug again?

5. Could the suspected adverse reaction be explained by the patient's pathological condition, combined medication, combined therapy, and used therapy etc?

7.5 Handling of SAEs

All SAEs should be managed properly and timely, and recorded on the appropriate SAE eCRF. Any AE considered serious by the investigator or which meets the aforementioned criteria must be submitted on a signed and dated SAE form to the sponsor, ethics review committee and relevant authorities within 24 hours of site awareness. And the sponsor should also inform all study sites of such SAEs in a timely manner to ensure subjects' safety in accordance with applicable laws and regulations.

Fatal or life-threatening events considered to be caused by the study drugs must be reported immediately to the sponsor and relevant authorities via telephone, fax, EMS or E-mail.

7.6 Opening and handling of emergency envelops

The emergency envelope provided along with each test drug in its package should only be opened in case of a serious complication or a serious adverse event, which is called emergency unblinding. Once unblinded, the patient should be withdrawn from the study and regarded as a dropout case. Information about when, where and why the emergency unblinding occurs as well as each personnel's signature should also be documented.

7.7 Follow-up of unresolved AEs

All AEs should be followed up until they have been resolved, stabilized or returned to baseline status.

8. DATA COLLECTION AND MANAGEMENT

8.1 Establishment of Three Committees

To ensure the effectiveness and integrity of the trial design, three committees have been established by the multicentre trial coordination group: the clinical trial guidance committee (Executive committee), the data monitoring committee (DMC) and the outcome evaluation committee. The first committee is responsible for the study design and the implementation process. The data monitoring committee will supervise the integrity and accuracy of data collection to control its quality. The outcome evaluation committee will evaluate key outcomes (including outcome measurements and adverse events) based on their clinical expertise.

8.2 Data Management

(1) All data will be recorded by trained clinical investigators using standardized e-CRFs and Brightech clinical information management systems (Brightech, Somerset, NJ, USA).

(2) The accuracy and reliability of the data will be ensured by the study monitor, who will verify and cross-check the e-CRFs against the investigator's records (source document verification), using the maintenance of a drug dispensing log by the investigator.

(3) Front-end checks in the e-CRFs and back-end checks in the clinical information management system will be used as a validation check of the data.

(4) If there are any discrepancies in the e-CRFs, the results will be sent to the investigator for resolution.

(5)The results of the analysis must not be released with individual identification of the subjects until the database is closed.

8.3 Data Report

Data report contains the aspects as followed: (1) members of the project; (2) disagreement from

the primary data management plan; (3) actual finish time of every project; (4) problems and the solution during the data management (if have any); (5) reconstruction of the database (if have any); (6) distribution of the participants; (7) participants who disobey the trial protocol; (7) classifying plan of the statistical analysis population. Data report will be performed monthly and then send to DMC since the first entry of the e-CRF.

8.4 Data Auditing and Blinding Review

When the data checking is finished, a data auditing and blinding review meeting will be hold. On the meeting, the data administrators, statisticians, researchers, clinical inspectors, and other related members would have a discussion on the following items according to the data management report and the data lists:

•Distribution of the participants;

•Protocol disobeying or not;

•Possible outlier;

•Baseline data;

•Outcomes;

•Statistical analysis plan.

Participants will be classified to their suitable statistical analysis sets according to the definition in the protocol. No patient can be excluded from the analysis, unless getting the permission of the meeting participants. All the meeting participants should sign the data locking consent, and the data auditing resolution.

8.5 Database Locking

The database will be locked if it fulfills all the aspects as followed:

•All the queries have been solved, and the database has been updated;

•No query has been found through the data inspection;

•The medical coding has been completed;

•The plan of the participants' classification has been approved;

•The final draft of the SAP has been made, and approved by the project leader.

The statisticians and the data administrators will sign the data locking form, and then the database will be locked. The locked database will be sent to the statisticians for further statistical analysis through the data format of SAS.

9. STATISTICAL CONSIDERATIONS

9.1 Study Populations

All patients with randomization will be included in the analysis set regardless of whether they receive any treatment. Most outcome measurements (except ~~*the ETT,*~~ micro-RNA and mRNA profiles) will be analyzed using full analysis sets and per-protocol sets according to intention-to-treat analysis. Safety analysis will be performed in a safety set, which is defined as a subset of subjects who were randomized and who received at least one treatment.

9.2 Statistical Analyses

The primary study hypothesis is that an initial usage of DHI added to optimal medical therapy could bring more clinical benefit for the patients with symptomatic stable angina than an initial strategy of optimal medical therapy alone. The primary analysis will be intention-to-treat with multiple imputations.The chi-square test will be used to compare the proportion of patients who have a clinically significant change in SAQ angina frequency at Day 30 from baseline (a post hoc analysis) between the two groups. Pre-specified subgroup analysis will also be performed according to the Statistical Analysis

Plan (SAP).

The secondary efficacy and safety outcomes will be analyzed using the two sample t-tests, Wilcoxon rank-sum test, Chi-square test, Poisson regression or Fisher's exact test according to the Statistical Analysis Plan (SAP).

All reported P values were two-sided; and a P value less than 0.05 was considered to be statistically significant except that from the primary outcome analyses, in which Bonferroni's multiple comparison test was applied twice and P <0.016 was considered significant. All calculations were performed using SAS 9.4 (SAS Institute Inc, USA).

9.3 Statistical Analysis Plan (SAP)

Prior to database lock and before code breaking, a final version of the SAP shall be issued and approved by the study statistician, and the principal investigator. The SAP will define all "pre-specified, planned analyses" and provide the general specifications for the analysis of the data to be collected and presented in clinical information management systems.

10 ETHICAL ASPECTS

The investigator will ensure that this study is conducted in full conformance with the principles of the "Declaration of Helsinki" and applicable laws and regulations on the conduct of clinical trials.

This trial firstly will be approved by center institutional review board (IRB) in Chinese PLA General Hospital. For the other sites, if need, the trail also should be approved by the local IRB at the certain enrolling sites. Only when the trial protocol is approved by the IRB, the enrollment of participant will begin.

Informed consent is a process that is initiated prior to the individual's agreeing to participate in the study and continues throughout the individual's study participation. Extensive discussion of risks and

possible benefits of this treatment will be provided to the subjects and their families. Consent forms describing in detail the study interventions/products, study procedures, and risks are given to the subject and written documentation of informed consent is required prior to starting intervention/administering study product. It must also be explained to the patients that they are completely free to refuse to enter the study or to withdraw from it at any time for any reason. A copy of the informed consent document will be given to the subjects for their records.

REFERENCES

1. Lloyd-Jones, D. M. et al. Defining and setting national goals for cardio vascular health promotion and disease reduction: The American Heart Association's strategic impact goal through 2020 and beyond. Circulation 2010,121, 586-613

2. Chinese Society of Cardiology, Chinese Medical Association; Editorial Board, Chinese Journal of Cardiology. [Guideline for diagnosis and treatment of patients with chronic stable angina (no abstract)]. Zhonghua Xin Xue Guan Bing Za Zhi. 2007;35(3):195-206.

3. Gibbons, R. J. et al. Guidelines for the diagnosis and treatment of chronic stable angina. J Am Coll Cardiol 1999,133, 2092-2197

4. Wiest, F. C. et al. Suboptimal pharmacotherapeutic management of chronic stable angina in the primary care setting. Am J Med 2004,117, 234-241.

5. Kirwan, B. A., Lubsen, J. & Poole-Wilson, P. A. Treatment of angina pectoris: associations with symptom severity. Int J Cardiol 2005,98, 299-306

6. Spertus, J. A., Jones, P., McDonell, M., Fan, V. & Fihn, S. D. Health status predicts long-term outcome in outpatients with coronary disease. Circulation 2002,106, 43-49

7. Mozaffarian, D., Bryson, C. L., Spertus, J. A., McDonell, M. B. & Fihn, S. D. Anginal symptoms consistently predict total mortality among outpatients with coronary artery disease. Am Heart J 2003,146, 1015-1022.

8. Berecki-Gisolf, J., Humphreyes-Reid, L., Wilson, A. & Dobson, A. Angina symptoms are associated wityhh mortality in older women with ischemic heart disease. Circulation 2009,120, 2330-2336

9. Du J, Yang W, Yi D, Xie Y, Yang W, Zhuang Y, et al. Analysis of using Danhong injection to treatment coronary heart disease patients medicines based on real world HIS database. Zhongguo Zhong Yao Za Zhi. 2011;36:2821-2824.

10. Peng LH, Yu Z, Sheng CL. Dan Hong Injection for Angina Pectoris: A Systematic Review. Chin J Evid-based Med 2011, 11(1): 57-63

11. Weintraub WS, Spertus JA, Kolm P, Maron DJ, Zhang Z, Jurkovitz C, et al. Effect of PCI on quality of life in patients with stable coronary disease. N Engl J Med. 2008;359:677-87.

12. Gibbons RJ, Abrams J, Chatterjee K, Daley J, Deedwania PC, Douglas JS, et al. ACC/AHA 2002 guideline update for the management of patients with chronic stable angina-—summary article: a report of the American College of Cardiology/American Heart Association Task Force on Practice Guidelines (Committee on the Management of Patients With Chronic Stable Angina). Circulation. 2003;107:149-58.

13.Fox K, Garcia MA, Ardissino D, Buszman P, Camici PG, Crea F, et al.Guidelines on the management of stable angina pectoris: executive summary: the Task Force on the Management of Stable Angina Pectoris of the European Society of Cardiology. Eur Heart J. 2006;27:1341-81.

14. Campeau L. Grading of angina pectoris. Circulation 1976;54:522–3.

15. Zheng X. Guidelines of Clinical Research of New Drugs of Traditional Chinese Medicine. Beijing: China Medical Science Press; 2002.

Protocol (Final)

The Final Version: October 15, 2015

Danhong Injection for the treatment of chronic stable angina (Phase IV): an adaptive-design, randomized, multi-center, double-blind, placebo-controlled clinical trial

Sponsored by: China National Key Technologies R&D Program on "New Drug R&D" (2011ZX09304-07)

EXECUTIVE COMMITTEE CHAIR

Prof. Yong-Yan Wang, Academician of Chinese Academy of Engineering

Institute of Basic Research in Clinical Medicine, China Academy of Chinese Medical Sciences

No.16 Nanxiaojie, Dongzhimennei, Beijing, 100700, China.

Tel: 86-10-84046033

E-mail: wangyongyan2010@sina.cn

PRINCIPLE INVESTIGATORS

Prof. Zhong Wang, Ph.D (for grant)

Institute of Basic Research in Clinical Medicine, China Academy of Chinese Medical Sciences

No.16 Nanxiaojie, Dongzhimennei, Beijing, 100700, China.

Tel:86-10-64093305

Email: zhonw@vip.sina.com

Prof. Yun-Dai Chen, M.D. (for trial)

Department of Cardiology, Chinese PLA General Hospital

No. 28 Fuxing Road, Beijing, 100853, China.

E-mail: cyundai@medmail.com.cn

PARTICIPATING SITES AND INVESTIGATOR

7. Chinese PLA General Hospital

Prof. Yun-Dai Chen, M.D. (for trial)

Site Principal Investigator, Director of Department of Cardiology

Chinese PLA General Hospital

No. 28 Fuxing Road, Beijing, 100853, China

F-mail: cyundai@medmail.com.cn

Prof. Wei Dong

Co-investigator, Department of Cardiology

Chinese PLA General Hospital

No. 28 Fuxing Road, Beijing, 100853, China

E-mail: 301dongw@sina.com

Dan-Dan Li

Co-investigator, Department of Cardiology

Chinese PLA General Hospital

No. 28 Fuxing Road, Beijing, 100853, China

E-mail: Ametuofo980869@163.com

Yu-Qi Liu

Co-investigator, Department of Cardiology

Chinese PLA General Hospital

No. 28 Fuxing Road, Beijing, 100853, China

E-mail: 7399253@qq.com

8. Dongfang Hospital Affiliated to Beijing University of Chinese Medicine

Prof. Yang Wu, M.D.

Site Principal Investigator, Director of Department of Cardiology

Dongfang Hospital Affiliated to Beijing University of Chinese Medicine

No. 6, Zone 1 Fangxinyuan, Fangzhuang, Beijing, 100078, China

E-mail:drwuyang@163.com

Da-Xuan Tang, M.D.

Co-investigator, Department of Cardiology

Dongfang Hospital Affiliated to Beijing University of Chinese Medicine

No. 6, Zone 1 Fangxinyuan, Fangzhuang, Beijing, 100078, China

E-mail: tang_dax@163.com

9. Peking University Third Hospital

Prof. Fu-Chun Zhang

Site Principal Investigator, Director of Department of Cardiology

Peking University Third Hospital

No.49, Huayuan North Road, Beijing, 100191, China

E-mail: Zhang.fuchun @medmail.com.cn

10. Xuan Wu Hospital, Capital Medical University

Prof. Qi Hua, M.D.

Site Principal Investigator, Director of Department of Cardiology

Xuan Wu Hospital, Capital Medical University

No.45, Changchun Street, Beijing, 100053, China

E-mail: huaqi5371@medmail.com.cn

11. **Guang'anmen Hospital, China Academy of Chinese Medical Sciences**

Prof. Jun Li, M.D.

Site Principal Investigator, Director of Department of Cardiology

Guang'anmen Hospital, China Academy of Chinese Medical Sciences

No.5, Beixiange, Beijing, 100053, China

Email: 13051458913@163.com

12. **The First Affiliated Hospital of Henan University of T.C.M.**

Prof. Ting-Hai Du

Site Principal Investigator, Director of Department of Cardiology

The First Affiliated Hospital of Henan University of T.C.M.

No. 19, Renmin Road, Zhengzhou, 450000, Henan, China

Email: dth2010@163.com

13. ***Renmin Hospital of Wuhan University***

Prof. Xue-Jun Jiang, M.D.

Site Principal Investigator, Director of Department of Cardiology

Renmin Hospital of Wuhan University

No.238, Jiefang Road, Wuchang, Wuhan, 430060, Hubei, China

Email: xjjiang@whu.edu.cn

14. ***Zhongnan Hospital of Wuhan University***

Prof. Yang-Gan Wang, M.D.

Site Principal Investigator, Director of Department of Cardiology

No.169, Donghu Road, Wuchang, Wuhan, 430030, Hubei, China

Email: ygwang2013@163.com

15. ***Affiliated Zhongshan Hospital of Dalian University***

Prof. Qin Yu, M.D.

Site Principal Investigator, Director of Department of Cardiology

No.6, Jiefang Street, Dalian, 116001, Liaoning, China

Email: yuqin060621@126.com

16. ***The Second Affiliated Hospital to Hunan University of Chinese Medicine***

Prof. Yi-Lin Mao, M.D.

Site Principal Investigator, Director of Department of Cardiology

No.233, Cai'e North Road, Changsha, 410005, Hunan, China

Email: maoyilin8518@126.com

17. ***The Affiliated Hospital to Changchun University of Chinese Medicine***

Prof. Yue Deng, M.D.

Site Principal Investigator, Director of Department of Cardiology

The Affiliated Hospital to Changchun University of Chinese Medicine

No.147, Gongnongda Road, Changchun, 130021, Jilin, China

Email: dyue7138@sina.com

18. The First Affiliated Hospital to Shanxi Medical University

Prof. Qing-Hua Han, M.D.

Site Principal Investigator, Director of Department of Cardiology

The First Affiliated Hospital to Shanxi Medical University

No.85, Jiefang South Road, Taiyuan, 030001, Shanxi, China

Email: syhqh@sohu.com

19. Affiliated Hospital of Shanxi University of Chinese Medicine

Prof. Ming-Jun Zhao

Site Principal Investigator, Director of Department of Cardiology

Affiliated Hospital of Shanxi University of Chinese Medicine

No.2, Weiyang West Road, Xianyang, 712000, Shanxi, China

Email: zmj7125@163.com

20. Changsha Central Hospital

Prof. Hai-Ying Dai

Site Principal Investigator, Director of Department of Cardiology

Changsha Central Hospital

No.161, Shaoshan South Road, Changsha, 410004, Hunan, China

Email: daihaiying2009@163.com

21. Hubei Provincial Hospital of T.C.M.,

Prof. You-Zhi Hu

Site Principal Investigator, Director of Department of Cardiology

Hubei Provincial Hospital of T.C.M.

No.4, Huayuanshan, Wuhan, 430061, Hubei, China

Email: hyz2003@medmail.com.cn

22. Shanxi Provincial People's Hospital

Prof. Kun Xing

Site Principal Investigator, Director of Department of Cardiology

Shanxi Provincial People's Hospital

No. 256, Youyi West Road, Xi'an, 710003, Shanxi, China

Email: 15891761516@163.com

23. Shanxi Province Hosptial of T.C.M.

Prof. Chao-Feng Liu

Site Principal Investigator, Director of Department of Cardiology

Shanxi Province Hosptial of T.C.M.

No. 2, Xihuamen, Xi'an, 710068, Shanxi, China.

Email: liu.reld@aliyun.com

24. Xi'an City Hospital of T.C.M.

Prof. Feng-Chun Li

Site Principal Investigator, Director of Department of Cardiology

Xi'an City Hospital of T.C.M.

No.69, Fengcheng Eight Road, Xi'an, 710001, Shanxi, China

Email: lifengchunyisheng@163.com

25. Shanghai Tongji Hospital,

Prof. Jin-Fa Jiang

Site Principal Investigator, Director of Department of Cardiology

Shanghai Tongji Hospital

No.398, Xinchun Road, Putuo District, Shanghai, 200065, China

Email: jiangjinfa@sohu.com

26. Shanghai Municipal Hospital of Traditional Chinese Medicine

Prof. Yao-Rong Dong

Site Principal Investigator, Director of Department of Cardiology

Shanghai Municipal Hospital of Traditional Chinese Medicine

No.274, Middle Zhijiang Road, Shanghai, 200071, China

Email: dyr19600618@sina.com

27. The First Hospital of Changsha

Prof. Guang Fu

Site Principal Investigator, Director of Department of Cardiology

The First Hospital of Changsha

No.311, Yingpan Road, Changsha, 410005, Hunan, China

Email: fuguangyisheng@163.com

28. Xin Hua Hospital of Zhejiang Province

Prof. Shu-Wei Huang

Site Principal Investigator, Director of Department of Cardiology

Xin Hua Hospital of Zhejiang Province

No.318, Chaowang Road, Hangzhou, 310005, Zhejiang, China

Email: hsw1104@163.com

29. Second Affiliated Hospital of Tianjin University of Traditional Chinese Medicine

Prof. Lan-Jun Sun

Site Principal Investigator, Director of Department of Cardiology

Second Affiliated Hospital of Tianjin University of Traditional Chinese Medicine

No.69, Zengchan Dao, Tianjin, 300150, China

Email: sunlanjun2015@163.com

30. Shanghai Tenth People's Hospital

Prof. Yun Xia

Site Principal Investigator, Director of Department of Cardiology

Shanghai Tenth People's Hospital

No.301, Middle Yanchang Road, Shanghai, 200072, China

Email: drxiayun@hotmail.com

31. Chongqing Traditional Chinese Medicine Hospital

Prof. Fan Liu

Site Principal Investigator, Director of Department of Cardiology

Chongqing Traditional Chinese Medicine Hospital

No.40, Daomenkou, Yuzhong District, Chongqing, 400011, China

Email: daliufan@sina.com

32. The Third People's Hospital of Chongqing

Prof. Jian-Cong Tan, M.D.

Site Principal Investigator, Director of Department of Cardiology

The Third People's Hospital of Chongqing

No.104, Pibashan Street, Yuzhong District, Chongqing, 400014, China

Email: tanjianchong@yahoo.com.cn

33. The First Affiliated Hospital of Heilongjiang University of Traditional Chinese Medicine

Prof. Ya-Bin Zhou, M.D.

Site Principal Investigator, Director of Department of Cardiology

The First Affiliated Hospital of Heilongjiang University of Traditional Chinese Medicine

No.28, Heping Road, Xiangfang District, Ha'erbin,150040,Heilongjiang, China

Email: zhouyabin_01@sina.com

34. The Second Affiliated Hospital of Heilongjiang University of Traditional Chinese Medicine

Prof. Guo-Qing Ma, M.D.

Site Principal Investigator, Director of Department of Cardiology

The Second Affiliated Hospital of Heilongjiang University of Traditional Chinese Medicine

No.411, Guogeli Street, Nangang District, Ha'erbin, 150009, Heilongjiang, China

Email: 13351685189@163.com

29. Jilin Province People's Hospital,

Prof. He-Ping Liu, M.D.

Site Principal Investigator, Director of Department of Cardiology

Jilin Province People's Hospital

No.1183, Gongnong Street,Chaoyang District, Changchun, 130021, Jilin, China

Email: heping-liu@sohu.com

30. Chengdu Sencond People's Hospital

Prof. Jian-Xiong Liu

Site Principal Investigator, Director of Department of Cardiology

Chengdu Sencond People's Hospital

No.10, Qingyun South Street, Jinjiang District, Chengdu, 610017, Sichuan, China

Email: 18908178061@163.com

31. People's Hospital of Zhengzhou

Prof. Heng-Liang Liu

People's Hospital of Zhengzhou

No. 33 Huanghe Road, Zhengzhou, 450003, Henan,China

Email: 13937115282@163.com

DATA MONITORING COMMITTEE (DMC)

Prof. Chun-Ti Shen (Chair), Changzhou TCM Hospital Affiliated to Nanjing University of Chinese Medicine

Prof. Qi-Guang Chen (Member, independent statistician), Southeast University

Prof. Hua-Qiang Zhang (Member), Jiangsu Provincial Commission of Health and Family Planning

Dr. Hong-Wu Wang (Member), Tianjin University of Traditional Chinese Medicine

Dr. Xiao-Chun Sun (Member), Beijing Kelunrui Pharmaceutical Technology Co. Ltd.

Prof. Cheng-Shu Wang (Member), Zhongda Hospital Southeast University

Dr. Hai-Xia Dang (Member), China Academy of Chinese Medical Sciences

Dr. Jun Liu (Member), Institute of Basic Research in Clinical Medicine, China Academy of Chinese Medical Sciences

PROTOCOL STATISTICIANS

Prof. Bi-Wei Chen, Ph.D., School of Public Health, Southeast University

Dijia Qiao 87, Nanjing,210009, Jiangsu,China

E-mail: drchenbw@126.com

TABLE OF CONTENTS

EXECUTIVE COMMITTEE CHAIR - 36 -
PRINCIPLE INVESTIGATORS - 36 -
PARTICIPATING SITES AND INVESTIGATOR - 37 -
DATA MONITORING COMMITTEE (DMC) - 45 -
PROTOCOL STATISTICIANS - 45 -
SUMMARY OF CHANGES - 48 -
PROTOCOL SYNOPSIS - 49 -
1. BACKGROUND INFORMATION - 52 -
2. OBJECTIVES - 53 -
3. STUDY DESIGN - 53 -
3.1 Overview - 53 -
3.2 Sample size - 53 -
3.3 Randomization - 55 -
3.4 Blinding - 55 -
3.5 Interim analysis - 55 -
4．SUBJECTS - 56 -
4.1 Diagnostic criteria - 56 -
4.2 Inclusion Criteria - 58 -
4.3 Exclusion Criteria - 58 -
4.4 Withdrawal/dropout criteria - 59 -
4.5 Criteria for study termination - 60 -
4.6 Criteria for subject discontinuation - 60 -
5 INTERVENTION - 61 -
5.1 Optimal medical therapy - 61 -
5.2 Treatment regimens - 62 -
5.3 Duration and follow-up - 62 -
5.4 Concomitant medications - 62 -
6 OUTCOMES MEASUREMENTS - 63 -
6.1 Primary outcome - 63 -
6.2 Secondary Outcomes - 63 -
6.3 Other Pre-specified Efficacy Outcomes - 66 -
6.4 Safety Outcomes - 66 -
6.5 Study Schema - 67 -
7 ADVERSE EVENTS - 68 -
7.1 Definition - 68 -
7.2 Recording - 69 -
7.3 Severity of an AE - 69 -
7.4 Evaluation the correlation between AEs and study drugs - 70 -
7.5 Handling of SAEs - 70 -
7.6 Opening and handling of emergency envelops - 71 -
7.7 Follow-up of unresolved AEs - 71 -
8. DATA COLLECTION AND MANAGEMENT - 71 -
8.1 Establishment of Three Committees - 71 -

8.2 Data Management - 72 -
8.3 Data Report - 72 -
8.4 Data Auditing and Blinding Review - 73 -
8.5 Database Locking - 73 -
9. STATISTICAL CONSIDERATIONS - 74 -
9.1 Study Populations - 74 -
9.2 Statistical Analyses - 74 -
9.3 Statistical Analysis Plan (SAP) - 75 -
10 ETHICAL ASPECTS - 75 -
REFERENCES - 76 -

SUMMARY OF CHANGES

All changes in this version appear in blue and italic type. Major changes include the following:

2) In Participating Sites and Investigator, 6 sites and corresponding investigators initially took part in this trail and at last 31 sites and corresponding investigators all over China have participated in our study.
3) After the first interim analysis, the DMC recommend that the ETT test be omitted for better patient compliance and higher feasibility of the trial, as many participants were reluctant to complete the test due to the uncomfortable experience during ETT in China. This makes the changes on 6.3 Other Pre-specified efficacy outcomes and 6.5 Study Schema.
4) After the second interim analysis, the sample size is expanded to 920. The detailed information about the process of this change could be see in 3.2.2 Sample size re-estimation.

PROTOCOL SYNOPSIS

TITLE:

Danhong Injection for the treatment of chronic stable angina (Phase IV): an adaptive-design, randomized, multi-center, double-blind, placebo-controlled clinical trial

DESIGN:

A multi-center, randomized, parallel-controlled, double-blind, adaptive clinical trial (superiority design)

NUMBER OF SITES:

31 sites

SAMPLE SIZE:

A total of 870 study participants with angina pectoris was planned initially. *And the sample size was enlarged to 920 by the DMC based on the results of the second interim analyses.*

POPULATION:

Patients aged 18 to 70 years who suffered from chronic stable angina with a clinical diagnosis of "Xueyu-Zheng" (Blood Stasis Syndrome), CCS angina class Ⅱ or Ⅲ were eligible for inclusion in the study.

RANDOMIZATION：

Randomization to Danhong Injection (DHI) group or control group (0.9% normal saline as placebo) will be in a 2:1 ratio

REGIMEN:

Optimal medical therapy (OMT) was given to all participants throughout the trial, including anti-platelet

agents (aspirin, 75-100 mg, once a day, and/or clopidogrel, 75 mg, once a day), lipid-lowering agents (atorvastatin, 10-20 mg, once a day, or simvastatin, 20-40 mg, once a day) and anti-angina agents (β-blockers, long-acting nitrates, or calcium channel blockers).

DHI group: OMT + DHI (40 ml, qd) plus 0.9% normal saline (250 ml, ivgtt, qd)

Control group: OMT + placebo of DHI (0.9% normal saline, 40ml, qd) plus 0.9% normal saline (250 ml, ivgtt, qd)

TREATMENT DURATION:

14 days

FOLLOWING UP

76 days after treatment

OUTCOME MEASURES:

Efficacy:

The primary efficacy endpoint of this study is:

Proportions of patients who had a clinically significant change on the angina-frequency scale in the Seattle Angina Questionnaire (SAQ) at Day 30

Secondary efficacy endpoints are:

(1).The total score of symptoms in a questionnaire related to traditional Chinese medicine;

(2). The proportion of patients who experience clinically significant changes in the other four Seattle Angina Questionnaire domains;

(3) The frequency of anginal attacks;

(4) Angina grade, according to the Canadian Cardiovascular Society (CCS) Angina Grading Scale;

(5) Consumption of short-acting nitrates;

(6) Changes in electrocardiogram results;

(7) Changes in serum lipid levels;

(8) Changes in high-sensitivity C-reactive protein levels;

(9) Changes in the platelet aggregation rate.

(10) Incidence of new-onset major vascular events within 90 days.

Other pre-specified efficacy endpoints are:

~~*(1) Changes in total exercise duration (TED) during exercise tolerance testing (ETT). from baseline to Day 14 in 290 patients selected after first interim analysis;*~~

~~*(2) Changes in time to 1mm st-segment depression during exercise tolerance testing (ETT) from baseline to Day 14 in 290 patients selected after first interim analysis;*~~

~~*(3)*~~ Changes in the micro-RNA and mRNA profiles of 60 patients selected at the Chinese PLA General Hospital and Xuanwu Hospital Capital Medical University.

Safety:

(1) Overall mortality within 90 days;

(2) Incidence of severe haemorrhages within 90 days;

(3) Incidence of moderate haemorrhages within 90 days;

(4) Incidence of adverse and serious adverse events.

2. BACKGROUND INFORMATION

Stable ischemic heart disease is a leading cause of death worldwide, and nearly 58% of patients with coronary artery disease were suffering from chronic stable angina[1]. Current treatment options including including pharmacotherapy (as organic nitrates, β-blockers, calcium channel antagonists), revascularization, lifestyle management and several alternative procedures, aims to reduce the risk of mortality and morbid events and to reduce symptoms[2]. For patients, it is often the latter that is of greater concern[3]. Despite these multiple treatment options[2], a high proportion of patients with stable angina remains symptomatic and their quality of life is impaired[4,5]. Moreover, several observational studies have shown that angina symptoms such as physical limitation and angina frequency are predictive indicators of mortality and acute coronary syndrome (ACS) hospitalizations[6-8]. Therefore, we need more therapies for patients with chronic angina in order to prevent the episode of the angina for the improvement of their quality of life.

Danhong injection (DHI), which is extracted from Danshen (Radix Salviae miltiorrhizae) and Honghua (Flos carthami), is widely used to treat the coronary heart disease. DHI, the profile of which has been analyzed using HPLC, is approved as a Chinese medicinal product for the treatment of various ischemic diseases by the State Food and Drug Administration of China (SFDA) in 2002. Some clinical studies have demonstrated that Danhong injection might be an effective and safe treatment option for the management of coronary heart disease[9,10.] However, the methodological quality of most previous studies was assessed to be, in general, low[10]. No critically appraised evidence, such as a well-designed randomized controlled trial, is available to provide a high level of evidence to justify the clinical use and recommendation of DHI. In this study, we will conduct a randomized, multicentre, double-blind, placebo-controlled trial to investigate the efficacy and safety of DHI in patients with

chronic stable angina.

2. OBJECTIVES

The objective of this study is mainly to evaluate the effect of DHI in preventing the episode of chronic stable angina and improving the angina-specific quality of life, and the safety of DHI.

3. STUDY DESIGN

3.1 Overview

This study is a three-stage adaptive-design, randomized, multicentre, double-blind, placebo-controlled trial. The number of subjects is initially estimated to be 870 and randomly assigned to be 582 in DHI group and 288 in the control group. Both groups received optimal medical therapy as recent guideline advocated. Participants in the DHI group are treated by DHI (40 ml, qd) plus 0.9% normal saline (250 ml, ivgtt, qd), while participants in the control group are treated by DHI placebo (0.9% normal saline, 40ml, qd) plus 0.9% normal saline (250 ml, ivgtt, qd).

3.2 Sample size

3.2.1 Initial estimation of sample size

According to the previous study[11], the proportion of patients who had clinically significant change defined as at least 20-point improvement in Seattle Angina Questionnaire angina frequency (SAQAF) score was 30%, after standard conventional therapy for a month. In this trial, we adopt an adaptive design by using the statistical sample size calculation software EAST5.2. It is hypothesized that an increase of at least 10% is of clinical significance for the DHI group; therefore, the number of subjects is initially estimated to be 726 (one-sided test, $\alpha=0.05$, $\beta=0.15$). To allow for a 20% dropout rate, a total

of 870 patients will be recruited. As patients will be randomized into the DHI group or control group in a ratio of 2:1, the number of participants in the DHI group is 582 and that in the control group is 288. According to the adaptive design, the sample size may be adjusted based on the results of two interim analyses, which will be carried out after one third (288) and two thirds (582) of patients have completed the trial, respectively.

3.2.2 Sample size re-estimation

In the first interim analysis, 288 patients were enrolled in the trial and 275 had the primary outcome. In the group A, 105 patients (57.07%, 105/183) had a clinically significant change in SAQAF at Day 30, while in the group B, 40 (43.96%, 40/91) got the clinically significant change in SAQAF (Z=2.063), and thus, the difference was not statistically significant between the two groups (Lan-DeMets spending function boundary not be crossed). Conditional power (CP) was calculated as 0.984 using EAST 5.2, and thus, DMC decided that the sample size should not be re-estimated.

In the second interim analysis, 576 patients were enrolled in the trial and 550 had the primary outcome. In the group A, 205 patients (56.32%, 205/364) had a clinically significant change in SAQAF at Day 30, while in the group B, 78 (41.94%,78/186) got the clinically significant change in SAQAF (Z=3.228), and the difference was statistically significant between the two groups (Lan-DeMets spending function boundary was crossed). Indeed, the trial could be stopped in advance because of the good efficacy of DHI according to the suggest of EAST 5.2. However, since there was no significant difference in the proportion of patients who have a clinically significant change in SAQAF at Day 90 between the two groups (64.64% vs. 55.91%, P= 0.0513), DMC determined to continue the trial and re-estimate the total sample size as 920 (one-sided test, α=0.05, β=0.1) using the above data at Day 90

with approximate 10% drop-out.

3.3 Randomization

All eligible patients who consent to participation will be randomized into either the Danhong injection or the placebo group in a 2:1 ratio. Randomization will be conducted using a clinical information management system (Brightech, Somerset, USA). This system automatically randomizes patients and generates a randomization number with a message noting their assigned treatment. In addition, randomization will be stratified based on whether a patient received standard conventional therapy for more than 1 week prior to study initiation.

3.4 Blinding

Participants and research personnel will be blinded to Danhong injection therapy or placebo treatment group assignments until the study has concluded. Because the colour of Danhong injection and 0.9 % saline are different,the dropping bottles will be wrapped in sealed shaded bags, and brown infusion devices will be used for infusion. These procedures will be implemented by two professional nurses who will be required to sign a confidentiality agreement before study initiation and not to contact each other. One of the professional nurses will be in charge of preparing the drugs in a special transfusion room and sealing the infusion bottles with shaded brown bags. The other nurse will take the prepared drugs from the transfusion room to the infusion nurse and supervise the infusion process to ensure that the allocation of the drugs is blinded to the patients (the shaded brown bags will not be unwrapped during infusion and will be checked for integrity after infusion).

3.5 Interim analysis

Because this is an adaptive trial, two interim analyses are prospectively planned, and these will be performed in a blinded manner after one-third and two-thirds of the patients, respectively, have

completed the trial. The statistical results of the interim analyses will be relayed to the data monitoring committee, who will decide on the re-estimation of sample size and determine whether any subsequent modifications must be made to the trial protocol. Because the two interim analyses may lead to an increased possibility of a type I error, we will apply the Lan-DeMets alpha spending function with an O'Brien-Fleming boundary to adjust the results.

4. SUBJECTS

4.1 Diagnostic criteria

4.1.1 Diagnostic criteria in Western Medicine

The diagnostic criteria for chronic stable angina are determined according to the Chinese Guidelines for the Diagnosis and Treatment of Chronic Stable Angina (2007)[2], the ACC/AHA Guideline Update for the Management of Patients With Chronic Stable Angina (2002)[12], and the European Society of Cardiology Guidelines for the Management of Stable Angina Pectoris[13].

Stable angina is characterized by transient episodes of chest pain precipitated by exercise or by other situations resulting in an increased myocardial oxygen demand. The pain usually disappears rapidly with rest or with sublingual nitroglycerin. The stable angina has 1 month's duration or more.

Patients must fulfill one of the following conditions:

(1) a history of myocardial infarction and ST-T changes;

(2) stenosis of more than 50 % in at least one major epicardial coronary artery, as shown by coronary angiography or computed tomography Angiography;

(3) coronary heart disease confirmed by radionuclide angiocardiography.

4.1.2 Grading of angina pectoris

According to the "Canadian Cardiovascular Society (CCS) grading of angina pectoris", the

severity of angina can be graded as follows[14]:

Class I: Ordinary physical activity does not cause angina, such as walking and climbing stairs. Angina with strenuous or rapid or prolonged exertion at work or recreation.

Class II: Slight limitation of ordinary activity. Walking or climbing stairs rapidly, walking uphill, walking or stair climbing after meals, or in cold, in wind or under emotional stress, or only during the few hours after awakening. Walking more than two blocks on the level and climbing more than one flight of ordinary stairs at a normal pace and in normal conditions.

Class III: Marked limitation of ordinary physical activity. Walking one or two blocks on the level and climbing one flight of stairs in normal conditions and at normal pace.

Class IV: Inability to carry out any physical activity without discomfort; angina may be present at rest.

4.1.3. Diagnostic criteria in Traditional Chinese medicine

Traditional Chinese medicinediagnostic criteria are determined according to the Guidelines for Clinical Research of New Drugs of Traditional Chinese Medicine (2002)[15].

We will choose the patients with the a clinical diagnosis of “Xueyu Zheng” (blood stasis syndrome), which is defined as a score of at least 15 on the Chinese Medicine Symptom Scale of “Xueyu Zheng” for angina patients.

The Chinese Medicine Symptom Scale of “Xueyu Zheng” includes the following items:

(1) chest pain (0–10);

(2) chest distress (0–10);

(3) palpitation (0–5);

(4) purple or dark lips (0–5);

(5) purple or dark tongue (0–5);

(6) unsmooth pulse (0–5).

If the patient has a certain above symptom, corresponding 10 or 5 score will be in this symptom; otherwise, 0 for the symptom. The total score of at least 15 will be determined as “Xueyu Zheng”.

4.2 Inclusion Criteria

(1). Female or male inpatients.

(2). Age: 18–70 years.

(3). Patients with a clinical diagnosis of chronic stable angina.

(4). Patients with a clinical diagnosis of “Xueyu Zheng” (blood stasis syndrome).

(5). Patients with moderate angina pectoris, which is defined as Grade II or III on the Canadian Cardiovascular Society Angina Grading Scale.

(6). Patient is willing to voluntarily participate and to sign a written informed consent document.

4.3 Exclusion Criteria

(1). Women who are pregnant, lactating, having a positive pregnancy test, or having a menstrual period at baseline.

(2). Women with childbearing potential disagree with using contraception during the treatment period.

(3). Patients with severe complications that would complicate the condition, as assessed by the investigator, including liver or renal dysfunction, severe cardiopulmonary dysfunction, pulmonary hypertension, chronic obstructive pulmonary disease, a history of epilepsy or cerebral haemorrhage.

(4). Patients were angina-free during the run-in period without taking any drug.

(5). Patients experienced myocardial infarction or who were classified as Grade IV on the Canadian Cardiovascular Society Angina Grading Scale within the preceding 3 months.

(6). Patients with chest pain that is caused by any other disease (e.g., acute myocardial infarction,

severe neurosis, menopausal syndrome or hyperthyroidism).

(7). Patients with a history of drug-induced bleeding or a history of bleeding caused by warfarin.

(8). Patients with a history of haematopoietic disorder.

(9). Patients have had surgery within the previous 4 weeks or who have a haemorrhagic tendency.

(10). Patients who are participating in other trials or who have participated in other trials within the past 3 months.

(11). Patients with a history of allergy or with a known or suspected allergy to the study drug.

(12). Patients with a known or suspected history of alcohol or drug abuse within the past 2 years.

(13). Patients with a mental disorder.

(14). Patients who are unable to participate in the study, as judged by the investigator.

(15). Family members or relatives of the study centre staff.

4.4 Withdrawal/dropout criteria

(10) Those who did not fulfill the eligibility criteria but were included by mistake;

(11) Those who fulfilled the eligibility criteria but did not take any study drug after randomization;

(12) Those with poor compliance that might interfere with the efficacy and safety evaluation;

(13) Those who experienced serious AEs, complications or special physiological changes, making it inappropriate to continue with the trial;

(14) Individual cases that were unblinded prematurely;

(15) Those who discontinued the trial voluntarily;

(16) Those who used any disallowed concomitant medication, especially those that had an obvious effect on the study drug and might interfere with the efficacy and safety evaluation;

(17) Those who discontinued or lost to follow-up for any other reasons or died during the trial;

(18) Those with incomplete data that might interfere with the efficacy and safety evaluation.

For all withdrawal/dropout cases, the potential reasons must be recorded. Patients who experienced any AE during the trial must be included in the statistical analysis of adverse drug reactions. Those who have taken the study drug for at least 1 week should be included in the statistical analysis of drug efficacy.

4.5 Criteria for study termination

Circumstances that may warrant termination include, but are not limited to:

(6) Identification of unexpected, significant, or unacceptable risk to subjects;

(7) Poor efficacy or no efficacy of the study drug;

(8) Major mistakes in the trial protocol;

(9) Insufficient adherence to protocol requirements;

(10) Upon sponsor's request (e.g. economic or administrative reasons).

4.6 Criteria for subject discontinuation

(4) Any allergic reaction or AE occurs such that continued participation in the study would not be in the best interest of the subject at the discretion of the treating physician;

(5) Worsening of clinical conditions occurs such that continued participation in the study would not be in the best interest of the subject at the discretion of the treating physician; considered as ineffective cases;

(6) Subjects are free to withdraw from participation in the study at any time upon request.

In any case, every effort must be made to determine why patients discontinued the study treatment prematurely, which may include but are not limited to poor confidence in efficacy, occurrence of AEs, SAEs, or other serious complications, worsening of symptoms such that emergent intervention is

required. As for those who withdrew from participation in the study or lost to follow-up, it is essential to investigate the potential reasons via telephone. Investigators should record in detail when and how the last dose was administered, the efficacy and safety parameters at and after discontinuation, the relationship between discontinuation or withdrawal and the study drug, and the potential impact of discontinuation cases on the final conclusion of the study. Moreover, case report form (CRF) of withdrawal/dropout cases should also be completed and their original data should also be documented and stored in a secure manner.

5 INTERVENTION

5.1 Optimal medical therapy

Optimal medical therapy will be provided to all of the included participants throughout the trial, in strict accordance with the Chinese Guidelines for the Diagnosis and Treatment of Chronic Stable Angina (2007) [2].

Optimal medical therapy includes:

(1) Antiplatelet agents: aspirin (75-100 mg, once per day) or clopidogrel (if the patient is intolerant to aspirin). Patients with a history of percutaneous coronary intervention will be prescribed both of these agents.

(2) Lipid-lowering agents (statins): atorvastatin (10-20 mg, once per day) or simvastatin (20-40 mg, once per day).

(3) Anti-angina agents: β-blockers (metoprolol 50-200 mg, once per day, or analogous agents); long-acting nitrates (isosorbide mononitrate 40-60 mg, once per day); or calcium channel blockers (amlodipine 5-20 mg, once per day).

(4) Patients with diabetes and hypertension will be advised to take angiotensin-converting enzyme

inhibitors or angiotensin-receptor blockers (e.g., lisinopril 10-20 mg, once per day, or losartan 50 mg, once per day) as a secondary preventive measure for chronic stable angina.

All of these basic treatments will be recorded in detail in the patients' medical records as well as in their e-CRFs.

Participants who received optimal medical therapy for more than 1 week prior to study initiation will be randomized directly into one of the groups; otherwise, a run-in period of 1 week of optimal medical therapy will be performed before randomization.

5.2 Treatment regimens

DHI group: Participants will be treated using Danhong injection (40 ml per day) plus 0.9 % normal saline (250 ml intravenously per day).

Control group: Participants will be treated using placebo (0.9 % normal saline, 40 ml per day) plus 0.9 % normal saline (250 ml intravenously per day).

5.3 Duration and follow-up

All of the included patients will undergo a 2-week treatment regimen and a 76-day follow-up period.

5.4 Concomitant medications

(1) Sublingual nitroglycerin tablet (0.5 mg per tablet, provided by Beijing Yimin Pharmaceutical Co., Ltd.)is permitted in case of an angina attack, and this dose can be repeated approximately every 5 minutes until the angina is relieved. However, if the angina persists after three doses, the patient should be transported to hospital immediately for further medical treatment. Patients will be asked to record the details of administration times, the number of doses and the dosages of nitroglycerin in a patient diary, which will be collected by the investigators at a subsequent study visit.

(2) Other WM medications targeting the treatment of angina or CHD will be disallowed during the trial.

(3) Participants are not allowed to take any other Chinese herbal medicines or Chinese patent medicines that have the effect of promoting blood circulation or removing blood stasis throughout the study period.

(4) If other medication or therapy is required to treat the concomitant diseases, then the name of the drug or therapy, actual dosage, dosing frequency and start/stop time should be well-documented.

6 OUTCOMES MEASUREMENTS

6.1 Primary outcome

Angina-specific health status will be assessed at baseline, Day 30, 60, and 90. Each assessment will be performed with the use of the Seattle Angina Questionnaire (SAQ), a 19-item self-administered questionnaire that measures 5 domains of CAD-related health status: physical limitation(PL), angina frequency(AF), treatment satisfaction(TS), and disease perception/quality of life(DP). The total score ranges from 0 to 100; and higher scores indicate better health status.[11]

The primary outcome is the proportion of patients who have a clinically significant change in Seattle Angina Questionnaire angina frequency (SAQAF) score at Day 30.

A clinically significant change in each scale of SAQ was defined as a difference of 8 points or more on the physical-limitation scale, 25 or more on the angina-stability scale, 20 or more on the angina-frequency scale, 12 or more on the treatment-satisfaction scale, and 16 or more on the quality-of-life scale, respectively.[11]

6.2 Secondary Outcomes

6.2.1 The total score of symptoms in a questionnaire related to traditional Chinese medicine .

The efficacy evaluation scale of Chinese Medicine Symptom in “Xueyu Zheng” (Table 1) will be used to assess the condition of patients with Xueyu Zheng at each visit (Day 0, Day 7, Day 14, Day 30, Day 60, Day 90).

Table 1 The efficacy evaluation scale of Chinese Medicine Symptom in “Xueyu Zheng”

Symptom	Visual Analogue Scale*	Score
Chest pain	0-----------------------------------10	
Chest distress	0-----------------------------------10	
Palpitation	0-----------------------------------5	
Purple or dark lips	0-----------------------------------5	
Purple or dark tongue	0-----------------------------------5	
Unsmooth pulse	No--0 Yes---5	

* According to the severity of the symptoms, the more severe the symptom is, the higher the score be determined. If the symptom is disappeared, the score will be recorded as 0.

The mean total score of symptoms in a questionnaire of *Xueyu Zheng* will be analyzed and compared between two groups at each visit. Moreover, the proportions of patients with syndrome improvement in *Xueyue Zheng* between the two groups will be recorded at each visit. Significant syndrome improvement is defined as at least 30% reduction in the *Xueyue Zheng* score[15].

6.2.2 The proportion of patients who experience clinically significant changes in the other four Seattle Angina Questionnaire domains (physical limitation, angina stability, treatment satisfaction, and disease perception/quality of life)[11].

At Day 30, 60, 90, investigators will observe the proportions of patients in each treatment group who were angina-free. Angina-free is defined as a score of 100 in the angina frequency score on the SAQ.

6.2.3 The frequency of angina attacks.

The patients will be asked to record their angina attacks in the diary. At each visit (Day 7, 14, 30, 60, 90), the investigator will collect the diary and record it onto the electronic case report forms (e-CRFs).

6.2.4 Angina grade, according to the Canadian Cardiovascular Society (CCS) Angina Grading Scale.

At each visit, the angina grade of each patient will be evaluated according to the Canadian Cardiovascular Society (CCS) Angina Grading Scale. The proportion of patients who have the improvement of the Canadian Cardiovascular Society (CCS) Angina Grading Scale will be compared between two groups at each visit (Day 7, 14, 30, 60, 90), which is defined that the CCS grade change to Grade I or decrease 1 grade.

6.2.5 Consumption of short-acting nitrates.

The patients will be asked to record their usage of short-acting nitrates in the diary. At each visit (Day 0, 7, 14, 30, 60, 90), the investigator will collect the diary and record it onto the e-CRF.

6.2.6 Changes in electrocardiogram results.

The 12-lead electrocardiogram (ECG) will be tested at each visit (Day 0, 7, 14, 30, 60, 90). The result will be evaluated by two independent ECG doctors. The proportion of patients with normal ECG recordings will be compared between the two groups at each visit. The "normal ECG" in our trial is defined as including (1) normal sinus rhythm (each P wave is followed by a QRS and P wave rate 60 - 100 bpm with <10% variation); (2) normal P waves (height < 2.5 mm in lead II and width < 0.11 s in lead II); (3) normal PR interval (0.12 to 0.20 s); (4) normal QRS complex (< 0.12 s duration, no pathological Q waves and no evidence of left or right ventricular hypertrophy); (5) normal QT interval (0.42 s); (6) normal ST segment without any elevation or depression; (7) normal T wave; and (8) normal U wave.

6.2.7 Changes in serum lipid levels at Day 14.

6.2.8 Changes in high-sensitivity C-reactive protein levels at Day 14.

6.2.9 Changes in the platelet aggregation rate at Day 14.

6.3 Other Pre-specified Efficacy Outcomes

6.3.1 Initial pre-specified efficacy outcomes

(1) Changes in total exercise duration (TED) during exercise tolerance testing (ETT) from baseline to Day 14 in 290 patients selected after first interim analysis;

(2) Changes in time to 1mm st-segment depression during exercise tolerance testing (ETT) from baseline to Day 14 in 290 patients selected after first interim analysis;

(3) Changes in the micro-RNA and mRNA profiles of 60 patients selected at the Chinese PLA General Hospital and Xuanwu Hospital Capital Medical University.

6.3.2 Pre-specified efficacy outcomes after first interim analysis

Changes in the micro-RNA and mRNA profiles of 60 patients selected at the Chinese PLA General Hospital and Xuanwu Hospital Capital Medical University.

After the first interim analysis, the DMC recommend that the ETT test be omitted for better patient compliance and higher feasibility of the trial, as many participants were reluctant to complete the test due to the uncomfortable experience during ETT in China.

6.4 Safety Outcomes

6.4.1 Incidence of new-onset major vascular events within 90 days.

Major vascular events include death induced by cardiac or cerebral vascular disorders , myocardial infarction, stroke, transient ischemic attack (TIA), coronary intervention (including stent thrombosis), peripheral vascular intervention, hospitalization for unstable angina, and acute heart failure (HF) events.

6.4.2 Overall mortality within 90 days.

6.4.3 Incidence of severe haemorrhages within 90 days.

Severe haemorrhages includes fatal bleeding, primary intracranial hemorrhage, post-traumatic symptomatic intracranial hemorrhage, or bleeding requiring transfusion, infusion, use of vasoconstrictor drugs and surgical intervention.

6.4.4 Incidence of moderate haemorrhages within 90 days.

Moderate haemorrhages includes bleeding requiring infusion but do not reach severe bleeding, absolute reduction in hemoglobin/hematocrit, severe disability induced by bleeding, intraocular hemorrhage with severe visual field defects, etc.

6.4.5 Incidence of adverse and serious adverse events (Section 7).

6.5 Study Schema

6.5.1 Initial study schema

Period	Run-in period	Treatment period			Follow-up period		
Day	-7	0	7	14	30	60	90
Informed consent	×	×					
Inclusion/exclusion criteria	×	×					
Medical history	×	×					
Medical examination	×						
Combined disease treatment	×	×	×	×	×	×	×
Outcomes							
SAQ		×			×	×	×
Symptoms questionnaire of TCM		×	×	×	×	×	×
Frequency of anginal attack per week	×	×	×	×	×	×	×
CCS grade		×	×	×	×	×	×
Consumption of short-acting nitrates	×	×	×	×	×	×	×
ECG		×	×	×	×	×	×
Serum lipids		×		×			
Hs-CRP		×		×			
Platelet aggregation rate		×		×			
New-onset major vascular events							×
Overall mortality							×
Incidence of severe hemorrhages							×
Incidence of moderate hemorrhages							×
AEs and SAEs			×	×	×	×	×
ETT (if necessary after 1st interim analysis)		×		×			
Profiles of micro-RNA in 60 patients in certain centers		×		×			×
Profiles of mRNA in 60 patients in certain centers		×		×			×

6.5.2 Study schema changed after the first interim analysis

Period	*Run-in period*	*Treatment period*			*Follow-up period*		
Day	*-7*	*0*	*7*	*14*	*30*	*60*	*90*
Informed consent	×	×					
Inclusion/exclusion criteria	×	×					
Medical history	×	×					
Medical examination	×						
Combined disease treatment	×	×	×	×	×	×	×
Outcomes							
SAQ		×			×	×	×
Symptoms questionnaire of TCM		×	×	×	×	×	×
Frequency of anginal attack per week	×	×	×	×	×	×	×
CCS grade		×	×	×	×	×	×
Consumption of short-acting nitrates	×	×	×	×	×	×	×
ECG		×	×	×	×	×	×
Serum lipids		×		×			
Hs-CRP		×		×			
Platelet aggregation rate		×		×			
New-onset major vascular events							×
Overall mortality							×
Incidence of severe hemorrhages							×
Incidence of moderate hemorrhages							×
AEs and SAEs			×	×	×	×	×
Profiles of micro-RNA in 60 patients in certain centers		×		×			×
Profiles of mRNA in 60 patients in certain centers		×		×			×

7 ADVERSE EVENTS

7.1 Definition

Adverse event (AE): An AE is any untoward medical occurrence in a patient or clinical investigation subject administered a pharmaceutical product and that does not necessarily have a causal relationship with this treatment. An AE can therefore be any unfavorable and unintended sign (including an abnormal laboratory finding), symptom, or disease temporally associated with the use of a medicinal (investigational) product, whether or not related to the medicinal (investigational) product. Pre-existing conditions which worsen during a study are also to be reported as AEs.

Serious Adverse Event (SAE): A SAE is any experience that suggests a significant hazard,

contraindication, side effect or precaution. It is any AE that at any dose fulfils at least one of the following criteria:

– is fatal;

– is life-threatening;

– required in-patient hospitalization or prolongation of existing hospitalization;

– results in persistent or significant disability/incapacity;

– is a congenital anomaly/birth defect;

– is medically significant or requires intervention to prevent one or other of the outcomes listed above.

Adverse drug reaction (ADR): An ADR is any unintended harm or injury associated with the use of given medications at a normal dosage during normal use. In clinical trials of any new drug or any new usage of a certain drug, any unintended harm or injury that has a causal relationship to the use of the drug should also be considered as an ADR.

7.2 Recording

For all AEs encountered during the clinical study, the following must be assessed and recorded on the adverse events form of the e-CRF: intensity, relationship to study drug, action taken regarding study drug, and outcome to date. All AEs, especially those for which the relationship to test drug is not "unrelated", should be followed up until they have returned to baseline status or stabilized. The follow-up procedures could be performed in clinic visits or during hospitalization, or via home visit, telephone or mail, depending on the severity of AEs.

7.3 Severity of an AE

Mild: events require minimal or no treatment and do not interfere with the subject's daily

activities.

Moderate: events result in a low level of inconvenience or concern with the therapeutic measures. Moderate events may cause some interference with functioning.

Severe: events interrupt a subject's usual daily activity and may require systemic drug therapy or other treatment. Severe events are usually incapacitating.

7.4 Evaluation the correlation between AEs and study drugs

Based on the current 6-level evaluation standard of the China National Center for Adverse Drug Reaction Monitoring, according to the answers to 5-iterm questionnaire, the correlation between AEs and study drugs can be categorized as to definitely related, probably related, possibly related, unlikely related, to be evaluated, and unable to evaluate (Table 2) . The adverse drug reaction was determined as "definitely related", "probably related", " possibly related".

Table 2 The assessment of the causality between adverse events and observed drug

<table>
<tr><th rowspan="2">Item*</th><th colspan="6">Index</th></tr>
<tr><th>1= definitely related</th><th>2= probably related</th><th>3= possibly related</th><th>4= unlikely related</th><th>5= to be evaluated</th><th>6= unable to evaluate</th></tr>
<tr><td>A reasonable timing relationship[1]</td><td>+</td><td>+</td><td>+</td><td>-</td><td rowspan="5">Additional detailed information to be evaluated</td><td rowspan="5">The necessary information for the evaluation is not available</td></tr>
<tr><td>Belonging to the Known ADR of the drug [2]</td><td>+</td><td>+</td><td>±</td><td>-</td></tr>
<tr><td>Relief after stopping the drug[3]</td><td>+</td><td>+</td><td>± ?</td><td>± ?</td></tr>
<tr><td>Recurrent AE after re-administration[4]</td><td>+</td><td>?</td><td>?</td><td>?</td></tr>
<tr><td>Suspected adverse reaction could be explained with other certain reasons[5]</td><td>-</td><td>-</td><td>± ?</td><td>± ?</td></tr>
</table>

*The 5-iterm questionnaire to assess the causality between adverse events and observed drug is listed in the items 1 to 5, detailed as following:

2. Is there a reasonable relationship between the time of starting the drug and the time of occurrence of the suspected adverse reaction?

2. Is the suspected adverse drug reaction consistent with the types of adverse reaction known to the drug?

3.Could the suspected adverse reaction be alleviated or disappeared after stopping the drug or reducing the dosage?

4. Dose the same adverse event occur again after given the same drug again?

5. Could the suspected adverse reaction be explained by the patient's pathological condition, combined medication, combined therapy, and used therapy etc?

7.5 Handling of SAEs

All SAEs should be managed properly and timely, and recorded on the appropriate SAE eCRF. Any AE considered serious by the investigator or which meets the aforementioned criteria must be submitted on a signed and dated SAE form to the sponsor, ethics review committee and relevant authorities within 24 hours of site awareness. And the sponsor should also inform all study sites of such SAEs in a timely manner to ensure subjects' safety in accordance with applicable laws and regulations.

Fatal or life-threatening events considered to be caused by the study drugs must be reported immediately to the sponsor and relevant authorities via telephone, fax, EMS or E-mail.

7.6 Opening and handling of emergency envelops

The emergency envelope provided along with each test drug in its package should only be opened in case of a serious complication or a serious adverse event, which is called emergency unblinding. Once unblinded, the patient should be withdrawn from the study and regarded as a dropout case. Information about when, where and why the emergency unblinding occurs as well as each personnel's signature should also be documented.

7.7 Follow-up of unresolved AEs

All AEs should be followed up until they have been resolved, stabilized or returned to baseline status.

9. DATA COLLECTION AND MANAGEMENT

8.1 Establishment of Three Committees

To ensure the effectiveness and integrity of the trial design, three committees have been established by the multicentre trial coordination group: the clinical trial guidance committee (Executive committee), the data monitoring committee (DMC) and the outcome evaluation committee. The first committee is responsible for the study design and the implementation process. The data monitoring

committee will supervise the integrity and accuracy of data collection to control its quality. The outcome evaluation committee will evaluate key outcomes (including outcome measurements and adverse events) based on their clinical expertise.

8.2 Data Management

(1) All data will be recorded by trained clinical investigators using standardized e-CRFs and Brightech clinical information management systems (Brightech, Somerset, NJ, USA).

(2) The accuracy and reliability of the data will be ensured by the study monitor, who will verify and cross-check the e-CRFs against the investigator's records (source document verification), using the maintenance of a drug dispensing log by the investigator.

(3) Front-end checks in the e-CRFs and back-end checks in the clinical information management system will be used as a validation check of the data.

(4) If there are any discrepancies in the e-CRFs, the results will be sent to the investigator for resolution.

(5)The results of the analysis must not be released with individual identification of the subjects until the database is closed.

8.3 Data Report

Data report contains the aspects as followed: (1) members of the project; (2) disagreement from the primary data management plan; (3) actual finish time of every project; (4) problems and the solution during the data management (if have any); (5) reconstruction of the database (if have any); (6) distribution of the participants; (7) participants who disobey the trial protocol; (7) classifying plan of the statistical analysis population. Data report will be performed monthly and then send to DMC since the first entry of the e-CRF.

8.4 Data Auditing and Blinding Review

When the data checking is finished, a data auditing and blinding review meeting will be hold. On the meeting, the data administrators, statisticians, researchers, clinical inspectors, and other related members would have a discussion on the following items according to the data management report and the data lists:

•Distribution of the participants;

•Protocol disobeying or not;

•Possible outlier;

•Baseline data;

•Outcomes;

•Statistical analysis plan.

Participants will be classified to their suitable statistical analysis sets according to the definition in the protocol. No patient can be excluded from the analysis, unless getting the permission of the meeting participants. All the meeting participants should sign the data locking consent, and the data auditing resolution.

8.5 Database Locking

The database will be locked if it fulfills all the aspects as followed:

•All the queries have been solved, and the database has been updated;

•No query has been found through the data inspection;

•The medical coding has been completed;

•The plan of the participants' classification has been approved;

•The final draft of the SAP has been made, and approved by the project leader.

The statisticians and the data administrators will sign the data locking form, and then the database will be locked. The locked database will be sent to the statisticians for further statistical analysis through the data format of SAS.

9. STATISTICAL CONSIDERATIONS

9.1 Study Populations

All patients with randomization will be included in the analysis set regardless of whether they receive any treatment. Most outcome measurements (except ~~*the ETT,*~~ micro-RNA and mRNA profiles) will be analyzed using full analysis sets and per-protocol sets according to intention-to-treat analysis. Safety analysis will be performed in a safety set, which is defined as a subset of subjects who were randomized and who received at least one treatment.

9.2 Statistical Analyses

The primary study hypothesis is that an initial usage of DHI added to optimal medical therapy could bring more clinical benefit for the patients with symptomatic stable angina than an initial strategy of optimal medical therapy alone. The primary analysis will be intention-to-treat with multiple imputations.The chi-square test will be used to compare the proportion of patients who have a clinically significant change in SAQ angina frequency at Day 30 from baseline (a post hoc analysis) between the two groups. Pre-specified subgroup analysis will also be performed according to the Statistical Analysis Plan (SAP).

The secondary efficacy and safety outcomes will be analyzed using the two sample t-tests, Wilcoxon rank-sum test, Chi-square test, Poisson regression or Fisher’s exact test according to the Statistical Analysis Plan (SAP).

All reported P values were two-sided; and a P value less than 0.05 was considered to be

statistically significant except that from the primary outcome analyses, in which Bonferroni's multiple comparison test was applied twice and P <0.016 was considered significant. All calculations were performed using SAS 9.4 (SAS Institute Inc, USA).

9.3 Statistical Analysis Plan (SAP)

Prior to database lock and before code breaking, a final version of the SAP shall be issued and approved by the study statistician, and the principal investigator. The SAP will define all "pre-specified, planned analyses" and provide the general specifications for the analysis of the data to be collected and presented in clinical information management systems.

10 ETHICAL ASPECTS

The investigator will ensure that this study is conducted in full conformance with the principles of the "Declaration of Helsinki" and applicable laws and regulations on the conduct of clinical trials.

This trial firstly will be approved by center institutional review board (IRB) in Chinese PLA General Hospital. For the other sites, if need, the trail also should be approved by the local IRB at the certain enrolling sites. Only when the trial protocol is approved by the IRB, the enrollment of participant will begin.

Informed consent is a process that is initiated prior to the individual's agreeing to participate in the study and continues throughout the individual's study participation. Extensive discussion of risks and possible benefits of this treatment will be provided to the subjects and their families. Consent forms describing in detail the study interventions/products, study procedures, and risks are given to the subject and written documentation of informed consent is required prior to starting intervention/administering study product. It must also be explained to the patients that they are completely free to refuse to enter the study or to withdraw from it at any time for any reason. A copy of

the informed consent document will be given to the subjects for their records.

REFERENCES

1. Lloyd-Jones, D. M. et al. Defining and setting national goals for cardio vascular health promotion and disease reduction: The American Heart Association's strategic impact goal through 2020 and beyond. Circulation 2010,121, 586-613

2. Chinese Society of Cardiology, Chinese Medical Association; Editorial Board, Chinese Journal of Cardiology. [Guideline for diagnosis and treatment of patients with chronic stable angina (no abstract)]. Zhonghua Xin Xue Guan Bing Za Zhi. 2007;35(3):195-206.

3. Gibbons, R. J. et al. Guidelines for the diagnosis and treatment of chronic stable angina. J Am Coll Cardiol 1999,133, 2092-2197

4. Wiest, F. C. et al. Suboptimal pharmacotherapeutic management of chronic stable angina in the primary care setting. Am J Med 2004,117, 234-241.

5. Kirwan, B. A., Lubsen, J. & Poole-Wilson, P. A. Treatment of angina pectoris: associations with symptom severity. Int J Cardiol 2005,98, 299-306

6. Spertus, J. A., Jones, P., McDonell, M., Fan, V. & Fihn, S. D. Health status predicts long-term outcome in outpatients with coronary disease. Circulation 2002,106, 43-49

7. Mozaffarian, D., Bryson, C. L., Spertus, J. A., McDonell, M. B. & Fihn, S. D. Anginal symptoms consistently predict total mortality among outpatients with coronary artery disease. Am Heart J 2003,146, 1015-1022.

8. Berecki-Gisolf, J., Humphreyes-Reid, L., Wilson, A. & Dobson, A. Angina symptoms are associated wityhh mortality in older women with ischemic heart disease. Circulation 2009,120,

2330-2336

9. Du J, Yang W, Yi D, Xie Y, Yang W, Zhuang Y, et al. Analysis of using Danhong injection to treatment coronary heart disease patients medicines based on real world HIS database. Zhongguo Zhong Yao Za Zhi. 2011;36:2821-2824.

10. Peng LH, Yu Z, Sheng CL. Dan Hong Injection for Angina Pectoris: A Systematic Review. Chin J Evid-based Med 2011, 11(1): 57-63

11. Weintraub WS, Spertus JA, Kolm P, Maron DJ, Zhang Z, Jurkovitz C, et al. Effect of PCI on quality of life in patients with stable coronary disease. N Engl J Med. 2008;359:677-87.

12. Gibbons RJ, Abrams J, Chatterjee K, Daley J, Deedwania PC, Douglas JS, et al. ACC/AHA 2002 guideline update for the management of patients with chronic stable angina-—summary article: a report of the American College of Cardiology/American Heart Association Task Force on Practice Guidelines (Committee on the Management of Patients With Chronic Stable Angina). Circulation. 2003;107:149-58.

13.Fox K, Garcia MA, Ardissino D, Buszman P, Camici PG, Crea F, et al.Guidelines on the management of stable angina pectoris: executive summary: the Task Force on the Management of Stable Angina Pectoris of the European Society of Cardiology. Eur Heart J. 2006;27:1341-81.

14. Campeau L. Grading of angina pectoris. Circulation 1976;54:522–3.

15. Zheng X. Guidelines of Clinical Research of New Drugs of Traditional Chinese Medicine. Beijing: China Medical Science Press; 2002.

SUMMARY OF CHANGES

All changes in final version of the protocol appear in blue and italic type. Major changes include the following:

1. In Participating Sites and Investigator, 6 sites and corresponding investigators initially took part in this trail and at last 31 sites and corresponding investigators all over China have participated in our study.

2. After the first interim analysis, the DMC recommend that the ETT test be omitted for better patient compliance and higher feasibility of the trial, as many participants were reluctant to complete the test due to the uncomfortable experience during ETT in China. This makes the changes on 6.3 Other Pre-specified efficacy outcomes and 6.5 Study Schema.

3. After the second interim analysis, the sample size is expanded to 920. The detailed information about the process of this change could be see in 3.2.2 Sample size re-estimation.

Danhong Injection for the treatment of chronic stable angina (Phase IV): an adaptive-design, randomized, multi-center, double-blind, placebo-controlled clinical trial

(Danhong Phase IV, DH20120703)

Statistical Analysis Plan
The Original Version: July 3, 2012

Prepared for and approved by the Executive committee of the study:

Prof. Yong-Yan Wang (Chair), China Academy of Chinese Medical Sciences

Prof. Xiao-Xi Du (Member), State Food and Drug Administration

Prof. Bo-Li Zhang (Member), China Academy of Chinese Medical Sciences

Prof. Run-Lin Gao (Member), Fuwai Hospital Chinese Academy of Medical Sciences

Prof. Wei-Liang Weng (Member), Xi-yuan Hospital Affiliated to China Academy of Chinese Medical Sciences

Dr. Ai-Ping Lv (Member), 1. Institute of Basic Research in Clinical Medicine, China Academy of Chinese Medical Sciences; 2. School of Chinese Medicine, Hong Kong Baptist University

Prof. Ming-Hui Yang (Member), Chinese PLA General Hospital

Prepared for Data Monitoring Committee

Prof. Chun-Ti Shen (Chair), Changzhou TCM Hospital Affiliated to Nanjing University of Chinese Medicine

Prof. Qi-Guang Chen (Member, independent statistician), Southeast University

Prof. Hua-Qiang Zhang (Member), Jiangsu Provincial Commission of Health and Family Planning

Dr. Hong-Wu Wang (Member), Tianjin University of Traditional Chinese Medicine

Dr. Xiao-Chun Sun (Member), Beijing Kelunrui Pharmaceutical Technology Co. Ltd.

Prof. Cheng-Shu Wang (Member), Zhongda Hospital Southeast University

Dr. Hai-Xia Dang (Member), China Academy of Chinese Medical Sciences

Dr. Jun Liu (Member), Institute of Basic Research in Clinical Medicine, China Academy of Chinese Medical Sciences

Prepared by an Independent Statistician:

Dr. Bing-Wei Chen (Statistician), Southeast University

Table of Contents

Table of Contents - 81 -
1. Introduction - 83 -
2. Study Objective - 84 -
3. Design - 84 -
3.1 Overview - 84 -
3.2 Eligibility criteria - 84 -
3.2.1 Diagnostic criteria - 84 -
3.2.2 Inclusion Criteria - 85 -
3.2.3 Exclusion Criteria - 86 -
3.3 Efficacy and Safety Outcomes - 87 -
3.3.1 Primary Efficacy Outcome - 87 -
3.3.2 Secondary Efficacy Outcomes - 87 -
3.3.3 Other Pre-specified Efficacy Outcomes - 88 -
3.3.4 Safety Outcomes - 88 -
3.4 Sample Size Calculation - 88 -
3.5 Study Schema - 89 -
4. Data Monitoring and Interim Analyses - 90 -
4.1 Data Management - 90 -
4.2 Establishment of Three Committees for DHI Phase IV Trials - 90 -
4.3 Interim Analyses - 90 -
5. Statistical Considerations - 91 -
5.1 Study Hypothesis - 91 -
5.2 Study Populations - 91 -
5.3 Methods of Statistical Analyses - 91 -
5.3.1 The general principle - 91 -
5.3.2 Statistical Comparisons Between Groups - 91 -
5.3.3 Center Effects for the Multicenter study - 92 -
5.3.4 The Management of Missing data - 92 -
5.3.5 Analysis Software - 92 -
5.4 Statistical Analyses - 93 -
5.4.1 Distribution of subjects - 93 -
5.4.2 Demographics and Baseline Characteristics - 93 -
5.4.3 Compliance and concomitant medication - 93 -
5.4.4 Analyses for Primary Outcome - 93 -
5.4.5 Analyses for Secondary Outcomes - 94 -

5.4.6 Other Pre-specified Efficacy Outcomes.. - 95 -

5.4.7 Safety Analyses.. - 96 -

5.4.8 Interim Analyses...- 96 -

References.. - 97 -

1. Introduction

Stable ischemic heart disease is a major cause of death worldwide, and nearly 58% of patients with coronary artery disease were suffering from chronic stable angina[1]. Current treatment options including pharmacotherapy (as organic nitrates, β-blockers, calcium channel antagonists), revascularization, lifestyle management and several alternative procedures, aims to reduce the risk of mortality and morbid events and to reduce symptoms[2]. For patients, it is often the latter that is of greater concern[3]. Despite these multiple treatment options[2], a high proportion of patients with stable angina remains symptomatic and their quality of life is impaired[4,5]. Moreover, several observational studies have shown that angina symptoms such as physical limitation and angina frequency are predictive indicators of mortality and acute coronary syndrome (ACS) hospitalizations[6-8]. Therefore, we need more therapies for patients with chronic angina in order to prevent the episode of the angina for the improvement of their quality of life.

Danhong injection (DHI), which is extracted from Danshen (Radix Salviae miltiorrhizae) and Honghua (Flos carthami), is widely used to treat the coronary heart disease. DHI, the profile of which has been analyzed using HPLC, is approved as a Chinese medicinal product for the treatment of various ischemic diseases by the State Food and Drug Administration of China (SFDA) in 2002. Some clinical studies have demonstrated that Danhong injection might be an effective and safe treatment option for the management of coronary heart disease[9,10.] However, the methodological quality of most previous studies was assessed to be, in general, low[10]. No critically appraised evidence, such as a well-designed randomized controlled trial, is available to provide a high level of evidence to justify the clinical use and recommendation of DHI. In this study, we will conduct a randomized, multicentre, double-blind,

placebo-controlled trial to investigate the efficacy and safety of DHI in patients with chronic stable angina.

2. Study Objective

The objective of this study is mainly to evaluate the effect of DHI in preventing the episode of chronic stable angina and improving the angina-specific quality of life, and the safety of DHI.

3. Design

3.1 Overview

This study is a three-stage adaptive-design, randomized, multicentre, double-blind, placebo-controlled trial. The number of subjects is initially estimated a total of 870 which 582 subjects are randomly assigned to in DHI group and 288 subjects in the control group. Both groups receive optimal medical therapy as recent guideline advocated. Subjects in the DHI group are treated by DHI (40 ml, qd) plus 0.9% normal saline (250 ml, ivgtt, qd), while subjects in the control group are treated by DHI placebo (0.9% normal saline, 40ml, qd) plus 0.9% normal saline (250 ml, ivgtt, qd).

3.2 Eligibility criteria

Eligible subjects are those who meet all of the following inclusion criteria and who do not have any of the listed exclusion criterion.

3.2.1 Diagnostic criteria

(1). The diagnostic criteria for chronic stable angina are determined according to the Chinese Guidelines for the Diagnosis and Treatment of Chronic Stable Angina (2007)[2], the ACC/AHA

Guideline Update for the Management of Patients With Chronic Stable Angina (2002)[11], and the European Society of Cardiology Guidelines for the Management of Stable Angina Pectoris[12].

(2). Traditional Chinese medicine diagnostic criteria are determined according to the Guidelines for Clinical Research of New Drugs of Traditional Chinese Medicine (2002)[13].

3.2.2 Inclusion Criteria

(1). Female or male inpatients.

(2). Age: 18–70 years.

(3). Patients with a clinical diagnosis of chronic stable angina. Patients must fulfill one of the following conditions:

(a) a history of myocardial infarction and ST-T changes;

(b) stenosis of more than 50 % in at least one major epicardial coronary artery, as shown by coronary angiography or computed tomography Angiography;

(c) coronary heart disease confirmed by radionuclide angiocardiography.

(4).Patients with a clinical diagnosis of *"Xueyu Zheng"* (blood stasis syndrome), which is defined as a score of at least 15 on the Chinese Medicine Symptom Scale of *"Xueyu Zheng"* for angina patients. The Chinese Medicine Symptom Scale of *"Xueyu Zheng"* includes the following items: (a) chest pain (0–10); (b) chest distress (0–10); (c) palpitation (0–5); (d) purple or dark lips (0–5); (e) purple or dark tongue (0–5); and (f) unsmooth pulse (0–5).

(5). Patients with moderate angina pectoris, which is defined as Grade II or III on the Canadian Cardiovascular Society Angina Grading Scale.

(6). Patient is willing to voluntarily participate and to sign a written informed consent document.

3.2.3 Exclusion Criteria

(1). Women who are pregnant, lactating, having a positive pregnancy test, or having a menstrual period at baseline.

(2). Women with childbearing potential disagree with using contraception during the treatment period.

(3). Patients with severe complications that would complicate the condition, as assessed by the investigator, including liver or renal dysfunction, severe cardiopulmonary dysfunction, pulmonary hypertension, chronic obstructive pulmonary disease, a history of epilepsy or cerebral haemorrhage.

(4). Patients were angina-free during the run-in period without taking any drug.

(5). Patients experienced myocardial infarction or who were classified as Grade IV on the Canadian Cardiovascular Society Angina Grading Scale within the preceding 3 months.

(6). Patients with chest pain that is caused by any other disease (e.g., acute myocardial infarction, severe neurosis, menopausal syndrome or hyperthyroidism).

(7). Patients with a history of drug-induced bleeding or a history of bleeding caused by warfarin.

(8). Patients with a history of haematopoietic disorder.

(9). Patients have had surgery within the previous 4 weeks or who have a haemorrhagic tendency.

(10). Patients who are participating in other trials or who have participated in other trials within the past 3 months.

(11). Patients with a history of allergy or with a known or suspected allergy to the study drug.

(12). Patients with a known or suspected history of alcohol or drug abuse within the past 2 years.

(13). Patients with a mental disorder.

(14). Patients who are unable to participate in the study, as judged by the investigator.

(15). Family members or relatives of the study centre staff.

3.3 Efficacy and Safety Outcomes

3.3.1 Primary Efficacy Outcome

The primary outcome is the proportion of patients who have a clinically significant change in Seattle Angina Questionnaire angina frequency (SAQAF) score at Day 30. Seattle Angina Questionnaire (SAQ) is a 19-item self-administered questionnaire that measures 5 domains of CAD-related health status: physical limitation(PL), angina stability(AS), angina frequency(AF), treatment satisfaction(TS), and disease perception/quality of life(DP). The total score ranges from 0 to 100; and higher scores indicate better health status.A clinically significant change in each scale of SAQ was defined as a difference of 8 points or more on the physical-limitation scale, 25 or more on the angina-stability scale, 20 or more on the angina-frequency scale, 12 or more on the treatment-satisfaction scale, and 16 or more on the quality-of-life scale, respectively.[14]

3.3.2 Secondary Efficacy Outcomes

(1).The total score of symptoms in a questionnaire related to traditional Chinese medicine;

(2). The proportion of patients who experience clinically significant changes in the other four Seattle Angina Questionnaire domains[14];

(3) The frequency of anginal attacks;

(4) Angina grade, according to the Canadian Cardiovascular Society (CCS) Angina Grading Scale;

(5) Consumption of short-acting nitrates;

(6) Changes in electrocardiogram results;

(7) Changes in serum lipid levels;

(8) Changes in high-sensitivity C-reactive protein levels;

(9) Changes in the platelet aggregation rate.

3.3.3 Other Pre-specified Efficacy Outcomes

(1) Changes in total exercise duration (TED) during exercise tolerance testing (ETT) from baseline to Day 14 in 290 patients selected after first interim analysis;

(2) Changes in time to 1mm st-segment depression during exercise tolerance testing (ETT) from baseline to Day 14 in 290 patients selected after first interim analysis;

(3) Changes in the micro-RNA and mRNA profiles of 60 patients selected at the Chinese PLA General Hospital and Xuanwu Hospital Capital Medical University.

3.3.4 Safety Outcomes

(1) Incidence of new-onset major vascular events within 90 days;

(2) Overall mortality within 90 days;

(3) Incidence of severe haemorrhages within 90 days;

(4) Incidence of moderate haemorrhages within 90 days;

(5) Incidence of adverse and serious adverse events.

3.4 Sample Size Calculation

Based on the previous study[14], the proportion of patients who had clinically significant change defined as at least 20-point improvement in Seattle Angina Questionnaire angina frequency (SAQAF) score was 30%, after standard conventional therapy for a month. In this trial, we apply an adaptive design for the statistical sample size calculation by software EAST5.2. It is hypothesized that an increase of at least 10% is of clinical significance for the DHI group; therefore, the number of subjects is initially

estimated to be 726 (one-sided test, α=0.05, β=0.15). To allow for a 20% dropout rate, a total of 870 patients will be recruited. As patients will be randomized into the DHI group or control group in a ratio of 2:1, the number of participants in the DHI group is 582 and in the control group is 288. According to the adaptive design, the sample size may be adjusted based on the results of two interim analyses, which will be carried out after one third (288) and two thirds (582) of patients have completed the trial, respectively.

3.5 Study Schema

Period	Run-in period	Treatment period			Follow-up period		
Day	-7	0	7	14	30	60	90
Informed consent	×	×					
Inclusion/exclusion criteria	×	×					
Medical history	×	×					
Medical examination	×						
Combined disease treatment	×	×	×	×	×	×	×
Outcomes							
SAQ		×			×	×	×
Symptoms questionnaire of TCM		×	×	×	×	×	×
Frequency of anginal attack per week	×	×	×	×	×	×	×
CCS grade		×	×	×	×	×	×
Consumption of short-acting nitrates	×	×	×	×	×	×	×
ECG		×	×	×	×	×	×
Serum lipids		×		×			
Hs-CRP		×		×			
Platelet aggregation rate		×		×			
New-onset major vascular events							×
Overall mortality							×
Incidence of severe hemorrhages							×
Incidence of moderate hemorrhages							×
AEs and SAEs			×	×	×	×	×
ETT (if necessary after 1st interim analysis)		×		×			
Profiles of micro-RNA in 60 patients in certain centers		×		×			×
Profiles of mRNA in 60 patients in certain centers		×		×			×

4. Data Monitoring and Interim Analyses

4.1 Data Management

(1) All data will be recorded by trained clinical investigators using standardized electronic case report forms and Brightech clinical information management systems (Brightech, Somerset, NJ, USA).

(2) The accuracy and reliability of the data will be ensured by the study monitor, who will verify and cross-check the electronic case report forms against the investigator's records (source document verification), using the maintenance of a drug dispensing log by the investigator.

(3) Front-end checks in the electronic case report forms and back-end checks in the clinical information management system will be used as a validation check of the data.

(4) If there are any discrepancies in the electronic case report forms, the results will be sent to the investigator for resolution.

(5) The results of the analysis must not be released with individual identification of the subjects until the database is closed.

4.2 Establishment of Three Committees for DHI Phase IV Trials

To ensure the effectiveness and integrity of the trial design, three committees have been established by the multicentre trial coordination group: the clinical trial guidance committee (Executive committee), the data monitoring committee (DMC) and the outcome evaluation committee. The first committee is responsible for the study design and the implementation process. The data monitoring committee will supervise the integrity and accuracy of data collection to control its quality. The outcome evaluation committee will evaluate key outcomes (including outcome measurements and adverse events) based on their clinical expertise.

4.3 Interim Analyses

Two interim analyses are prospectively planned, and these will be performed in a blinded manner after one-third (288) and two-thirds (592) of the patients, respectively. The statistical results of the interim analyses will be relayed to the data monitoring committee, who will decide on the re-estimation of

sample size and determine whether any subsequent modifications must be made to the trial protocol.

5. Statistical Considerations

5.1 Study Hypothesis

The primary study hypothesis is that an initial usage of DHI added to optimal medical therapy could bring more clinical benefit for the patients with symptomatic stable angina than an initial strategy of optimal medical therapy alone.

5.2 Study Populations

All patients with randomization will be included in the analysis set regardless of whether they receive any treatment. Most outcome measurements (except the ETT, micro-RNA and mRNA profiles) will be analyzed using full analysis sets and per-protocol sets according to intention-to-treat analysis. Safety analysis will be performed in a safety set, which is defined as a subset of subjects who were randomized and who received at least one treatment.

5.3 Methods of Statistical Analyses

5.3.1 The general principle

The statistical analysis will include the distribution of subjects, baseline characteristics of participants, compliance and concomitant medication, efficacy analysis and safety analysis.

For continuous variables, means and standard deviations will be presented, unless the variable has a skewed distribution, in which case medians, 25th and 75th percentiles will be presented.

For categorical variables, the number and percentage of participants within each category will be presented. For each variable (continuous or categorical), the number of missing values will be reported.

5.3.2 Statistical Comparisons Between Groups

Quantitative data will be compared using two-sample analysis of variance (ANOVA) and t tests. Paired t tests will be used to analyze differences between pre- and posttreatment time points. Two-sample t tests will be employed for comparisons between treatment groups. After ANOVA, we will then use

Student-Newman-Keuls significance tests for pairwise comparisons. Enumeration data will be analyzed using χ^2 tests, Cochran-Mantel-Haenszel χ^2 tests, Fisher's exact tests or Wilcoxon rank tests; χ^2 tests will be used for categorical data and rank sum tests will be used for ordinal data. All hypothesis testing will be carried out at the 5% (2-sided) significance level.

5.3.3 Center Effects for the Multicenter study

To estimate the overall variability of the center effects, we used the random center effects (RCE) accounting for center effects. Therefore, mixed-effect model was used for the primary outcome.

5.3.4 The Management of Missing data

Regardless of any violations, compliance or early withdrawal from the trial, if the patient is randomized, her data will be analyzed in our primary outcome analyses. For this we will use the SAS procedure Proc MI process. Each method will give rise to 100 different imputed data sets. We will fit our final model described before to each of these imputed datasets and then compute an overall estimate of the intervention effect as an average of the imputation specific estimates. The standard error of the overall intervention effect estimate will be calculated using Rubin's formula. SAS procedure Proc MIANALYZE will be used to implement these tasks.

To examine sensitivity to the MAR assumptions about the missing data, we will perform a sensitivity analysis under the missing not at random (MNAR) assumption.

5.3.5 Analysis Software

For all statistical analyses, SAS 9.4 software will be used.

5.4 Statistical Analyses

5.4.1 Distribution of subjects

Analytical statistics will be calculated to estimate the difference in the number of participants who have completed or who have been withdrawn from the trial between groups.

5.4.2 Demographics and Baseline Characteristics

Baseline characteristics in each group will be analyzed using descriptive statistics, including means or medians for continuous variables and percentages for categorical variables.

5.4.3 Compliance and concomitant medication

Compliance analysis will be based on full analysis sets, and analysis of concomitant medications will be based on safety sets.

5.4.4 Analyses for Primary Outcome

The chi-square test will be used to compare the proportion of patients who have a clinically significant change in SAQ angina frequency at Day 30 from baseline (a post hoc analysis) between the two groups.

Subgroup Analyses for the Primary Outcome

The primary outcome with respect to health status for prespecified subgroups according to age, sex, CCS angina grade in the baseline(II vs. III), heart rate in the baseline, disease duration, previous or no previous usage of optimal medical therapy, usage or no usage of nitroglycerin, presence or absence of diabetes, presence or absence of hypertension, presence or absence of hyperlipidemia will be analyzed.

Sensitivity Analyses for the Primary Outcome

Because the MAR assumption cannot be verified using the data, the sensitivity of inferences to departures from the MAR assumption should be tested.[15] A straightforward sensitivity analysis for the MAR assumption in multiple imputation is based on the pattern-mixture or control-based pattern imputation model under the MNAR assumption by using the SAS procedure Proc MI process. Therefore, mixed-effect model under MNAR assumption will be used.

5.4.5 Analyses for Secondary Outcomes

(1). The mean total score of symptoms in a questionnaire of *Xueyu Zheng* will be analyzed with the use of two sample t-tests comparing the scores of patients in the DHI and the control groups at each visit.

(2). The chi-square test will be used to compare the proportions of patients with syndrome improvement in *Xueyue Zheng* between the two groups. Significant syndrome improvement is defined as at least 30% reduction in the *Xueyue Zheng* score[13].

(3) The chi-square test will be used to compare the proportions of patients in each treatment group who were angina-free as defined by the angina frequency score on the SAQ (with a score of 100 indicating that the patient was angina-free).

(4) The chi-square test will be used to compare the proportion of patients who have a clinically significant change in the other four SAQ domains (physical limitation, angina stability, treatment satisfaction, and disease perception/quality of life) at Day 30 from baseline (a post hoc analysis).

(5) Mean observed data for each SAQ domain will be analyzed with the use of two sample t-tests comparing the scores of patients in the DHI and the control groups at each visit (a prespecified end point).

(6) The cumulative incidence density of angina frequency will be analyzed with the use of Poisson regression between the DHI and the control groups at each visit.

(7) The proportion of patients who have the improvement of the Canadian Cardiovascular Society (CCS) Angina Grading Scale, which is defined that the CCS grade change to Grade I or decrease 1 grade, will be analyzed with the use of the rank sum tests between the DHI and the control groups at each visit.

(8) The cumulative consumption density of nitroglycerin according to the diary will be analyzed with the use of Poisson regression between the two groups at each visit.

(9) The chi-square test will be used to compare the proportion of patients with normal ECG recordings between the two groups at each visit. The "normal ECG" in our trial is defined as including (a) normal sinus rhythm (each P wave is followed by a QRS and P wave rate 60 - 100 bpm with <10% variation); (b) normal P waves (height < 2.5 mm in lead II and width < 0.11 s in lead II); (c) normal PR interval (0.12 to 0.20 s); (d) normal QRS complex (< 0.12 s duration, no pathological Q waves and no evidence of left or right ventricular hypertrophy); (e) normal QT interval (0.42 s); (f) normal ST segment without any elevation or depression; (g) normal T wave; and (h) normal U wave.

(10) Serum lipid (total cholesterol, LDL-cholesterol, HDL-cholesterol, triglyceride) levels at Day 14 will be analyzed with the use of two sample t-tests between the two groups.

(11) The high-sensitivity C-reactive protein level at Day 14 will be analyzed with the use of two sample t-tests between the two groups.

(12) The platelet aggregation rate at Day 14 will be analyzed with the use of two sample t-tests between the two groups.

5.4.6 Other Pre-specified Efficacy Outcomes

(1) Changes in total exercise duration (TED) during exercise tolerance testing (ETT). from baseline to

Day 14 will be analyzed with the use of t-tests between the two groups.

(2) Changes in time to 1mm st-segment depression during exercise tolerance testing (ETT) from baseline to Day 14 will be analyzed with the use of t-tests between the two groups.

(3) Changes in the micro-RNA and mRNA profiles will be analyzed by Beijing Genomics Institute (BGI), which is not included in this statistical analysis plan.

5.4.7 Safety Analyses

(1) Incidence of new-onset major vascular events within 90 days will be analyzed with the chi-square test or Fisher's exact test between the two groups.

(2) Overall mortality within 90 days will be analyzed with the Chi-square test or Fisher's exact test between the two groups.

(3) Incidence of severe haemorrhages within 90 days will be analyzed with the Chi-square test or Fisher's exact test between the two groups.

(4) Incidence of moderate haemorrhages within 90 days will be analyzed with the Chi-square test or Fisher's exact test between the two groups.

(5) All adverse events and serious adverse events will be listed. Chi-square test or Fisher's exact test will be used to compare the incidence of adverse events between the two groups. P-value will not be corrected for multiple tests.

5.4.8 Interim Analyses

Two interim analyses will be performed in a blinded manner after one-third (288) and two-thirds (592) of the patients, respectively. The statistical results of the interim analyses will be performed by independent statistician in the data monitoring committee. Because the two interim analyses may lead

to an increased possibility of a type I error, Lan-DeMets alpha spending function with an O'Brien-Fleming boundary will be applied to adjust the results.

References

1. Lloyd-Jones, D. M. et al. Defining and setting national goals for cardio vascular health promotion and disease reduction: The American Heart Association's strategic impact goal through 2020 and beyond. Circulation 2010,121, 586-613

2. Chinese Society of Cardiology, Chinese Medical Association; Editorial Board, Chinese Journal of Cardiology. [Guideline for diagnosis and treatment of patients with chronic stable angina (no abstract)]. Zhonghua Xin Xue Guan Bing Za Zhi. 2007;35(3):195-206.

3. Gibbons, R. J. et al. Guidelines for the diagnosis and treatment of chronic stable angina. J Am Coll Cardiol 1999,133, 2092-2197

4. Wiest, F. C. et al. Suboptimal pharmacotherapeutic management of chronic stable angina in the primary care setting. Am J Med 2004,117, 234-241.

5. Kirwan, B. A., Lubsen, J. & Poole-Wilson, P. A. Treatment of angina pectoris: associations with symptom severity. Int J Cardiol 2005,98, 299-306

6. Spertus, J. A., Jones, P., McDonell, M., Fan, V. & Fihn, S. D. Health status predicts long-term outcome in outpatients with coronary disease. Circulation 2002,106, 43-49

7. Mozaffarian, D., Bryson, C. L., Spertus, J. A., McDonell, M. B. & Fihn, S. D. Anginal symptoms consistently predict total mortality among outpatients with coronary artery disease. Am Heart J 2003,146, 1015-1022.

8. Berecki-Gisolf, J., Humphreyes-Reid, L., Wilson, A. & Dobson, A. Angina symptoms are associated wityhh mortality in older women with ischemic heart disease. Circulation 2009,120, 2330-2336

9. Du J, Yang W, Yi D, Xie Y, Yang W, Zhuang Y, et al. Analysis of using Danhong injection to treatment coronary heart disease patients medicines based on real world HIS database. Zhongguo Zhong Yao Za Zhi. 2011;36:2821-2824.

10. Peng LH, Yu Z, Sheng CL. Dan Hong Injection for Angina Pectoris: A Systematic Review. Chin J Evid-based Med 2011, 11(1): 57-63

11.Gibbons RJ, Abrams J, Chatterjee K, Daley J, Deedwania PC, Douglas JS, et al. ACC/AHA 2002 guideline update for the management of patients with chronic stable angina-—summary article: a report of the American College of Cardiology/American Heart Association Task Force on Practice Guidelines (Committee on the Management of Patients With Chronic Stable Angina). Circulation. 2003;107:149-58.

12.Fox K, Garcia MA, Ardissino D, Buszman P, Camici PG, Crea F, et al.Guidelines on the management of stable angina pectoris: executive summary: the Task Force on the Management of Stable Angina Pectoris of the European Society of Cardiology. Eur Heart J. 2006;27:1341-81.

13.Zheng X. Guidelines of Clinical Research of New Drugs of Traditional Chinese Medicine. Beijing: China Medical Science Press; 2002.

14.Weintraub WS, Spertus JA, Kolm P, Maron DJ, Zhang Z, Jurkovitz C, et al. Effect of PCI on quality of life in patients with stable coronary disease. N Engl J Med. 2008;359:677-87.

15. Little RJ, D'Agostino R, Cohen ML, et al. The prevention and treatment of missing data in clinical trials. N Engl J Med. 2012. 367(14): 1355-60.

Danhong Injection for the treatment of chronic stable angina (Phase IV): an adaptive-design, randomized, multi-center, double-blind, placebo-controlled clinical trial

(Danhong Phase IV, DH20120703)

Statistical Analysis Plan

The Final Version: November 20, 2015

Prepared for and approved by the Executive committee of the study:

Prof. Yong-Yan Wang (Chair), China Academy of Chinese Medical Sciences

Prof. Xiao-Xi Du (Member), China Food and Drug Administration

Prof. Bo-Li Zhang (Member), China Academy of Chinese Medical Sciences

Prof. Run-Lin Gao (Member), Fuwai Hospital Chinese Academy of Medical Sciences

Prof. Wei-Liang Weng (Member), Xi-yuan Hospital Affiliated to China Academy of Chinese Medical Sciences

Dr. Ai-Ping Lv (Member), 1. Institute of Basic Research in Clinical Medicine, China Academy of Chinese Medical Sciences; 2. School of Chinese Medicine, Hong Kong Baptist University

Prof. Ming-Hui Yang (Member), Chinese PLA General Hospital

Prepared for Data Monitoring Committee

Prof. Chun-Ti Shen (Chair), Changzhou TCM Hospital Affiliated to Nanjing University of Chinese

Medicine

Prof. Qi-Guang Chen (Member, independent statistician), Southeast University

Prof. Hua-Qiang Zhang (Member), Jiangsu Provincial Commission of Health and Family Planning

Dr. Hong-Wu Wang (Member), Tianjin University of Traditional Chinese Medicine

Dr. Xiao-Chun Sun (Member), Beijing Kelunrui Pharmaceutical Technology Co. Ltd.

Prof. Cheng-Shu Wang (Member), Zhongda Hospital Southeast University

Dr. Hai-Xia Dang (Member), China Academy of Chinese Medical Sciences

Dr. Jun Liu (Member), Institute of Basic Research in Clinical Medicine, China Academy of Chinese Medical Sciences

Prepared by an Independent Statistician:

Dr. Bing-Wei Chen (Statistician), Southeast University

SUMMARY OF CHANGES

All changes in this version appear in blue. Major changes include the following:

1) After the first interim analysis, the DMC recommend that the ETT test be omitted for better patient compliance and higher feasibility of the trial, as many participants were reluctant to complete the test due to the uncomfortable experience during ETT in China. This makes the changes on 3.3.3 Other Pre-specified Efficacy Outcomes, 3.5 Study Schema and 5.4.6 Other Pre-specified Efficacy Outcomes.
2) After the second interim analysis, the sample size is expanded to 920. The detailed information about the process of this change could be see in 3.4 Sample Size Calculation.
3) After the second interim analysis, we added the details for the subgroup anlysis in the age, heart rate in the baseline, disease duration, see "Subgroup Analyses for the Primary Outcome " in 5.4.4 Analyses for Primary Outcome
4) After the second interim analysis, we added the "Interim Analyses Results" in 5.4.8 Interim Analyses.

Table of Contents

Table of Contents.......- 81 -
1. Introduction....... - 83 -
2. Study Objective....... - 84 -
3. Design.......- 84 -
3.1 Overview.......- 84 -
3.2 Eligibility criteria....... - 84 -
3.2.1 Diagnostic criteria.......- 84 -
3.2.2 Inclusion Criteria....... - 85 -
3.2.3 Exclusion Criteria....... - 86 -
3.3 Efficacy and Safety Outcomes.......- 87 -
3.3.1 Primary Efficacy Outcome....... - 87 -
3.3.2 Secondary Efficacy Outcomes.......- 87 -
3.3.3 Other Pre-specified Efficacy Outcomes....... - 88 -
3.3.4 Safety Outcomes.......- 88 -
3.4 Sample Size Calculation....... - 88 -
3.5 Study Schema.......- 89 -
4. Data Monitoring and Interim Analyses....... - 90 -
4.1 Data Management....... - 90 -
4.2 Establishment of Three Committees for DHI Phase IV Trials.......- 90 -
4.3 Interim Analyses....... - 90 -
5. Statistical Considerations....... - 91 -
5.1 Study Hypothesis....... - 91 -
5.2 Study Populations....... - 91 -
5.3 Methods of Statistical Analyses....... - 91 -
5.3.1 The general principle.......- 91 -
5.3.2 Statistical Comparisons Between Groups.......- 91 -
5.3.3 Center Effects for the Multicenter study....... - 92 -
5.3.4 The Management of Missing data....... - 92 -
5.3.5 Analysis Software.......- 92 -
5.4 Statistical Analyses....... - 93 -
5.4.1 Distribution of subjects.......- 93 -
5.4.2 Demographics and Baseline Characteristics.......- 93 -
5.4.3 Compliance and concomitant medication.......- 93 -
5.4.4 Analyses for Primary Outcome....... - 93 -
5.4.5 Analyses for Secondary Outcomes.......- 94 -

5.4.6 Other Pre-specified Efficacy Outcomes.......... - 95 -
5.4.7 Safety Analyses.......... - 96 -
5.4.8 Interim Analyses.......... - 96 -
References.......... - 97 -

1. Introduction

Stable ischemic heart disease is a major cause of death worldwide, and nearly 58% of patients with coronary artery disease were suffering from chronic stable angina[1]. Current treatment options including pharmacotherapy (as organic nitrates, β-blockers, calcium channel antagonists), revascularization, lifestyle management and several alternative procedures, aims to reduce the risk of mortality and morbid events and to reduce symptoms[2]. For patients, it is often the latter that is of greater concern[3]. Despite these multiple treatment options[2], a high proportion of patients with stable angina remains symptomatic and their quality of life is impaired[4,5]. Moreover, several observational studies have shown that angina symptoms such as physical limitation and angina frequency are predictive indicators of mortality and acute coronary syndrome (ACS) hospitalizations[6-8]. Therefore, we need more therapies for patients with chronic angina in order to prevent the episode of the angina for the improvement of their quality of life.

Danhong injection (DHI), which is extracted from Danshen (Radix Salviae miltiorrhizae) and Honghua (Flos carthami), is widely used to treat the coronary heart disease. DHI, the profile of which has been analyzed using HPLC, is approved as a Chinese medicinal product for the treatment of various ischemic diseases by the State Food and Drug Administration of China (SFDA) in 2002. Some clinical studies have demonstrated that Danhong injection might be an effective and safe treatment option for the management of coronary heart disease[9,10.] However, the methodological quality of most previous studies was assessed to be, in general, low[10]. No critically appraised evidence, such as a well-designed randomized controlled trial, is available to provide a high level of evidence to justify the clinical use and recommendation of DHI. In this study, we will conduct a randomized, multicentre, double-blind,

placebo-controlled trial to investigate the efficacy and safety of DHI in patients with chronic stable angina.

2. Study Objective

The objective of this study is mainly to evaluate the effect of DHI in preventing the episode of chronic stable angina and improving the angina-specific quality of life, and the safety of DHI.

3. Design

3.1 Overview

This study is a three-stage adaptive-design, randomized, multicentre, double-blind, placebo-controlled trial. The number of subjects is initially estimated a total of 870 which 582 subjects are randomly assigned to in DHI group and 288 subjects in the control group. Both groups receive optimal medical therapy as recent guideline advocated. Subjects in the DHI group are treated by DHI (40 ml, qd) plus 0.9% normal saline (250 ml, ivgtt, qd), while subjects in the control group are treated by DHI placebo (0.9% normal saline, 40ml, qd) plus 0.9% normal saline (250 ml, ivgtt, qd).

3.2 Eligibility criteria

Eligible subjects are those who meet all of the following inclusion criteria and who do not have any of the listed exclusion criterion.

3.2.1 Diagnostic criteria

(1). The diagnostic criteria for chronic stable angina are determined according to the Chinese Guidelines for the Diagnosis and Treatment of Chronic Stable Angina (2007)[2], the ACC/AHA

Guideline Update for the Management of Patients With Chronic Stable Angina (2002)[11], and the European Society of Cardiology Guidelines for the Management of Stable Angina Pectoris[12].

(2). Traditional Chinese medicine diagnostic criteria are determined according to the Guidelines for Clinical Research of New Drugs of Traditional Chinese Medicine (2002)[13].

3.2.2 Inclusion Criteria

(1). Female or male inpatients.

(2). Age: 18–70 years.

(3). Patients with a clinical diagnosis of chronic stable angina. Patients must fulfill one of the following conditions:

(a) a history of myocardial infarction and ST-T changes;

(b) stenosis of more than 50 % in at least one major epicardial coronary artery, as shown by coronary angiography or computed tomography Angiography;

(c) coronary heart disease confirmed by radionuclide angiocardiography.

(4).Patients with a clinical diagnosis of *"Xueyu Zheng"* (blood stasis syndrome), which is defined as a score of at least 15 on the Chinese Medicine Symptom Scale of *"Xueyu Zheng"* for angina patients. The Chinese Medicine Symptom Scale of *"Xueyu Zheng"* includes the following items: (a) chest pain (0–10); (b) chest distress (0–10); (c) palpitation (0–5); (d) purple or dark lips (0–5); (e) purple or dark tongue (0–5); and (f) unsmooth pulse (0–5).

(5). Patients with moderate angina pectoris, which is defined as Grade II or III on the Canadian Cardiovascular Society Angina Grading Scale.

(6). Patient is willing to voluntarily participate and to sign a written informed consent document.

3.2.3 Exclusion Criteria

(1). Women who are pregnant, lactating, having a positive pregnancy test, or having a menstrual period at baseline.

(2). Women with childbearing potential disagree with using contraception during the treatment period.

(3). Patients with severe complications that would complicate the condition, as assessed by the investigator, including liver or renal dysfunction, severe cardiopulmonary dysfunction, pulmonary hypertension, chronic obstructive pulmonary disease, a history of epilepsy or cerebral haemorrhage.

(4). Patients were angina-free during the run-in period without taking any drug.

(5). Patients experienced myocardial infarction or who were classified as Grade IV on the Canadian Cardiovascular Society Angina Grading Scale within the preceding 3 months.

(6). Patients with chest pain that is caused by any other disease (e.g., acute myocardial infarction, severe neurosis, menopausal syndrome or hyperthyroidism).

(7). Patients with a history of drug-induced bleeding or a history of bleeding caused by warfarin.

(8). Patients with a history of haematopoietic disorder.

(9). Patients have had surgery within the previous 4 weeks or who have a haemorrhagic tendency.

(10). Patients who are participating in other trials or who have participated in other trials within the past 3 months.

(11). Patients with a history of allergy or with a known or suspected allergy to the study drug.

(12). Patients with a known or suspected history of alcohol or drug abuse within the past 2 years.

(13). Patients with a mental disorder.

(14). Patients who are unable to participate in the study, as judged by the investigator.

(15). Family members or relatives of the study centre staff.

3.3 Efficacy and Safety Outcomes

3.3.1 Primary Efficacy Outcome

The primary outcome is the proportion of patients who have a clinically significant change in Seattle Angina Questionnaire angina frequency (SAQAF) score at Day 30. Seattle Angina Questionnaire (SAQ) is a 19-item self-administered questionnaire that measures 5 domains of CAD-related health status: physical limitation(PL), angina stability(AS), angina frequency(AF), treatment satisfaction(TS), and disease perception/quality of life(DP). The total score ranges from 0 to 100; and higher scores indicate better health status.A clinically significant change in each scale of SAQ was defined as a difference of 8 points or more on the physical-limitation scale, 25 or more on the angina-stability scale, 20 or more on the angina-frequency scale, 12 or more on the treatment-satisfaction scale, and 16 or more on the quality-of-life scale, respectively.[14]

3.3.2 Secondary Efficacy Outcomes

(1).The total score of symptoms in a questionnaire related to traditional Chinese medicine;

(2). The proportion of patients who experience clinically significant changes in the other four Seattle Angina Questionnaire domains[14];

(3) The frequency of anginal attacks;

(4) Angina grade, according to the Canadian Cardiovascular Society (CCS) Angina Grading Scale;

(5) Consumption of short-acting nitrates;

(6) Changes in electrocardiogram results;

(7) Changes in serum lipid levels;

(8) Changes in high-sensitivity C-reactive protein levels;

(9) Changes in the platelet aggregation rate.

3.3.3 Other Pre-specified Efficacy Outcomes

~~(1) Changes in total exercise duration (TED) during exercise tolerance testing (ETT) from baseline to Day 14 in 290 patients selected after first interim analysis;~~

~~(2) Changes in time to 1mm st-segment depression during exercise tolerance testing (ETT) from baseline to Day 14 in 290 patients selected after first interim analysis;~~

~~(3)~~ Changes in the micro-RNA and mRNA profiles of 60 patients selected at the Chinese PLA General Hospital and Xuanwu Hospital Capital Medical University.

3.3.4 Safety Outcomes

(1) Incidence of new-onset major vascular events within 90 days;

(2) Overall mortality within 90 days;

(3) Incidence of severe haemorrhages within 90 days;

(4) Incidence of moderate haemorrhages within 90 days;

(5) Incidence of adverse and serious adverse events.

3.4 Sample Size Calculation

Based on the previous study[14], the proportion of patients who had clinically significant change defined as at least 20-point improvement in Seattle Angina Questionnaire angina frequency (SAQAF) score was 30%, after standard conventional therapy for a month. In this trial, we apply an adaptive design for the statistical sample size calculation by software EAST5.2. It is hypothesized that an increase of at least 10% is of clinical significance for the DHI group; therefore, the number of subjects is initially

estimated to be 726 (one-sided test, α=0.05, β=0.15). To allow for a 20% dropout rate, a total of 870 patients will be recruited. As patients will be randomized into the DHI group or control group in a ratio of 2:1, the number of participants in the DHI group is 582 and in the control group is 288. According to the adaptive design, the sample size may be adjusted based on the results of two interim analyses, which will be carried out after one third (288) and two thirds (582) of patients have completed the trial, respectively.

Sample size re-estimation

In the first interim analysis, 288 patients were enrolled in the trial and 275 had the primary outcome. In the group A, 105 patients (57.07%, 105/183) had a clinically significant change in SAQAF at Day 30, while in the group B, 40 (43.96%, 40/91) got the clinically significant change in SAQAF (Z=2.063), and thus, the difference was not statistically significant between the two groups (Lan-DeMets spending function boundaries not be crossed). Conditional power (CP) was calculated as 0.984 using EAST 5.2, and thus, DMC decided that the sample size should not be re-estimated.

In the second interim analysis, 576 patients were enrolled in the trial and 550 had the primary outcome. In the group A, 205 patients (56.32%, 205/364) had a clinically significant change in SAQAF at Day 30, while in the group B, 78 (41.94%,78/186) got the clinically significant change in SAQAF (Z=3.228), and the difference was statistically significant between the two groups(Lan-DeMets spending function boundary was crossed). Indeed, the trial could be stopped in advance because of the good efficacy of DHI according to the suggest of EAST 5.2. However, since there was no significant difference in the proportion of patients who have a clinically significant change in SAQAF at Day 90 between the two groups (64.64% vs. 55.91%, P= 0.0513), DMC determined to continue the trial and re-estimate the total sample size as 920 using the above data at Day 90 with approximate 10%

drop-out.

3.5 Study Schema

3.5.1 Initial study schema

Period	Run-in period	Treatment period			Follow-up period		
Day	-7	0	7	14	30	60	90
Informed consent	×	×					
Inclusion/exclusion criteria	×	×					
Medical history	×	×					
Medical examination	×						
Combined disease treatment	×	×	×	×	×	×	×
Outcomes							
SAQ		×			×	×	×
Symptoms questionnaire of TCM		×	×	×	×	×	×
Frequency of anginal attack per week	×	×	×	×	×	×	×
CCS grade		×	×	×	×	×	×
Consumption of short-acting nitrates	×	×	×	×	×	×	×
ECG		×	×	×	×	×	×
Serum lipids		×		×			
Hs-CRP		×		×			
Platelet aggregation rate		×		×			
New-onset major vascular events							×
Overall mortality							×
Incidence of severe hemorrhages							×
Incidence of moderate hemorrhages							×
AEs and SAEs			×	×	×	×	×
ETT (if necessary after 1st interim analysis)		×		×			
Profiles of micro-RNA in 60 patients in certain centers		×		×			×
Profiles of mRNA in 60 patients in certain centers		×		×			×

3.5.2Study schema changed after the first interim analysis

Period	Run-in period	Treatment period			Follow-up period		
Day	-7	0	7	14	30	60	90
Informed consent	×	×					
Inclusion/exclusion criteria	×	×					
Medical history	×	×					
Medical examination	×						
Combined disease treatment	×	×	×	×	×	×	×
Outcomes							

SAQ		×			×	×	×
Symptoms questionnaire of TCM		×	×	×	×	×	×
Frequency of anginal attack per week	×	×	×	×	×	×	×
CCS grade		×	×	×	×	×	×
Consumption of short-acting nitrates	×	×	×	×	×	×	×
ECG		×	×	×	×	×	×
Serum lipids		×		×			
Hs-CRP		×		×			
Platelet aggregation rate		×		×			
New-onset major vascular events							×
Overall mortality							×
Incidence of severe hemorrhages							×
Incidence of moderate hemorrhages							×
AEs and SAEs			×	×	×	×	×
Profiles of micro-RNA in 60 patients in certain centers		×		×			×
Profiles of mRNA in 60 patients in certain centers		×		×			×

4. Data Monitoring and Interim Analyses

4.1 Data Management

(1) All data will be recorded by trained clinical investigators using standardized electronic case report forms and Brightech clinical information management systems (Brightech, Somerset, NJ, USA).

(2) The accuracy and reliability of the data will be ensured by the study monitor, who will verify and cross-check the electronic case report forms against the investigator’s records (source document verification), using the maintenance of a drug dispensing log by the investigator.

(3) Front-end checks in the electronic case report forms and back-end checks in the

clinical information management system will be used as a validation check of the data.

(6) If there are any discrepancies in the electronic case report forms, the results will be sent to the investigator for resolution.

(7) The results of the analysis must not be released with individual identification of the subjects until the database is closed.

4.2 Establishment of Three Committees for DHI Phase IV Trials

To ensure the effectiveness and integrity of the trial design, three committees have been established by the multicentre trial coordination group: the clinical trial guidance committee (Executive committee), the data monitoring committee (DMC) and the outcome evaluation committee. The first committee is responsible for the study design and the implementation process. The data monitoring committee will supervise the integrity and accuracy of data collection to control its quality. The outcome evaluation committee will evaluate key outcomes (including outcome measurements and adverse events) based on their clinical expertise.

4.3 Interim Analyses

Two interim analyses are prospectively planned, and these will be performed in a blinded manner after one-third (288) and two-thirds (592) of the patients, respectively. The statistical results of the interim analyses will be relayed to the data monitoring committee, who will decide on the re-estimation of sample size and determine whether any subsequent modifications must be made to the trial protocol.

5. Statistical Considerations

5.1 Study Hypothesis

The primary study hypothesis is that an initial usage of DHI added to optimal medical therapy could bring more clinical benefit for the patients with symptomatic stable angina than an initial strategy of optimal medical therapy alone.

5.2 Study Populations

All patients with randomization will be included in the analysis set regardless of whether they receive any treatment. Most outcome measurements (except the ETT, micro-RNA and mRNA profiles) will be analyzed using full analysis sets and per-protocol sets according to intention-to-treat analysis. Safety analysis will be performed in a safety set, which is defined as a subset of subjects who were randomized and who received at least one treatment.

5.3 Methods of Statistical Analyses

5.3.1 The general principle

The statistical analysis will include the distribution of subjects, baseline characteristics of participants, compliance and concomitant medication, efficacy analysis and safety analysis.

For continuous variables, means and standard deviations will be presented, unless the variable has a skewed distribution, in which case medians, 25th and 75th percentiles will be presented.

For categorical variables, the number and percentage of participants within each category will be presented. For each variable (continuous or categorical), the number of missing values will be reported.

5.3.2 Statistical Comparisons Between Groups

Quantitative data will be compared using two-sample analysis of variance (ANOVA) and t tests. Paired t tests will be used to analyze differences between pre- and posttreatment time points. Two-sample t tests will be employed for comparisons between treatment groups. After ANOVA, we will then use Student-Newman-Keuls significance tests for pairwise comparisons. Enumeration data will be analyzed using χ^2 tests, Cochran-Mantel-Haenszel χ^2 tests, Fisher's exact tests or Wilcoxon rank tests; χ^2 tests will be used for categorical data and rank sum tests will be used for ordinal data. All hypothesis testing will be carried out at the 5% (2-sided) significance level.

5.3.3 Center Effects for the Multicenter study

To estimate the overall variability of the center effects, we used the random center effects (RCE) accounting for center effects. Therefore, mixed-effect model was used for the primary outcome.

5.3.4 The Management of Missing data

Regardless of any violations, compliance or early withdrawal from the trial, if the patient is randomized, her data will be analyzed in our primary outcome analyses. For this we will use the SAS procedure Proc MI process. Each method will give rise to 100 different imputed data sets. We will fit our final

model described before to each of these imputed datasets and then compute an overall estimate of the intervention effect as an average of the imputation specific estimates. The standard error of the overall intervention effect estimate will be calculated using Rubin's formula. SAS procedure Proc MIANALYZE will be used to implement these tasks.

To examine sensitivity to the MAR assumptions about the missing data, we will perform a sensitivity analysis under the missing not at random (MNAR) assumption.

5.3.5 Analysis Software

For all statistical analyses, SAS 9.4 software will be used.

5.4 Statistical Analyses

5.4.1 Distribution of subjects

Analytical statistics will be calculated to estimate the difference in the number of participants who have completed or who have been withdrawn from the trial between groups.

5.4.2 Demographics and Baseline Characteristics

Baseline characteristics in each group will be analyzed using descriptive statistics, including means or medians for continuous variables and percentages for categorical variables.

5.4.3 Compliance and concomitant medication

Compliance analysis will be based on full analysis sets, and analysis of concomitant medications will be based on safety sets.

5.4.4 Analyses for Primary Outcome

The chi-square test will be used to compare the proportion of patients who have a clinically significant change in SAQ angina frequency at Day 30 from baseline (a post hoc analysis) between the two groups.

Subgroup Analyses for the Primary Outcome

The primary outcome with respect to health status for prespecified subgroups according to age (<65 years vs. ≥65 years), sex, CCS angina grade in the baseline(II vs. III), heart rate in the baseline (<75 beats/min vs. ≥75 beats/min), disease duration(≤15 days vs. >15 days), previous or no previous usage of optimal medical therapy, usage or no usage of nitroglycerin, presence or absence of diabetes, presence or absence of hypertension, presence or absence of hyperlipidemia will be analyzed.

Sensitivity Analyses for the Primary Outcome

Because the MAR assumption cannot be verified using the data, the sensitivity of inferences to departures from the MAR assumption should be tested.[15] A straightforward sensitivity analysis for the MAR assumption in multiple imputation is based on the pattern-mixture or control-based pattern imputation model under the MNAR assumption by using the SAS procedure Proc MI process. Therefore, mixed-effect model under MNAR assumption will be used.

5.4.5 Analyses for Secondary Outcomes

(1). The mean total score of symptoms in a questionnaire of *Xueyu Zheng* will be analyzed with the use of two sample t-tests comparing the scores of patients in the DHI and the control groups at each visit.

(2). The chi-square test will be used to compare the proportions of patients with syndrome improvement in *Xueyue Zheng* between the two groups. Significant syndrome improvement is defined

as at least 30% reduction in the *Xueyue Zheng* score[13].

(3) The chi-square test will be used to compare the proportions of patients in each treatment group who were angina-free as defined by the angina frequency score on the SAQ (with a score of 100 indicating that the patient was angina-free).

(4) The chi-square test will be used to compare the proportion of patients who have a clinically significant change in the other four SAQ domains (physical limitation, angina stability, treatment satisfaction, and disease perception/quality of life) at Day 30 from baseline (a post hoc analysis).

(5) Mean observed data for each SAQ domain will be analyzed with the use of two sample t-tests comparing the scores of patients in the DHI and the control groups at each visit (a prespecified end point).

(6) The cumulative incidence density of angina frequency will be analyzed with the use of Poisson regression between the DHI and the control groups at each visit.

(7) The proportion of patients who have the improvement of the Canadian Cardiovascular Society (CCS) Angina Grading Scale, which is defined that the CCS grade change to Grade I or decrease 1 grade, will be analyzed with the use of the rank sum tests between the DHI and the control groups at each visit.

(8) The cumulative consumption density of nitroglycerin according to the diary will be analyzed with the use of Poisson regression between the two groups at each visit.

(9) The chi-square test will be used to compare the proportion of patients with normal ECG recordings between the two groups at each visit. The "normal ECG" in our trial is defined as including (a) normal sinus rhythm (each P wave is followed by a QRS and P wave rate 60 - 100 bpm with <10% variation); (b) normal P waves (height < 2.5 mm in lead II and width < 0.11 s in lead II); (c) normal PR interval

(0.12 to 0.20 s); (d) normal QRS complex (< 0.12 s duration, no pathological Q waves and no evidence of left or right ventricular hypertrophy); (e) normal QT interval (0.42 s); (f) normal ST segment without any elevation or depression; (g) normal T wave; and (h) normal U wave.

(10) Serum lipid (total cholesterol, LDL-cholesterol, HDL-cholesterol, triglyceride) levels at Day 14 will be analyzed with the use of two sample t-tests between the two groups.

(11) The high-sensitivity C-reactive protein and/or C-reactive protein level at Day 14 will be analyzed with the use of two sample t-tests between the two groups.

(12) The platelet aggregation rate at Day 14 will be analyzed with the use of two sample t-tests between the two groups.

5.4.6 Other Pre-specified Efficacy Outcomes

~~(1) Changes in total exercise duration (TED) during exercise tolerance testing (ETT). from baseline to Day 14 will be analyzed with the use of t-tests between the two groups.~~

~~(2) Changes in time to 1mm st-segment depression during exercise tolerance testing (ETT) from baseline to Day 14 will be analyzed with the use of t-tests between the two groups.~~

~~(3)~~ Changes in the micro-RNA and mRNA profiles will be analyzed by Beijing Genomics Institute (BGI), which is not included in this statistical analysis plan.

5.4.7 Safety Analyses

(1) Incidence of new-onset major vascular events within 90 days will be analyzed with the chi-square test or Fisher's exact test between the two groups.

(2) Overall mortality within 90 days will be analyzed with the Chi-square test or Fisher's exact test between the two groups.

(3) Incidence of severe haemorrhages within 90 days will be analyzed with the Chi-square test or Fisher's exact test between the two groups.

(4) Incidence of moderate haemorrhages within 90 days will be analyzed with the Chi-square test or Fisher's exact test between the two groups.

(5) All adverse events and serious adverse events will be listed. Chi-square test or Fisher's exact test will be used to compare the incidence of adverse events between the two groups. P-value will not be corrected for multiple tests.

5.4.8 Interim Analyses

Two interim analyses will be performed in a blinded manner after one-third (288) and two-thirds (592) of the patients, respectively. The statistical results of the interim analyses will be performed by independent statistician in the data monitoring committee. Because the two interim analyses may lead to an increased possibility of a type I error, Lan-DeMets alpha spending function with an O'Brien-Fleming boundary will be applied to adjust the results.

Interim Analyses Results

(1) After the first interim analysis, the DMC recommended that the ETT test be omitted for better patient compliance and higher feasibility of the trial, as many participants were reluctant to complete the test due to the uncomfortable experience during ETT in China.

(2) The sample size was re-estimated to 920 by the DMC based on the results of the second interim analysis.

References

1. Lloyd-Jones, D. M. et al. Defining and setting national goals for cardio vascular health promotion

and disease reduction: The American Heart Association's strategic impact goal through 2020 and beyond. Circulation 2010,121, 586-613

2. Chinese Society of Cardiology, Chinese Medical Association; Editorial Board, Chinese Journal of Cardiology. [Guideline for diagnosis and treatment of patients with chronic stable angina (no abstract)]. Zhonghua Xin Xue Guan Bing Za Zhi. 2007;35(3):195-206.

3. Gibbons, R. J. et al. Guidelines for the diagnosis and treatment of chronic stable angina. J Am Coll Cardiol 1999,133, 2092-2197

4. Wiest, F. C. et al. Suboptimal pharmacotherapeutic management of chronic stable angina in the primary care setting. Am J Med 2004,117, 234-241.

5. Kirwan, B. A., Lubsen, J. & Poole-Wilson, P. A. Treatment of angina pectoris: associations with symptom severity. Int J Cardiol 2005,98, 299-306

6. Spertus, J. A., Jones, P., McDonell, M., Fan, V. & Fihn, S. D. Health status predicts long-term outcome in outpatients with coronary disease. Circulation 2002,106, 43-49

7. Mozaffarian, D., Bryson, C. L., Spertus, J. A., McDonell, M. B. & Fihn, S. D. Anginal symptoms consistently predict total mortality among outpatients with coronary artery disease. Am Heart J 2003,146, 1015-1022.

8. Berecki-Gisolf, J., Humphreyes-Reid, L., Wilson, A. & Dobson, A. Angina symptoms are associated wityhh mortality in older women with ischemic heart disease. Circulation 2009,120, 2330-2336

9. Du J, Yang W, Yi D, Xie Y, Yang W, Zhuang Y, et al. Analysis of using Danhong injection to treatment coronary heart disease patients medicines based on real world HIS database. Zhongguo Zhong Yao Za Zhi. 2011;36:2821-2824.

10. Peng LH, Yu Z, Sheng CL. Dan Hong Injection for Angina Pectoris: A Systematic Review. Chin J

Evid-based Med 2011, 11(1): 57-63

11.Gibbons RJ, Abrams J, Chatterjee K, Daley J, Deedwania PC, Douglas JS, et al. ACC/AHA 2002 guideline update for the management of patients with chronic stable angina-—summary article: a report of the American College of Cardiology/American Heart Association Task Force on Practice Guidelines (Committee on the Management of Patients With Chronic Stable Angina). Circulation. 2003;107:149-58.

12.Fox K, Garcia MA, Ardissino D, Buszman P, Camici PG, Crea F, et al.Guidelines on the management of stable angina pectoris: executive summary: the Task Force on the Management of Stable Angina Pectoris of the European Society of Cardiology. Eur Heart J. 2006;27:1341-81.

13.Zheng X. Guidelines of Clinical Research of New Drugs of Traditional Chinese Medicine. Beijing: China Medical Science Press; 2002.

14.Weintraub WS, Spertus JA, Kolm P, Maron DJ, Zhang Z, Jurkovitz C, et al. Effect of PCI on quality of life in patients with stable coronary disease. N Engl J Med. 2008;359:677-87.

15. Little RJ, D'Agostino R, Cohen ML, et al. The prevention and treatment of missing data in clinical trials. N Engl J Med. 2012. 367(14): 1355-60.

SUMMARY OF CHANGES

All changes in the final version appear in blue. Major changes include the following:

1. After the first interim analysis, the DMC recommend that the ETT test be omitted for better patient compliance and higher feasibility of the trial, as many participants were reluctant to complete the test due to the uncomfortable experience during ETT in China. This makes the changes on 3.3.3 Other Pre-specified Efficacy Outcomes, 3.5 Study Schema and 5.4.6 Other Pre-specified Efficacy Outcomes.

2. After the second interim analysis, the sample size is expanded to 920. The detailed information about the process of this change could be see in 3.4 Sample Size Calculation.

3. After the second interim analysis, we added the details for the subgroup anlysis in the age, heart rate in the baseline, disease duration, see "Subgroup Analyses for the Primary Outcome " in 5.4.4 Analyses for Primary Outcome

4. After the second interim analysis, we added the "Interim Analyses Results" in 5.4.8 Interim Analyses.
