# Supplementary material for: Detection of an anti-angina therapeutic module in the effective population treated by a multi-target drug Danhong injection: a randomized trial
Source: Signal Transduct Target Ther. 2021 Sep 1;6:329. doi: 10.1038/s41392-021-00741-x (PMC8410855; doi:10.1038/s41392-021-00741-x)
Supplement: Supplementary file 2 — Supplemental Material [file 41392_2021_741_MOESM2_ESM.docx]

Supplementary Materials for

Detection of an anti-angina therapeutic module in the effective population treated by a multi-target drug Danhong injection: a randomized trial

Jun Liu^1#^, Dan-Dan Li^2#^, Wei Dong^2^, Yu-Qi Liu^2^, Yang Wu^3^, Da-Xuan Tang^3^, Fu-Chun Zhang^4^, Meng Qiu^4^, Qi Hua^5^, Jing-Yu He^5^, Jun Li^6^, Bai Du^6^, Ting-Hai Du^7^, Lin-Lin Niu^7^, Xue-Jun Jiang^8^, Bo Cui^8^, Jiang-Bin Chen^8^,Yang-Gan Wang^9^, Hai-Rong Wang^9^, Qin Yu^10^, Jing He^10^, Yi-Lin Mao^11^, Xiao-Fang Bing^11^, Yue Deng^12^, Yu-Dan Tian^12^, Qing-Hua Han^13^, Da-Jin Liu^13^, Li-Qin Duan^13^, Ming-Jun Zhao^14^, Cui-Ying Zhang^14^, Hai-Ying Dai^15^, Ze-Hua Li^15^, Ying Xiao^15^, You-Zhi Hu^16^, Xiao-Yu Huang^16^, Kun Xing^17^, Xin Jiang^17^, Chao-Feng Liu^18^, Jing An^18^, Feng-Chun Li^19^, Tao Tao^19^, Jin-Fa Jiang^20^, Ying Yang^20^, Yao-Rong Dong^21^, Lei Zhang^21^, Guang Fu^22^, Ying Li^22^, Shu-Wei Huang^23^, Li-Ping Dou^23^, Lan-Jun Sun^24^, Ying-Qiang Zhao^24^, Jie Li^24^, Yun Xia^25^, Jun Liu^25^, Fan Liu^26^, Wen-Jin He^26^, Ying Li^26^, Jian-Cong Tan^27^, Yang Lin^27^, Ya-Bin Zhou^28^, Jian-Fei Yang^28^, Guo-Qing Ma^29^, Hui-Jun Chen^29^, He-Ping Liu^30^, Zong-Wu Liu^30^, Jian-Xiong Liu^31^, Xiao-Jia Luo^31^, Xiao-Hong Bin^31^, Ya-Nan Yu^1^, Hai-Xia Dang^1,32^, Bing Li^1,33^, Fei Teng^34^, Wang-Min Qiao^34^, Xiao-Long Zhu^34^, Bing-Wei Chen^35^, Qi-Guang Chen^35^, Chun-Ti Shen^36^, Yong-Yan Wang^1*^, Yun-Dai Chen^2*^, Zhong Wang^1*^

Correspondence to: Zhong Wang (zhonw@vip.sina.com) or Yun-Dai Chen (cyundai@medmail.com.cn) or Yong-Yan Wang (wangyongyan2010@sina.cn)

**This PDF file includes:**

Materials and Methods

Figure. S1 The standard of the fingerprint electropherogram of Danhong Injection.

Figure. S2 The chemical structure of the 5 main component content in Danghong injection.

Figure. S3 Mean Scores on the Seattle Angina Questionnaire from Baseline to 90 Days.

Figure. S4 Bar graph of enriched terms across 104 DEG-mRNAs, colored by p-values.

Figure. S5 The distributions on the categories of the enriched GO biological processes (a) and KEGG pathways (b) from the targeted modular network of DHI

Figure. S6 The differentially expressed modules (DEMs) at Day 30 detected in the populations with different effect according to Z_summary_ compared with the control group and theirselve’s Day 0

Figure. S7 The corralation between theΔAF and the DEMs in the populations with a mild effect (40>ΔAF≥20) (a) and no effect (ΔAF<20) (b)

Table S1 Baseline characteristics of the study participants.

Table S2 The concomitant medications between two groups

Table S3 Patients with clinically significant improvement from baseline in scores on the Seattle Angina Questionnaire

Table S4 Three sensitivity analysis for the changes from baseline in SAQ-AF scale

Table S5 Freedom from angina over time as assessed with the angina-frequency scale of Seattle Angina Questionnaire (angina-frequency score of 100 on the Seattle Angina Questionnaire) according to treatment group.

Table S6 Scores on the Seattle Angina Questionnaire from baseline to 90 days

Table S7 Changes in Xueyu-Zheng score from baseline to 90 days

Table S8 Changes in angina frequency and the consumption of the nitroglycerin according to patients’ dairy

Table S9 Changes in CCS grade and ECG recordings

Table S10 Changes in plasma lipid level, C-reactive protein and platelet aggregation rate

Table S11 Adverse Events during the Study

Table S12 Clinical characteristics of patients who provided serum samples for RNA sequencing.

Table S13 The differentially expressed genes (mRNA and miRNA) before and after treatment with Danhong injection compared with control group

Table S14 The Z_summary_ value and the name of the 25 targeted pharmacological modules of Danhong injection versus placebo at Day 30

Table S15 The targeted modular map of 25 functional modules

Table S16 The enriched BP-GOs and KEGG pathways of the targeted modules of DHI

Table S17 The corralation between the 90 DEGs at Day 30 and the ΔAF

Table S18 The enrichment on the GO biological process for the effective therapeutic module

**Other Supplementary Materials for this manuscript include the following:**

Supplement File: Protocol&SAP

**Materials and Methods**

**Study design**

This study is a three-stage adaptive-design, randomized, multicentre, double-blind, placebo-controlled trial. The trial protocol ^1^ (File.S1) was approved by the central institutional review board from Chinese PLA General Hospital (IRB No.【2012】Pharmaceutical (025)). This trial complied with the principles of the Declaration of Helsinki. Informed written consent was obtained from all participants. Patients or the public were not involved in the design, or conduct, or reporting, or dissemination plans of our research.

**Participants**

**Inclusion criteria**

(1). Female or male inpatients.

(2). Age: 18–70 years.

(3). Patients with a clinical diagnosis of chronic stable angina.

(4). Patients with a clinical diagnosis of “Xueyu Zheng” (blood stasis syndrome).

(5). Patients with moderate angina pectoris, which is defined as Grade II or III on the Canadian Cardiovascular Society Angina Grading Scale.

(6). Patient is willing to voluntarily participate and to sign a written informed consent document.

**Exclusion criteria**

(1). Women who are pregnant, lactating, having a positive pregnancy test, or having a menstrual period at baseline.

(2). Women with childbearing potential disagree with using contraception during the treatment period.

(3). Patients with severe complications that would complicate the condition, as assessed by the investigator, including liver or renal dysfunction, severe cardiopulmonary dysfunction, pulmonary hypertension, chronic obstructive pulmonary disease, a history of epilepsy or cerebral haemorrhage.

(4). Patients were angina-free during the run-in period without taking any drug.

(5). Patients experienced myocardial infarction or who were classified as Grade IV on the Canadian Cardiovascular Society Angina Grading Scale within the preceding 3 months.

(6). Patients with chest pain that is caused by any other disease (e.g., acute myocardial infarction, severe neurosis, menopausal syndrome or hyperthyroidism).

(7). Patients with a history of drug-induced bleeding or a history of bleeding caused by warfarin.

(8). Patients with a history of haematopoietic disorder.

(9). Patients have had surgery within the previous 4 weeks or who have a haemorrhagic tendency.

(10). Patients who are participating in other trials or who have participated in other trials within the past 3 months.

(11). Patients with a history of allergy or with a known or suspected allergy to the study drug.

(12). Patients with a known or suspected history of alcohol or drug abuse within the past 2 years.

(13). Patients with a mental disorder.

(14). Patients who are unable to participate in the study, as judged by the investigator.

(15). Family members or relatives of the study centre staff.

**Interventions**

Optimal medical therapy was given to all participants throughout the trial, in strict accordance with the Chinese Guidelines for the Diagnosis and Treatment of Chronic Stable Angina (2007) ^2^.Optimal medical therapy includes: 1) Antiplatelet agents: aspirin (75-100 mg, once per day) or clopidogrel (if the patient is intolerant to aspirin). Patients with a history of percutaneous coronary intervention should be prescribed both of these agents.2) Lipid-lowering agents (statins): atorvastatin (10-20 mg, once per day) or simvastatin (20-40 mg, once per day).3) Anti-angina agents: β-blockers (metoprolol 50-200 mg, once per day, or analogous agents); long-acting nitrates (isosorbide mononitrate 40-60 mg, once per day); or calcium channel blockers (amlodipine 5-20 mg, once per day).4) Patients with diabetes and hypertension were advised to take angiotensin-converting enzyme inhibitors or angiotensin-receptor blockers (e.g., lisinopril 10-20 mg, once per day, or losartan 50 mg, once per day) as a secondary preventive measure for chronic stable angina. All of these basic treatments were recorded in detail in the patients’ medical records as well as in their e-CRFs. Participants who received optimal medical therapy for more than 1 week prior to study initiation were randomized directly into one of the groups; otherwise, a run-in period of 1 week of optimal medical therapy was performed before randomization.

Participants in the DHI and control groups received a daily intravenous drip of 40 ml DHI and 40 ml placebo (0.9% normal saline), respectively, added to 250 ml 0.9% normal saline. All participants underwent 2 weeks of treatment and 76 days of follow-up. Patients could take a nitroglycerin tablet (0.5 mg per tablet, provided by Beijing Yimin Pharmaceutical Co., Ltd.) in the event of an angina attack, and they recorded the details regarding the angina attack and the usage of nitroglycerin in a patient diary. Other drugs with the same indications as DHI were not allowed to be used throughout the study period. All concomitant medications were recorded on the eCRFs.

**Outcome measurements**

Angina-specific health status was assessed at baseline, Day 30, 60, and 90. Each assessment was performed with the use of the Seattle Angina Questionnaire (SAQ), a 19-item self-administered questionnaire that measures 5 domains of CAD-related health status: physical limitation(PL), angina frequency(AF), treatment satisfaction(TS), and disease perception/quality of life(DP). The total score ranges from 0 to 100; and higher scores indicate better health status.^3^ The primary outcome is the proportion of patients who have a clinically significant change in Seattle Angina Questionnaire angina frequency (SAQAF) score at Day 30. A clinically significant change in each scale of SAQ was defined as a difference of 8 points or more on the physical-limitation scale, 25 or more on the angina-stability scale, 20 or more on the angina-frequency scale, 12 or more on the treatment-satisfaction scale, and 16 or more on the quality-of-life scale, respectively. ^3^

The efficacy evaluation scale of Chinese Medicine Symptom in “*Xueyu Zheng*”, a 6-item questionnaire of the symptoms related to *Xueyu Zheng* including chest pain, chest distress, palpitation, purple or dark lips, purple or dark tongue and unsmooth pulse ^1^ , was used to assess the condition of patients with *Xueyu Zheng* at each visit (Day 0, Day 7, Day 14, Day 30, Day 60, Day 90). The mean total score of symptoms in a questionnaire of *Xueyu Zheng* was analyzed and compared between two groups at each visit. Moreover, the proportions of patients with syndrome improvement in *Xueyue Zheng* between the two groups were recorded at each visit. Significant syndrome improvement is defined as at least 30% reduction in the *Xueyue Zheng* score ^1^.

The patients were asked to record their angina attacks and the usage of short-acting nitrates in the diary. At each visit (Day 7, 14, 30, 60, 90), the investigator collected the diary and recorded them onto the electronic case report forms (e-CRFs).

At each visit, the angina grade of each patient was evaluated according to the Canadian Cardiovascular Society (CCS) Angina Grading Scale. The proportion of patients who have the improvement of the Canadian Cardiovascular Society (CCS) Angina Grading Scale was compared between two groups at each visit (Day 7, 14, 30, 60, 90), which was defined that the CCS grade change to Grade I or decrease 1 grade.

The 12-lead electrocardiogram (ECG) was tested at each visit (Day 0, 7, 14, 30, 60, 90). The result was evaluated by two independent ECG doctors. The proportion of patients with normal ECG recordings was compared between the two groups at each visit. The "normal ECG" in our trial was defined as including (1) normal sinus rhythm (each P wave is followed by a QRS and P wave rate 60 - 100 bpm with <10% variation); (2) normal P waves (height < 2.5 mm in lead II and width < 0.11 s in lead II); (3) normal PR interval (0.12 to 0.20 s); (4) normal QRS complex (< 0.12 s duration, no pathological Q waves and no evidence of left or right ventricular hypertrophy); (5) normal QT interval (0.42 s); (6) normal ST segment without any elevation or depression; (7) normal T wave; and (8) normal U wave.

The levels of serum lipid, high-sensitivity C-reactive protein (hs-CRP) or C-reactive protein (CRP) , platelet aggregation rate (PAR) were tested at baseline and Day 14. The changes in biochemical indexes such as serum lipids, hs-CRP or CRP and PAR at Day 14 were also considered as the secondary outcomes.

Another outcome measure, the change of total exercise duration (TED) in exercise tolerance test (ETT) from baseline to Day 14, was assessed in 290 patients until the first interim analysis. However, after the first interim analysis, the Data Monitoring Committee (DMC) recommended that the ETT test be omitted for better patient compliance and higher feasibility of the trial.

**Safety outcomes**

The safety outcomes include: (1) the incidence of new-onset major vascular events within 90 days. Major vascular events include death induced by cardiac or cerebral vascular disorders , myocardial infarction, stroke, transient ischemic attack (TIA), coronary intervention (including stent thrombosis), peripheral vascular intervention, hospitalization for unstable angina, and acute heart failure (HF) events; (2) Overall mortality within 90 days; (3) incidence of severe haemorrhages within 90 days. Severe haemorrhages includes fatal bleeding, primary intracranial hemorrhage, post-traumatic symptomatic intracranial hemorrhage, or bleeding requiring transfusion, infusion, use of vasoconstrictor drugs and surgical intervention; (4) incidence of moderate haemorrhages within 90 days. Moderate haemorrhages includes bleeding requiring infusion but do not reach severe bleeding, absolute reduction in hemoglobin/hematocrit, severe disability induced by bleeding, intraocular hemorrhage with severe visual field defects, etc; (5) incidence of adverse and serious adverse events.

**Randomization**

All eligible patients who consent to participation will be randomized into either the Danhong injection or the placebo group in a 2:1 ratio. Randomization will be conducted using a clinical information management system (Brightech, Somerset, USA). This system automatically randomizes patients and generates a randomization number with a message noting their assigned treatment. In addition, randomization will be stratified based on whether a patient received standard conventional therapy for more than 1 week prior to study initiation.

**Blinding**

Participants and research personnel will be blinded to Danhong injection therapy or placebo treatment group assignments until the study has concluded. Because the colour of Danhong injection and 0.9 % saline are different,the dropping bottles will be wrapped in sealed shaded bags, and brown infusion devices will be used for infusion. These procedures will be implemented by two professional nurses who will be required to sign a confidentiality agreement before study initiation and not to contact each other. One of the professional nurses will be in charge of preparing the drugs in a special transfusion room and sealing the infusion bottles with shaded brown bags. The other nurse will take the prepared drugs from the transfusion room to the infusion nurse and supervise the infusion process to ensure that the allocation of the drugs is blinded to the patients (the shaded brown bags will not be unwrapped during infusion and will be checked for integrity after infusion).

**RNA profile analytic platforms for differentially expressed genes (DEGs)**

The serum samples of patients from 2 selected centers (301 Hospital and Xuanwu Hospital) were sequenced using the Illumina HiSeq sequencing platform according to the manufacturer’s instructions and a previously described protocol ^4-5^. We evaluated the raw data on the two aspects: filtering contaminants and initial judgment of the data. Mapping to the human genome, miRBase ^6^ , Rfam and the NCBI database was carried out using Bowtie 2 ^7^ (one mismatch allowed). The prediction of novel miRNAs was performed using the popular software package miRDeep2 ^8^. The DEG results were obtained using DESeq2 ^9^, Student’s t-test was used to detect significantly differences in expression profiles before and after drug administration. Significant miRNAs and mRNAs were defined as having a p-value <0.05 and at least a 1.5-fold change. The enriched Gene Ontology biological processes (GO-BPs) and Kyoto Encyclopedia of Genes and Genomes (KEGG) pathways of the DEG-mRNAs were evaluated by Metascape ^10^, and the target mRNAs of the DEG-miRNAs were predicted via TargetSCAN Release 7.2 ^11^.

**Construction of the Targeted Modular Map of Danhong Injection (DHI)**

The construction of the gene coexpression construction for DHI at Day 30 and the identification of the corresponding modules were implemented using WGCNA, an R package for weighted correlation network analysis ^12^. To identify coexpression modules, we used a topological overlap measure to perform average linkage hierarchical clustering to obtain a dendrogram whose branches were identified using the dynamic hybrid tree cut algorithm ^13^. Then, the branches were defined as modules, and each module was assigned a color. We set the minimum module size to 3.

To quantitatively assess whether modules in the DHI group were changed in coexpression patterns independent of the control group, we adopted a Z_summary_ statistic implemented in the module preservation function of WGCNA ^12^. We defined a module with a Z_summary_ < 0 compared to the control group as a targeted differentially expressed module (DEM), which might be activated by a drug ^14^. The equation defining Z_summary_ is as follows ^15^ :

$\text{Z}_{\text{summary}}\text{=}\frac{\text{median(Z}_{\text{meanCor}}\text{,}_{\text{ZmeanAdj}}\text{,Z}_{\text{propvarExpl}}\text{,Z}_{\text{meamKME}}\text{)+median(Z}_{\text{cor.KIM}}\text{,Z}_{\text{cor.KME}}\text{,Z}_{\text{cor.cor}}\text{)}}{2}$ (1)

Furthermore, we reconstructed the targeted DEM network with the DEMs as nodes and the interactive relationships between modules defined by the connectivity score (CS) ^16^, as the edges:

$CS(i,j)\text{=}\frac{Number\_Of\_Links(Comm_{i},Comm_{j})}{Size(Comm_{i})*Size(Comm_{j})}$ (2)

This network was visualized using Cytoscape 3.6.0 ^17^. The enriched GO-BPs and KEGG pathways of the genes in each module were also identified using the Database for Annotation, Visualization and Integrated Discovery (DAVID) Bioinformatics Resources 6.8 ^18^, and we classified the GO terms and pathways based on the GO_slim2 ^19^ and KEGG functional hierarchies. Afterwards, we renamed each targeted DEM using its hub genes (the genes with the highest degree centrality) or the most significant GO-BPs and KEGG pathways; thus, each targeted DEM was assigned a certain function and designated a targeted functional module (TFM).

**Detection of the Effective Therapeutic Module of DHI**

Furthermore, to determine the RNA expression profile feature of the targeted population in order to detect the effective therapeutic module of DHI, we divided the populations in the DHI group at Day 30 into 3 groups according to ΔAF (defined as the change in SAQ-AF at Day 30 from baseline at Day 0): the group with the best effect (ΔAF≥40), a group with a mild effect (40>ΔAF≥20) and a group with no effect (ΔAF<20). Then, we constructed the gene coexpression networks and detected the DEMs for each population group according to Z_summary_ ^14^ using WGCNA ^12^ as described above. Afterwards, the correlation value (r) of each DEM with ΔAF was calculated by WGCNA, while the correlations between the DEGs at Day 30 and ΔAF were calculated using Pearson correlation coefficients. The differences in the correlation of DEGs with ΔAF and DEMs with ΔAF were calculated by an unpaired t-test. Significant DEMs were defined as those with r≥0.3 and p<0.05, and the most significant DEM (with the highest r-value and lowest p-value) in the population with the best effect was considered the most effective therapeutic module (METM) of DHI. We selected the METM genes expressed in 4 types of populations with different effects (ΔAF<0, ΔAF=10, ΔAF=20 and ΔAF≥40), reconstructed the corresponding gene coexpression modules using WGCNA ^12^ and visualized them using Cytoscape 3.6.0 ^17^. A topological analysis of these modules was performed with the network analysis functionality of Cytoscape 3.6.0 ^17^. In addition, we obtained a global topological parameter D score by integrating each normalized topology parameter in these modules calculated above to show the flexibility of the METM in different populations treated with DHI. The D score model was based on Euclidean distance, which calculates the distance (similarity) between two points, especially for measurements of similarity between multidimensional data (Euclidean n-space) ^20-21^. If a= (a_1_, a_2_, ..., a_n_) and b = (b_1_, b_2_, ..., b_n_) are two modules in Euclidean n-space, then the D score is the distance (d) from a to b and is defined as follows:

$\mathbf{d=}\sqrt{{\mathbf{（}\mathbf{a}_{\mathbf{1}}\mathbf{-}\mathbf{b}_{\mathbf{1}}\mathbf{）}}^{\mathbf{2}}\mathbf{+.......+}{\mathbf{（}\mathbf{a}_{\mathbf{n}}\mathbf{-}\mathbf{b}_{\mathbf{n}}\mathbf{）}}^{\mathbf{2}}}$

Furthermore, we calculated the correlation between ΔAF and the D score or the number of edges using linear regression. Moreover, the genes with high phenotype correlation (gene significance for AF ≥0.9) and high module membership (module membership≥0.9) were identified using WGCNA; these genes were considered the biomarkers of the anti-angina effect of DHI.

**Statistical Analysis**

According to the previous study ^2^, the proportion of patients who had clinically significant change defined as at least 20-point improvement in Seattle Angina Questionnaire angina frequency (SAQAF) score was 30%, after standard conventional therapy for a month. In this trial, we adopt an adaptive design by using the statistical sample size calculation software EAST5.2. It is hypothesized that an increase of at least 10% is of clinical significance for the DHI group; therefore, the number of subjects is initially estimated to be 726 (one-sided test, α=0.05, β=0.15). To allow for a 20% dropout rate, a total of 870 patients will be recruited. As patients will be randomized into the DHI group or control group in a ratio of 2:1, the number of participants in the DHI group is 582 and that in the control group is 288. Two interim analyses with O’Brien-Fleming ^22^ stopping boundaries were performed to re-estimate the sample size after 288 and 582 patients completed the trial.

In the first interim analysis, 288 patients were enrolled in the trial and 275 had the primary outcome. In the group A, 105 patients (57.07%, 105/183) had a clinically significant change in SAQAF at Day 30, while in the group B, 40 (43.96%, 40/91) got the clinically significant change in SAQAF (Z=2.063), and thus, the difference was not statistically significant between the two groups (Lan-DeMets spending function boundary not be crossed). Conditional power (CP) was calculated as 0.984 using EAST 5.2, and thus, DMC decided that the sample size should not be re-estimated.

In the second interim analysis, 576 patients were enrolled in the trial and 550 had the primary outcome. In the group A, 205 patients (56.32%, 205/364) had a clinically significant change in SAQAF at Day 30, while in the group B, 78 (41.94%,78/186) got the clinically significant change in SAQAF (Z=3.228), and the difference was statistically significant between the two groups (Lan-DeMets spending function boundary was crossed). Indeed, the trial could be stopped in advance because of the good efficacy of DHI according to the suggest of EAST 5.2. However, since there was no significant difference in the proportion of patients who have a clinically significant change in SAQAF at Day 90 between the two groups (64.64% vs. 55.91%, P= 0.0513), DMC determined to continue the trial and re-estimate the total sample size as 920 (one-sided test, α=0.05, β=0.1) using the above data at Day 90 with approximate 10% drop-out.

The primary outcome was analyzed according to the intention-to-treat principle. Missing data were handled by regression-based multiple imputation using the fully conditional method ^23^. Three sensitivity analyses (1 preplanned and two post hoc) were conducted for the primary outcome. The preplanned used control-based pattern model to evaluate sensitivity to missing data departure from the missing at random assumption. Two post hoc sensitivity analyses evaluated whether baseline imbalance in clinical site and a mixed-effect model with repeated measures could have diluted the estimates of treatment effect.Continuous variables were reported as the mean and standard deviation (SD), and categorical variables were reported as frequencies and percentages with 95% CIs. Statistical differences between groups were analyzed using Student’s t-test or the Wilcoxon rank-sum test for quantitative data and chi-squared test for categorical data. All reported P values were two-sided, and a P value less than 0.05 was considered to be statistically significant except in the primary outcome analyses, in which Bonferroni's multiple comparison test was applied twice and P <0.016 was considered significant. All calculations were performed using SAS 9.4 (SAS Institute Inc., USA).

**REFERENCES**

1. Wang, P.Q. et al. Danhong injection in the treatment of chronic stable angina: study protocol for a randomized controlled trial. *Trials.* **16**,474 (2015).
2. Chinese Society of Cardiology, Chinese Medical Association, & Editorial Board, Chinese Journal of Cardiology. [Guideline for diagnosis and treatment of patients with chronic stable angina (no abstract)]. *Zhonghua Xin Xue Guan Bing Za Zhi.* **35**, 195-206 (2007)
3. Weintraub, W.S. et al. Effect of PCI on quality of life in patients with stable coronary disease. *N. Engl. J. Med.* **359**, 677-687 (2008).
4. Meder, B. et al. Influence of the confounding factors age and sex on microRNA profiles from peripheral blood. *Clin. Chem.* **60**, 1200-1208 (2014).
5. Leidinger, P. et al. A blood based 12-miRNA signature of Alzheimer disease patients. *Genome. Biol.* **14**, R78 (2013).
6. Griffiths-Jones, S., Saini, H. K., van Dongen, S., & Enright, A. J. miRBase: tools for microRNA genomics. *Nucleic. Acids. Res.* **36(Database issue)**, D154-D158 (2008).
7. Langmead, B., & Salzberg, S. L. Fast gapped-read alignment with Bowtie 2. *Nat Methods.* **9**, 357-359 (2012).
8. Friedländer, M. R., Mackowiak, S. D., Li, N., Chen, W., & Rajewsky, N. miRDeep2 accurately identifies known and hundreds of novel microRNA genes in seven animal clades. *Nucleic, Acids, Res.* **40**, 37-52 (2012).
9. Love, M. I., Huber, W., & Anders, S. Moderated estimation of fold change and dispersion for RNA-seq data with DESeq2. *Genome Biol.* **15**, 550 (2014).
10. Zhou, Y. et al. Metascape provides a biologist-oriented resource for the analysis of systems-level datasets. *Nat. Commun.* **10,** 1523 (2019).
11. Agarwal, V., Bell, G. W., Nam, J. W., & Bartel, D. P. Predicting effective microRNA target sites in mammalian mRNAs. *Elife.* **4**, e05005 (2015).
12. Langfelder, P. & Horvath, S. WGCNA: an R package for weighted correlation network analysis. *BMC Bioinformatics.* **9**:559 (2008).
13. Langfelder, P., Zhang, B., & Horvath, S. Defining clusters from a hierarchical cluster tree: the Dynamic Tree Cut package for R. *Bioinformatics* **24**, 719-720 (2008).
14. Li, B. et al. Quantitative Identification of Compound-Dependent On-Modules and Differential Allosteric Modules From Homologous Ischemic Networks. *CPT. Pharmacometrics. Syst. Pharmacol.* **5**, 575-584 (2016).
15. Langfelder, P., Luo, R., Oldham, M. C., & Horvath, S. Is my network module preserved and reproducible? *PLoS Comput. Biol.* **7**, e1001057 (2011).
16. Levy, O., Knisbacher, B.A., Levanon, E.Y. & Havlin, S. Integrating networks and comparative genomics reveals retroelement proliferation dynamics in hominid genomes. *Sci. Adv.* **3**, e1701256 (2017).
17. Demchak, B., et al. Cytoscape: the network visualization tool for GenomeSpace workflows. *F1000Res* **3**, 151(2014).
18. Huang, d., Sherman, B. T., & Lempicki, R. A. Systematic and integrative analysis of large gene lists using DAVID Bioinformatics Resources. *Nat Protoc.* **4**, 44-57 (2009).
19. Hu, Z. L., Bao, J., & Reecy, M. J. CateGOrizer: A Web-Based Program to Batch Analyze Gene Ontology Classification Categories. *Online Journal of Bioinformatics.* **9**, 108-112(2008).
20. Wang, B. et al. Similarity network fusion for aggregating data types on a genomic scale. *Nat. Methods* **11,** 333-337 (2014).
21. Nguyen, V.A. & Lió, P. Measuring similarity between gene expression profiles: a Bayesian approach. *BMC. Genomics.* **10 Suppl 3**, S14 (2009).
22. O'Brien, P.C. & Fleming, T.R. A multiple testing procedure forclinical trials. *Biometrics* **35**, 549-556 (1979).
23. Little, R.J. et al. The prevention and treatment of missing data in clinical trials. *N. Engl. J. Med.* **367**, 1355-1360 (2012).

**Figure. S1.**


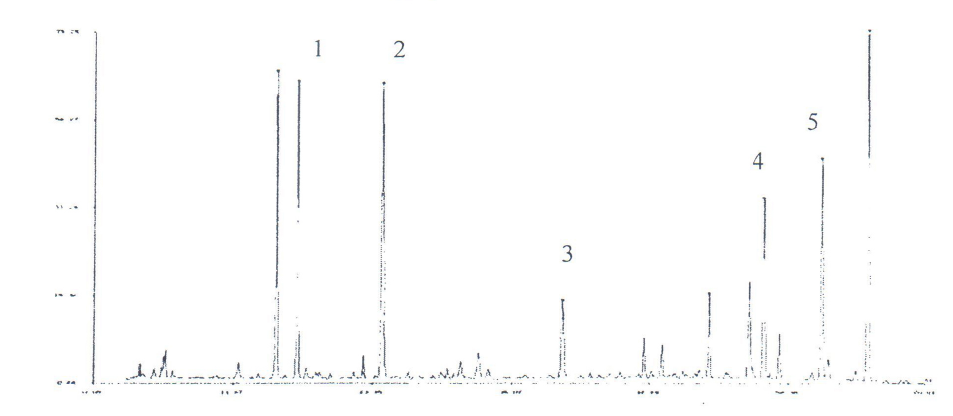


**Figure. S1. The standard of the fingerprint electropherogram of Danhong Injection.** The fingerprint electropherogram is obtained using Agilent 1100 Series High Performance Liquid Chromatograph (Agilent Chemstation Workstation) or Shimadzu LC-20AT High Performance Liquid Chromatograph (Shimadzu LC Solution Chromatographic Workstation) and Kromasil C18 chromatographic column (250mm×4.6mm,5μm). The main components in the injection includes: 1. Danshensu sodium; 2.Protocatechualdehyde; 3. p-coumaric acid; 4, Rosmarinci acid; 5. Salvianolic acid B. The fingerprint electropherogram of the test sample of Danhong injection should show the same peak value as thechromatographic peak retention time of the corresponding reference solution. According to the similarity evaluation system for fingerprint electropherogram of reference solution, the similarity should be more than 0.9 between the test sample (using in our trail) and the reference solution.

#

# **Figure. S2.**

#
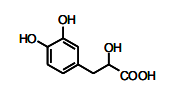

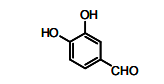

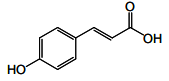


a.Danshensu sodium C_9_H_10_O_5_ b. Protocatechualdehyde C_7_H_6_O_3_  c. p-coumaric acid C_9_H_8_O_3_


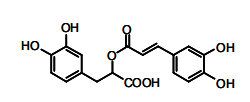

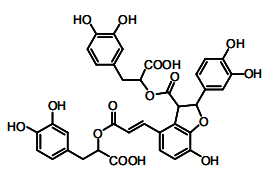


d. Rosmarinci acid C_18_H_16_O_8_  e. Salvianolic acid B C_36_H_30_O_16_

**Figure. S2. The chemical structure of the 5 main component content in Danghong injection.** This injection should contain Danshensu sodium not less than 0.80mg, protocatechualdehyde not less than 0.10mg, p-coumaric acid not less than 20μg, rosmarinci acid not less than 0.10mg, and salvianolic acid B not less than 0.16mg.

**Figure. S3.**

**
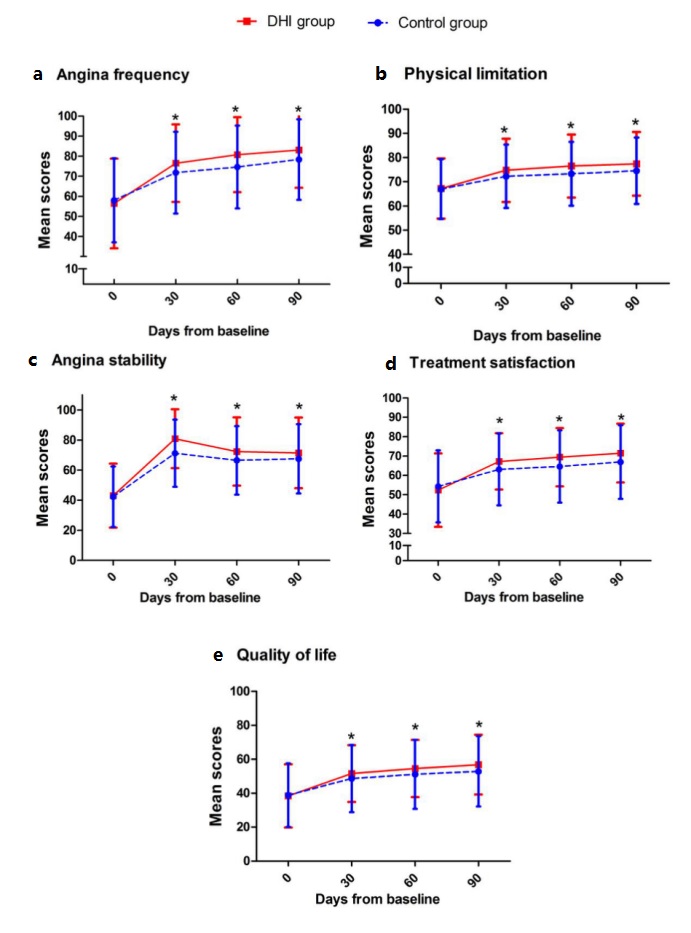
**

**Figure. S3. Mean Scores on the Seattle Angina Questionnaire from Baseline to 90 Days.**

**Figure. S4.**


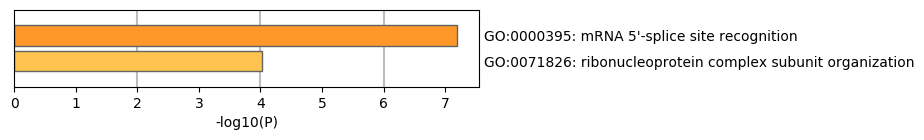


**Figure. S4. Bar graph of enriched terms across 104 DEG-mRNAs, colored by p-values.**

**Figure. S5.**


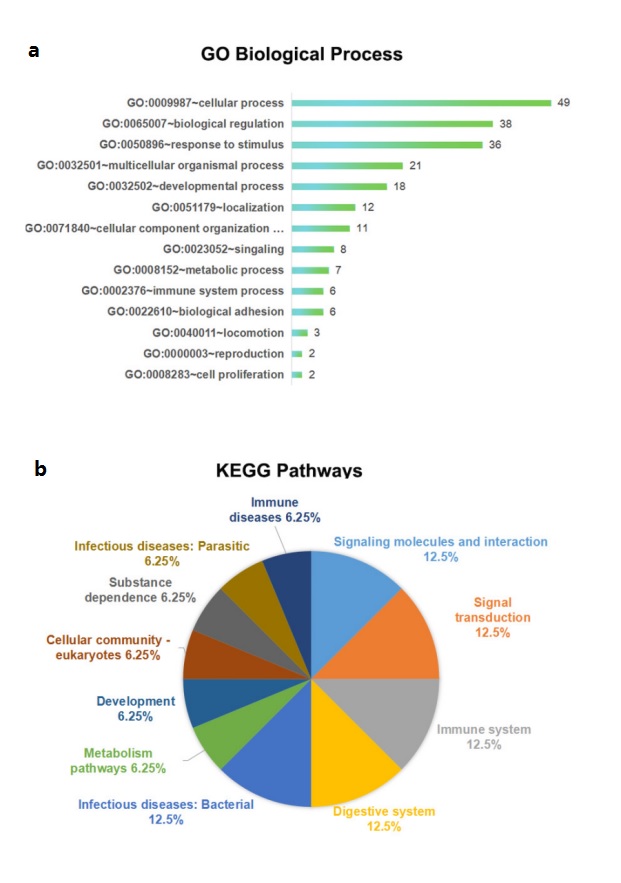


**Figure. S5. The distributions on the categories of the enriched GO biological processes (a) and KEGG pathways (b) from the targeted modular network of DHI**

**Figure. S6.**


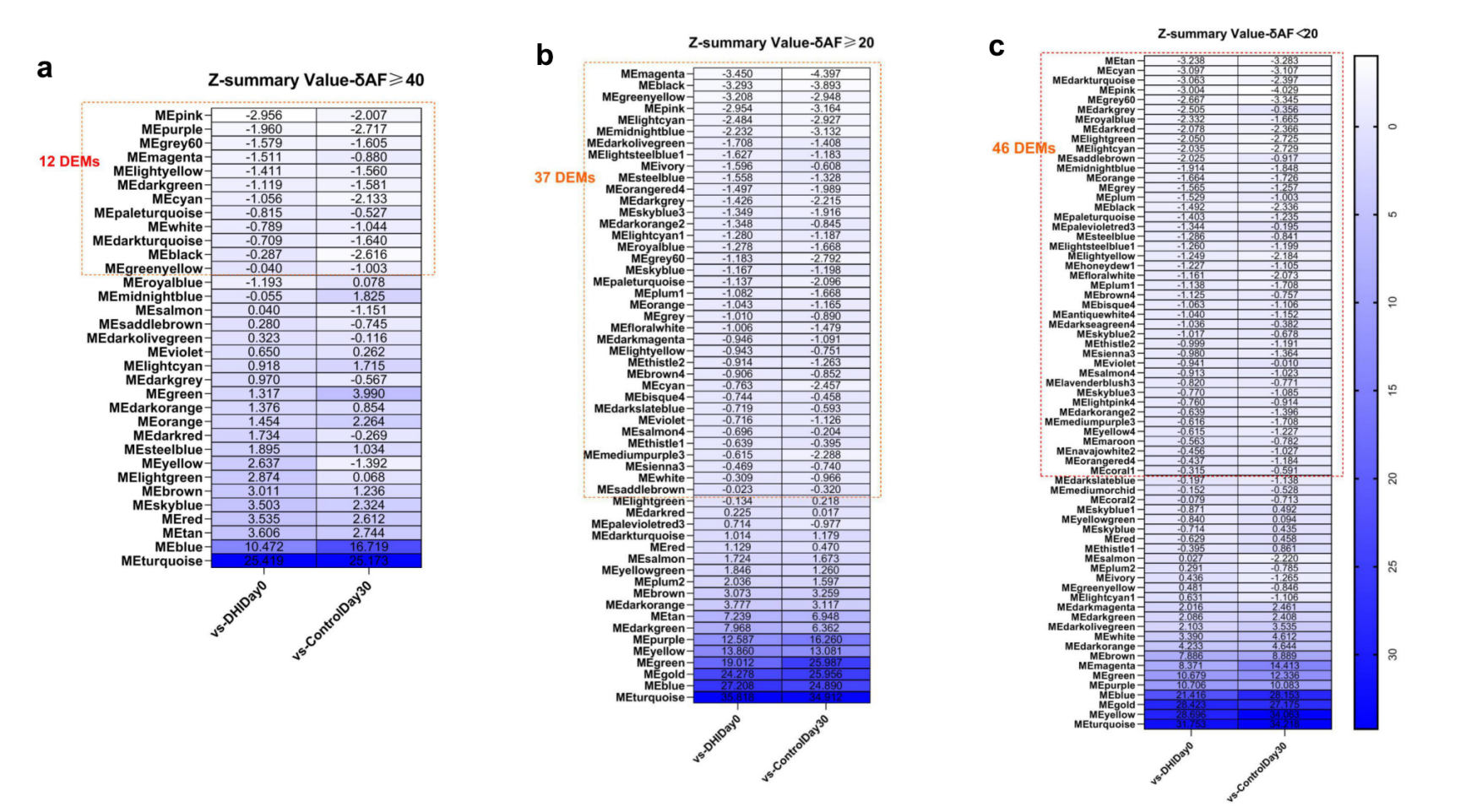


**Figure. S6. The differentially expressed modules (DEMs) at Day 30 detected in the populations with different effect according to Z_summary_ compared with the control group and theirselve’s Day 0**

**Figure. S7**

**
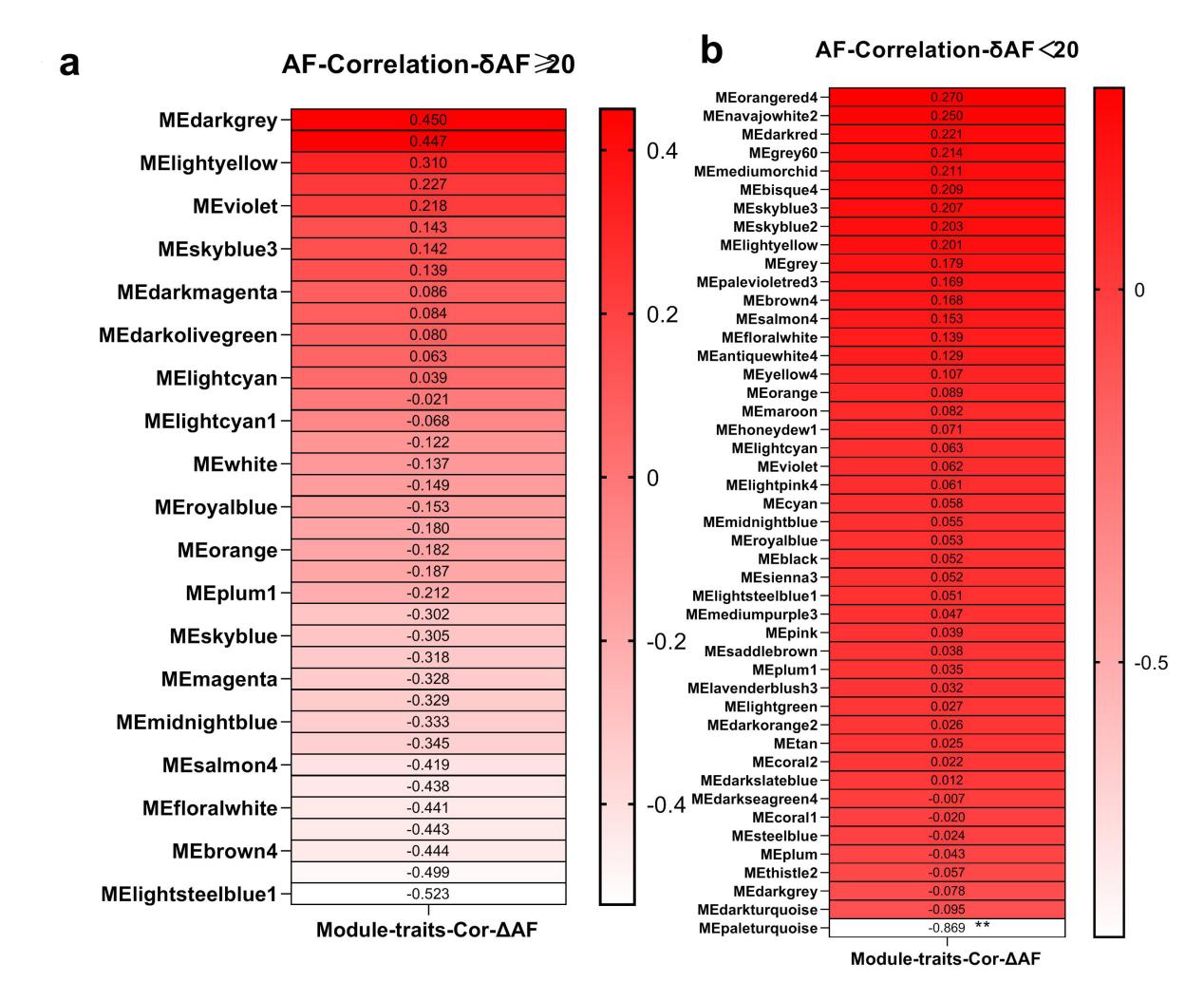
**

**Figure. S7 The corralation between theΔAF and the DEMs in the populations with a mild effect (40>ΔAF≥20) (a) and no effect (ΔAF<20) (b)**

**Table S1. Baseline characteristics of the study participants.**

| **Characteristics** | | **DHI group***  **(N=613)** | **Control group**  **(N= 305)** | | **P value †** |
| --- | --- | --- | --- | --- | --- |
|  |  |  |  |  |  |
| **Sex—No.(%)** | | | | | 0.1553 |
| **Male** | 407(66.39) | | 188(61.64) | |  |
| **Female** | 206(33.61) | | 117(38.36) | |  |
| **Age (yrs) (mean(SD))** | 59.50(6.93) | | 60.01(6.75) | | 0.2828 |
| **BMI(kg/m2) (mean(SD))** | 24.68(2.93) | | 24.64(2.77) | | 0.8318 |
| **Prior optimal medical therapy for angina—No.(%)** | | | | | 0.5520 |
| **Yes** | | 577(94.13) | 290(95.08) | |  |
| **No** | | 36(5.87) | 15(4.92) | |  |
| **Diabetes—No.(%)** | | 51(8.32) | 21(6.89) | | 0.4464 |
| **Hypertension—No.(%)** | | 304(49.59) | 154(50.49) | | 0.7974 |
| **Hyperlipidemia—No.(%)** | | 46(7.50) | 20(6.56) | | 0.6010 |
| **History of myocardial infarction—No.(%)** | | 115(18.76) | 43(14.10) | | 0.0780 |
| **SAQ§ (mean(SD))** | | | | | |
| **Angina frequency** | | 56.44(22.34) | 58.00(20.95) | | 0.3106 |
| **Physical limitation** | | 67.18(12.45) | 66.96(12.32) | | 0.7941 |
| **Angina stability** | | 43.03(21.28) | 42.30(20.13) | | 0.6178 |
| **Treatment satisfaction** | | 52.40(18.97) | 54.29(18.60) | | 0.1534 |
| **Quality of life** | | 38.42(18.64) | 38.82(18.72) | | 0.7555 |
| **TCM syndrome score§ (mean(SD))** | | 21.43(7.95) | 20.96(7.15) | | 0.3853 |
| **CCS angina class****—No.(%)§** | | | | | 0.9715 |
| **II** | | 487(79.45) | | 242(79.34) |  |
| **III** | | 126(20.55) | | 63(20.66) |  |
| **ECG—No.(%)¶** | | | | | 0.4517 |
| **Normal** | | 249(40.95) | | 132(43.56) |  |
| **Abnormal** | | 359(59.05) | | 171(56.44) |  |
| **Pulse rate (bpm) (mean(SD))** | | 68.90(8.96) | | 68.72(8.29) | 0.7700 |
| **Blood pressure (mmHg) (mean(SD))** | | | | | |
| **Systolic** | | 127.66(12.63) | | 127.03(10.98) | 0.4581 |
| **Diastolic** | | 77.43(9.71) | | 76.44(8.01) | 0.1244 |
| **Plasma lipid level (mmol/L) (mean(SD))** | | | | |  |
| **Total cholesterol** | | 4.40(1.35) | | 4.44(1.14) | 0.5934 |
| **LDL cholesterol** | | 2.44(0.90) | | 2.48(0.95) | 0.5775 |
| **HDL cholesterol** | | 1.21(0.30) | | 1.22(0.34) | 0.9310 |
| **Triglyceride** | | 1.78(1.44) | | 1.84(1.20) | 0.5399 |
| **CRP (mg/L)& (median(P25-P75))** | | 1.87(0.50-3.54) | | 1.43(0.50-3.18) | 0.2009 |
| **hs-CRP (mg/L)& (median(P25-P75))** | | 1.28(0.64-2.76) | | 1.51(0.69-3.89) | 0.1409 |
| **PAR# (mean(SD))** | | 56.44(20.80) | | 53.08(21.37) | 0.2254 |

* DHI denotes Danhong injection.

**†** P values were not adjusted for multiple comparisons.

§“SAQ” refers to the Seattle Angina Questionnaire; “TCM” refers to traditional Chinese medicine; “CCS” refers to the Canadian Cardiovascular Society.

¶ “ECG” refers to an electrocardiogram.

& The levels of CRP were measured in 256 patients (176 in the DHI group vs. 80 in the control group), and those of hs-CRP was measured in 560 patients (372 in the DHI group vs. 188 in the control group). The median(P25-P75) was used to describe the central tendency, and the differences between groups were tested for statistical significance using the Wilcoxon rank-sum test.

# “PAR” refers to the platelet aggregation rate. PAR values were determined in only 243 patients (163 in the DHI group vs. 80 in the control group) due to the unavailability of the test at some study sites.

**Table S2 The concomitant medications between two groups***

| **Concomitant drugs** | **DHI^#^**  **(n=604)** | **Control**  **(n=302)** | **P value** |
| --- | --- | --- | --- |
| **Clopidogrel** |  |  |  |
| No n(%) | 443(73.34) | 215(71.19) | 0.4934 |
| Yes n(%) | 161(26.66) | 87(28.81) |  |
| **Aspirin** |  |  |  |
| No n(%) | 58(9.60) | 28(9.27) | 0.8726 |
| Yes n(%) | 546(90.40) | 274(90.73) |  |
| **Stains** |  |  |  |
| No n(%) | 53(8.77) | 31(10.26) | 0.4660 |
| Yes n(%) | 551(91.23) | 271(89.74) |  |
| **Calcium channel blockers** |  |  |  |
| No n(%) | 438(72.52) | 222(73.51) | 0.7513 |
| Yes n(%) | 166(27.48) | 80(26.49) |  |
| **Angiotensin II-receptor blockers** |  |  |  |
| No n(%) | 459(75.99) | 227(75.17) | 0.7841 |
| Yes n(%) | 145(24.01) | 75(24.83) |  |
| **Angiotensin converting enzyme inhibitors** | | | |
| No n(%) | 509(84.27) | 258(85.43) | 0.6482 |
| Yes n(%) | 95(15.73) | 44(14.57) |  |

***** The concomitant medications were analysis according to the Safety Analyses. P values were not adjusted for multiple testing.

# DHI denotes Donhong injection.

**Table S3 Patients with clinically significant improvement from baseline in scores on the Seattle Angina Questionnaire**

| **Domain** | **DHI group***  **(N=613)** | **Control group**  **(N=305)** | **Difference**  **(95%CI)** | **P Value ^†^** |
| --- | --- | --- | --- | --- |
|  | **No. of patients with improvement(%)** | |  |  |
| **Primary Outcome^#^** | | | | |
| **Angina frequency**  Day 30 | | | | |
| **not imputed** | 308/585(52.65) | 115/289(39.79) | / | 0.0003 |
| **imputed** | 52.41(48.37,56.44) | 39.62(34.00,45.24) | 12.78(5.86, 19.71) | 0.0003 |
| **Secondary outcome^$^** | |  |  |  |
| **Angina frequency** | | |  |  |
| Day 60 | 356/585(60.85) | 135/287(47.04) | 13.82(6.82,20.82) | 0.0001 |
| Day 90 | 386/584(66.10) | 164/287(57.14) | 8.95(2.06,15.85) | 0.0100 |
| **Physical limitation** | | | | |
| Day 30 | 226/585(38.63) | 79/289(27.34) | 11.30(4.82,17.78) | 0.0010 |
| Day 60 | 271/585(46.32) | 94/287(32.75) | 13.57(6.80,20.34) | 0.0001 |
| Day 90 | 291/584(49.83) | 109/287(37.98) | 11.85(4.92,18.78) | 0.0010 |
| **Angina stability** | | | | |
| Day 30 | 466/585(79.66) | 191/289(66.09) | 13.57(7.21,19.93) | <0.0001 |
| Day 60 | 390/585(66.67) | 165/287(57.49) | 9.18(2.30,16.05) | 0.0081 |
| Day 90 | 384/584(65.75) | 172/287(59.93) | 5.82(-1.03,12.68) | 0.0927 |
| **Treatment satisfaction** | | | | |
| Day 30 | 287/585(49.06) | 96/289(33.22) | 15.84(9.07,22.62) | <0.0001 |
| Day 60 | 322/585(55.04) | 109/287(37.98) | 17.06(10.15,23.98) | <0.0001 |
| Day 90 | 334/584(57.19) | 122/287(42.51) | 14.68(7.70,21.67) | <0.0001 |
| **Quality of life** | | | | |
| Day 30 | 271/585 (46.32) | 109/289 (37.72) | 8.61(1.71,15.50) | 0.0157 |
| Day 60 | 313/585 (53.50) | 122/287 (42.51) | 11.00(3.99,18.00) | 0.0023 |
| Day 90 | 340/584 (58.22) | 132/287 (45.99) | 12.23(5.21,19.24) | 0.0007 |

* DHI denotes Donhong injection.

^†^ P values were not adjusted for multiple testing.

#Missing data multiple imputed for the primary outcome analysis; The number of participants with imputed data: 28(4.57%) for DHI group, 16(5.25%) for Control group.

$ Missing data not imputed for secondary outcome analysis.

**Table S4. Three sensitivity analysis for the changes from baseline in SAQ-AF scale***

| **Visit** | **Method** | **DHI（n=613）** | **Control（n=305）** | **Difference(95%CI)** | **P** |
| --- | --- | --- | --- | --- | --- |
| **Sensitivity analysis for center effects, LS mean (95% CI)**** | | | | | |
| Day 30 | ttest | 20.09(18.28, 21.91) | 13.78(11.79, 15.76) | 6.32(3.37, 9.26) | <.001 |
|  | ANCOVA | 19.14(17.78, 20.50) | 13.86(12.01, 15.72) | 5.28(3.08, 7.47) | <.001 |
|  | mixed | 19.21(16.25, 22.16) | 13.91(10.69, 17.12) | 5.30(3.11, 7.50) | <.001 |
| Day 60 | ttest | 24.25(22.39, 26.11) | 16.62(14.46, 18.79) | 7.63(4.57, 10.69) | <.001 |
|  | ANCOVA | 23.37(22.02, 24.72) | 16.87(15.03, 18.72) | 6.50(4.33, 8.67) | <.001 |
|  | mixed | 23.42(20.08, 26.76) | 16.90(13.33, 20.47) | 6.53(4.30, 8.75) | <.001 |
| Day 90 | ttest | 26.53(24.64, 28.42) | 20.33(18.04, 22.62) | 6.52(4.35, 8.69) | <.001 |
|  | ANCOVA | 26.14(24.77, 27.51) | 21.09(19.22, 22.95) | 5.05(2.86, 7.25) | <.001 |
|  | mixed | 26.13(22.76, 29.51) | 21.06(17.45, 24.67) | 5.07(2.88, 7.27) | <.001 |
| **Sensitivity analysis for departures from the missing at random assumption, LS mean (95% CI)^#^** | | | | | |
| Day 30 | mixed | 18.95(16.02, 21.88) | 13.86(10.68, 17.05) | 5.08(2.92, 7.25) | <.001 |
| Day 60 | mixed | 23.10(19.81, 26.40) | 16.88(13.36, 20.40) | 6.22(4.08, 8.37) | <.001 |
| Day 90 | mixed | 25.90(22.55, 29.24) | 21.06(17.49, 24.64) | 4.83(2.63, 7.03) | <.001 |
| **Sensitivity analysis using mixed-effect model with repeated measures method, LS mean (95% CI)^&^** | | | | | |
| Day 30 | mixed | 19.49(16.33, 22.65) | 14.21(10.81, 17.61) | 5.27(3.11, 7.43) | <.001 |
| Day 60 | mixed | 23.64(20.49, 26.79) | 17.06(13.69, 20.44) | 6.58(4.47, 8.69) | <.001 |
| Day 90 | mixed | 25.93(22.77, 29.09) | 20.77(17.36, 24.17) | 5.16(2.99, 7.34) | <.001 |

* In ANCOVA and mixed model, the differences is evaluated with least square(LS)mean. DHI, Danghong injection; CI, confidence interval; ANCOVA, analysis of covariance; LS, least squares; SAQ-AF:Seattle Angina Questionnaire angina-frequency scale.

** In our multicenter trials, randomization is carried out using permuted blocks stratified by center. To account for center effects, 3 three methods were used to adjust for center effects in the analysis. 1) without center effects: t-test with change of SAQ-AF score at Day 30/60/90 from baseline; 2) fixed center effects: ANCOVA with change from baseline as response variable, SAQ-AF score at baseline as a covariate, treatment and site as fixed effect; 3) random center effects: Mixed model with change from baseline as response variable, SAQ-AF score at baseline as a covariate, treatment as a fixed effect, site as random effects. The results of unadjusted and adjusted for center effects showed center factor did not affect the treatment effects.

^#^ The sensitivity analysis with a control-based pattern imputation model, assessed whether the change from baseline about AF scores at Day 30, Day 60 and Day 90 was robust to departure from missing at random missing at random. An imputation model for the missing observations in the control group was constructed from the observed data in the control group rather than the DHI.

^&^A sensitivity analysis also was performed using a mixed-effect model with repeated measures approach. The model included change from baseline to Day 30/60/90 as response variables, fixed-effects factors for treatment, visit, treatment × visit interaction, and SAQ-AF score at baseline as well as site and individual as the random effects. The model did not impute missing data points. An unstructured correlation matrix was used to model the within participant errors. Parameters were estimated using the maximum likelihood.

**Table S5 Freedom from angina over time as assessed with the** **angina-frequency scale of Seattle Angina Questionnaire (angina-frequency score of 100 on the Seattle Angina Questionnaire) according to treatment group.**

| Domain | DHI group^*^ | Control group | P Value^#^ |
| --- | --- | --- | --- |
|  | *No. of patients with angina-free(%)* | |  |
| Baseline | 9/613(1.47) | 4/305(1.31) | 1.0000 |
| Day 30 | 106/585(18.12) | 35/289(12.07) | 0.0219 |
| Day 60 | 156/585(26.67) | 48/287(16.67) | 0.0010 |
| Day 90 | 202/584(34.59) | 73/287(25.35) | 0.0057 |

* DHI denotes Donhong injection.

# The chi-square test was used to compare the proportions of patients in each treatment group who were angina-free as defined by the angina frequency score on the SAQ (with a score of 100 indicating that the patient was angina-free)

**Table. S6. Scores on the Seattle Angina Questionnaire from baseline to 90 days***

| **Domain** | **DHI**  **(N=613)** | **Control**  **(N=305)** | **P Value****†** |
| --- | --- | --- | --- |
| **Angina frequency** | | | |
| Baseline | 56.44±22.34 | 58.00±20.95 | 0.3106 |
| Day 30^a^ | 76.48±19.31 | 71.80±20.37 | 0.0010 |
| Day 60^b^ | 80.74±18.71 | 74.60±20.60 | <0.0001 |
| Day 90^c^ | 83.05±18.79 | 78.33±20.07 | 0.0007 |
| **Physical limitation** | | | |
| Baseline | 67.18±12.45 | 66.96±12.32 | 0.7941 |
| Day 30^a^ | 74.72±13.05 | 72.24±13.10 | 0.0084 |
| Day 60^b^ | 76.48±13.02 | 73.27±13.18 | 0.0007 |
| Day 90^c^ | 77.37±13.16 | 74.51±13.72 | 0.0031 |
| **Angina stability** | | | |
| Baseline | 43.03±21.28 | 42.30±20.13 | 0.6178 |
| Day 30^a^ | 80.90±19.61 | 71.19±22.31 | <0.0001 |
| Day 60^b^ | 72.39±22.68 | 66.46±22.74 | 0.0003 |
| Day 90^c^ | 71.49±23.49 | 67.51±23.01 | 0.0181 |
| **Treatment satisfaction** | | | |
| Baseline | 52.40±18.97 | 54.29±18.60 | 0.1534 |
| Day 30^a^ | 67.18±14.54 | 63.06±18.53 | 0.0003 |
| Day 60^b^ | 69.41±15.03 | 64.60±18.67 | <0.0001 |
| Day 90^c^ | 71.47±15.18 | 66.88±18.98 | 0.0001 |
| **Quality of life** | | | |
| Baseline | 38.42±18.64 | 38.82±18.72 | 0.7555 |
| Day 30^a^ | 51.61±16.71 | 48.62±19.74 | 0.0193 |
| Day 60^b^ | 54.56±16.88 | 51.13±20.33 | 0.0087 |
| Day 90^c^ | 56.82±17.60 | 52.90±20.73 | 0.0037 |

* Plus–minus values are means ±SD. DHI denotes Danhong injection

†P values were not adjusted for multiple testing.

1. Numbers of participants of Day 30: 585, DHI group, 289 and Control group.

b. Numbers of participants of Day 60: 585, DHI group and 287, Control group.

c. Numbers of participants of Day90: 584, DHI group and 287, Control group.

**Table S7. Changes in Xueyu-Zheng score from baseline to 90 days***

| **Domain** | **DHI group** | **Control group** | **P Value** |
| --- | --- | --- | --- |
|  | **N=**613 | **N=**305 |  |
| **Mean Xueyu-Zheng score (SD)** | | | |
| Baseline | 21.43±7.95 | 20.96±7.15 | 0.3853 |
| Day 7^a^ | 16.54±8.15 | 17.93±8.17 | 0.0165 |
| Day 14^b^ | 12.31±7.98 | 15.44±8.86 | <0.0001 |
| Day 30^c^ | 11.15±8.09 | 14.12±8.79 | <0.0001 |
| Day 60^d^ | 10.13±7.83 | 13.52±9.09 | <0.0001 |
| Day 90^e^ | 9.15±7.86 | 12.59±9.32 | <0.0001 |
| **No. of the patients with** **significant syndrome improvements in Xueyu-Zheng score(%)** | | | |
| Day 7^a^ | 198/598(33.11) | 62/297(20.88) | 0.0001 |
| Day 14^b^ | 380/590(64.41) | 136/292(46.58) | <0.0001 |
| Day 30^c^ | 422/582(72.51) | 150/289(51.90) | <0.0001 |
| Day 60^d^ | 448/582(76.98) | 161/286(56.29) | <0.0001 |
| Day 90^e^ | 474/583(81.30) | 169/287(58.89) | <0.0001 |

* Plus–minus values are means ±SD. DHI denotes Danhong injection

1. Numbers of participants of Day 7: 598, DHI group, 297 and Control group.

b. Numbers of participants of Day 14: 590, DHI group, 292 and Control group.

c. Numbers of participants of Day 30: 582, DHI group, 289 and Control group.

d. Numbers of participants of Day 60: 582, DHI group and 286, Control group.

e. Numbers of participants of Day90: 583, DHI group and 287, Control group.

**Table S8. Changes in angina frequency and the consumption of the nitroglycerin according to patients’ dairy**

| **Domain** | **DHI group** | | | **Control group** | | **P-value*** |
| --- | --- | --- | --- | --- | --- | --- |
|  | **No.** | **Value** | | **No.** | **Value** |  |
| **Incidence density for the angina frequency (attacks/person.day)** | | | | | | |
| Day 1-Day 7 | 599 | | 0.26 | 298 | 0.28 | 0.2504 |
| Day 8-Day 14 | 592 | | 0.18 | 291 | 0.20 | 0.0272 |
| Day 15- Day 30 | 587 | | 0.13 | 289 | 0.17 | <0.0001 |
| Day 31- Day 60 | 585 | | 0.11 | 287 | 0.16 | <0.0001 |
| Day 61- Day 90 | 583 | | 0.09 | 287 | 0.11 | <0.0001 |
| **Incidence density for the consumption of** **the nitroglycerin (pill/person.day)** | | | | | | |
| Day 1-Day 7 | 599 | | 0.22 | 298 | 0.22 | 0.6624 |
| Day 8-Day 14 | 592 | | 0.15 | 291 | 0.17 | 0.0984 |
| Day 15- Day 30 | 587 | | 0.11 | 289 | 0.15 | <0.0001 |
| Day 31- Day 60 | 585 | | 0.09 | 287 | 0.14 | <0.0001 |
| Day 61- Day 90 | 583 | | 0.08 | 287 | 0.10 | <0.0001 |

*****Poisson regression were used to compare difference of the incidence density between two groups.**Table S9. Changes in CCS grade and ECG recordings**

| **Domain** | **DHI group** | **Control group** | **P Value** |
| --- | --- | --- | --- |
| **Patients with improvement in CCS grade (NO./Total(%))** | | | |
| Day 7 | 73/600(12.17) | 25/299(8.36) | 0.0845 |
| Day 14 | 108/594(18.18) | 41/295(13.99) | 0.1165 |
| Day 30 | 151/588(25.68) | 58/289(20.07) | 0.0668 |
| Day 60 | 189/585(32.31) | 68/287(23.69) | 0.0087 |
| Day 90 | 212/584(36.30) | 82/288(28.47) | 0.0214 |
| **Patients with** **normal ECG recordings(NO./Total(%))** | | | |
| Baseline | 249/608(40.95) | 132/303(43.56) | 0.4517 |
| Day 7 | 250/588(42.52) | 122/290(42.07) | 0.8994 |
| Day 14 | 250/585(42.74) | 125/289(43.25) | 0.8844 |
| Day 30 | 252/578(43.60) | 130/286(45.45) | 0.6052 |
| Day 60 | 267/578(46.19) | 128/284(45.07) | 0.7557 |
| Day 90 | 278/577(48.18) | 137/283(48.41) | 0.9495 |

**Table S10. Changes in plasma lipid level, C-reactive protein and platelet aggregation rate**

| **Domain** | **DHI group** | | **Control group** | | **P-value** |
| --- | --- | --- | --- | --- | --- |
|  | **Mean(SD)** | **No(miss)** | **Mean(SD)** | **No(miss)** |  |
| **Plasma lipid level (mmol/L) at Day 14** | | | | | |
| Total cholesterol | 4.25(0.97) | 578(35) | 4.29(1.01) | 288(17) | 0.6318 |
| LDL-cholesterol | 2.36(0.81) | 581(32) | 2.40(0.83) | 286(19) | 0.5312 |
| HDL-cholesterol | 1.22(0.30) | 581(32) | 1.22(0.30) | 288(17) | 0.9211 |
| Triglyceride | 1.61(0.92) | 582(31) | 1.72(1.10) | 287(18) | 0.1222 |
| **High-sensitivity C-reactive protein (mg/L) at Day 14^#^** | 1.22(0.60-2.68) | 364(4) | 1.29(0.57-2.68) | 182(3) | 0.9310 |
| **C-reactive protein (mg/L) at Day 14^#^** | 1.12(0.45-2.90) | 161(13) | 1.00(0.40-2.72) | 80(7) | 0.7077 |
| **Platelet aggregation rate at Day 14** | 56.18(18.94) | 162(451) | 55.96(17.86) | 79(226) | 0.9285 |

# Median (P_25_-P_75_) was used to describe,Statistical differences between groups were analyzed using Wilcoxon rank-sum test

**Table S11. Adverse Events during the Study**

| **Events** | **DHI group**  **(N=604)** | **Control group**  **(N=302)** | **P-value** | **DHI group**  **(N=604)** | **Control group**  **(N=302)** | **P-value** |
| --- | --- | --- | --- | --- | --- | --- |
|  | ***No. of events*** | |  | ***No. of patients with event (%)*** | |  |
| **Any adverse event** | 93 | 58 | 0.1471 | 60(9.9) | 36(11.9) | 0.3597 |
| **Adverse events (except SAEs)** | 80 | 49 | 0.2262 | 57(9.4) | 34(11.3) | 0.3900 |
| **Cardiac disorders** | 3 | 2 |  | 2 | 1 |  |
| Chest discomfort | 1 | 1 |  | 1 | 1 |  |
| Palpitation | 2 | 0 |  | 2 | 0 |  |
| Dyspnoea | 0 | 1 |  | 0 | 1 |  |
| **Eye disorders** | 3 | 0 |  | 2 | 0 |  |
| Conjunctival disorder | 1 | 0 |  | 1 | 0 |  |
| Conjunctival haemorrhage | 1 | 0 |  | 1 | 0 |  |
| Retinal haemorrhage | 1 | 0 |  | 1 | 0 |  |
| **Gastrointestinal disorders** | 3 | 2 |  | 3 | 2 |  |
| Abdominal distension | 1 | 0 |  | 1 | 0 |  |
| Upper abdominal pain | 0 | 1 |  | 0 | 1 |  |
| Gastrointestinal haemorrhage | 1 | 0 |  | 1 | 0 |  |
| Chronic gastritis | 0 | 1 |  | 0 | 1 |  |
| Haemorrhoidal haemorrhage | 1 | 0 |  | 1 | 0 |  |
| **General disorders and administration site condition** | 3 | 2 |  | 3 | 1 |  |
| Asthenia | 1 | 0 |  | 1 | 0 |  |
| Chills | 0 | 1 |  | 0 | 1 |  |
| Inflammation | 1 | 0 |  | 1 | 0 |  |
| Pyrexia | 0 | 1 |  | 0 | 1 |  |
| Vessel puncture site inflammation | 1 | 0 |  | 1 | 0 |  |
| **Hepatobiliary disorders** | 1 | 6 |  | 1 | 6 |  |
| Abnormal hepatic function | 1 | 4 |  | 1 | 4 |  |
| Acute hepatitis | 0 | 1 |  | 0 | 1 |  |
| Liver injury | 0 | 1 |  | 0 | 1 |  |
| **Infections and infestations** | 19 | 10 |  | 18 | 9 |  |
| Conjunctivitis | 0 | 1 |  | 0 | 1 |  |
| Upper respiratory tract infection | 5 | 5 |  | 5 | 5 |  |
| Urinary tract infection | 14 | 4 |  | 14 | 4 |  |
| **Investigations** | 31 | 22 |  | 18 | 16 |  |
| Coagulation and bleeding analyses abnormal^$^ | 2 | 1 |  | 2 | 1 |  |
| Transaminases increased^#^ | 12 | 5 |  | 12 | 5 |  |
| Blood cholesterol increased | 1 | 0 |  | 1 | 0 |  |
| Blood triglycerides increased | 2 | 1 |  | 2 | 1 |  |
| Low density lipoprotein increased | 1 | 0 |  | 1 | 0 |  |
| Blood creatinine increased | 0 | 1 |  | 0 | 1 |  |
| Blood urea abnormal | 2 | 0 |  | 2 | 0 |  |
| White blood cell count decreased | 0 | 1 |  | 0 | 1 |  |
| Protein urine present | 2 | 2 |  | 2 | 2 |  |
| Blood urine present | 0 | 1 |  | 0 | 1 |  |
| Red blood cells urine positive | 2 | 2 |  | 2 | 2 |  |
| White blood cells urine positive | 3 | 2 |  | 3 | 2 |  |
| Occult blood positive | 0 | 2 |  | 0 | 2 |  |
| C-reactive protein increased | 4 | 4 |  | 4 | 4 |  |
| **Nervous system disorders** | 6 | 0 |  | 4 | 0 |  |
| Dizziness | 2 | 0 |  | 2 | 0 |  |
| Headache | 2 | 0 |  | 2 | 0 |  |
| Hypoaesthesia | 2 | 0 |  | 2 | 0 |  |
| **Metabolism and nutrition disorders** | 6 | 4 |  | 6 | 4 |  |
| Diabetes mellitus | 0 | 1 |  | 0 | 1 |  |
| Hyperlipidaemia | 6 | 3 |  | 6 | 3 |  |
| **Musculoskeletal and connective tissue disorders** | 1 | 0 |  | 1 | 0 |  |
| Arthralgia | 1 | 0 |  | 1 | 0 |  |
| **Psychiatric disorders** | 2 | 0 |  | 2 | 0 |  |
| Insomnia | 1 | 0 |  | 1 | 0 |  |
| Tension | 1 | 0 |  | 1 | 0 |  |
| **Renal and urinary disorders** | 0 | 2 |  | 0 | 2 |  |
| Microalbuminuria | 0 | 1 |  | 0 | 1 |  |
| Haematuria | 0 | 1 |  | 0 | 1 |  |
| **Reproductive system and breast disorders** | 1 | 0 |  | 1 | 0 |  |
| Spontaneous penile erection | 1 | 0 |  | 1 | 0 |  |
| **Skin and subcutaneous tissue disorders** | 2 | 0 |  | 2 | 0 |  |
| Eczema | 1 | 0 |  | 1 | 0 |  |
| Rash | 1 | 0 |  | 1 | 0 |  |
| **Severe adverse event** | 13 | 9 | 0.4454 | 6 (1.0) | 4(1.3) | 0.9105 |
| **Cardiac disorders** | 8 | 3 |  | 5 | 2 |  |
| Acute coronary syndrome | 1 | 0 |  | 1 | 0 |  |
| Chest pain | 1 | 0 |  | 1 | 0 |  |
| Chest discomfort | 2 | 1 |  | 2 | 1 |  |
| Dyspnoea | 1 | 1 |  | 1 | 1 |  |
| Palpitation | 1 | 0 |  | 1 | 0 |  |
| Unstable angina | 2 | 0 |  | 2 | 0 |  |
| Acute myocardial infarction | 0 | 1 |  | 0 | 1 |  |
| **Nervous system disorders** | 1 | 2 |  | 1 | 1 |  |
| Headache | 1 | 0 |  | 1 | 0 |  |
| Dizziness | 0 | 1 |  | 0 | 1 |  |
| Vertebrobasilar insufficiency | 0 | 1 |  | 0 | 1 |  |
| **General disorders and administration site condition** | 3 | 0 |  | 2 | 0 |  |
| Asthenia | 1 | 0 |  | 1 | 0 |  |
| Cold sweat | 1 | 0 |  | 1 | 0 |  |
| Chills | 1 | 0 |  | 1 | 0 |  |
| **Psychiatric disorders** | 1 | 1 |  | 1 | 1 |  |
| Anxiety | 1 | 1 |  | 1 | 1 |  |
| **Infections and infestations** | 0 | 1 |  | 0 | 1 |  |
| Viral hepatitis | 0 | 1 |  | 0 | 1 |  |
| **Hepatobiliary disorders** | 0 | 1 |  | 0 | 1 |  |
| Cholestasis | 0 | 1 |  | 0 | 1 |  |
| **Gastrointestinal disorders** | 0 | 1 |  | 0 | 1 |  |
| Nausea | 0 | 1 |  | 0 | 1 |  |
| **New-onset major vascular events within 90 days** | **3** | **1** | **0.8502** | **3(0.5)** | **1(0.3)** | **0.8502** |
| Unstable angina | 2 | 0 | / | 2 | 0 | / |
| Acute coronary syndrome | 1 | 0 | / | 1 | 0 | / |
| Acute myocardial infarction | 0 | 1 | / | 0 | 1 | / |

**# “**Transaminases increased” indicates that either alanine or aspartate aminotransferase or both of them increased.

$ “Coagulation and bleeding analyses abnormal” indicates that any index abnormal in coagulation and bleeding analyses, here including activated partial thromboplastin time, prothrombin time and activated coagulation time.

**Table S12. Clinical characteristics of patients who provided serum samples for RNA sequencing.**

| **Characteristics** | | **Total**  **(N=62)** | | | **DHI group**  **(N=41 )** | | **Control group**  **(N= 21)** | **P value ^†^** |
| --- | --- | --- | --- | --- | --- | --- | --- | --- |
| **Sex —No. (%)** | | | | | | | | 0.6150 |
| Male | | 41 | | | 28 | | 13 |  |
| Female | | 21 | | | 13 | | 8 |  |
| **Age (yr) (Mean(Std))** | | 58(6) | | | 58(6) | | 57(7) | 0.6779 |
| **BMI(kg/m^2^) (Mean(Std))** | | 26.05(2.71) | | | 25.86(2.59) | | 26.41(2.96) | 0.4525 |
| **Prior intensive combination medical therapy for angina—No. (%)** | | | | | | | | 0.1135 |
| Yes | | 46 | | | 33 | 13 | |  |
| No | | 16 | | | 8 | 8 | |  |
| **Diabetes—No. (%)** | | 24 | | | 16 | 8 | | 0.9433 |
| **Hypertension—No. (%)** | | 46 | | | 30 | 16 | | 0.7970 |
| **Hyperlipidemia—No. (%)** | | 26 | | | 18 | 8 | | 0.6610 |
| **SAQ domains (Mean(Std))** | | | | | | | | |
| Angina frequency | | 64(18) | | | 63(19) | 67(15) | | 0.3494 |
| Physical limitation | | 71(9) | | | 72(10) | 69(7) | | 0.3370 |
| Angina stability | | 42(22) | | | 41(24) | 43(18) | | 0.7391 |
| Treatment satisfaction | | 66(12) | | | 66(13) | 65(10) | | 0.7536 |
| Quality of life | | 37(17) | | | 36(17) | 37(19) | | 0.8458 |
| **TCM syndrome score (Mean(Std))** | | 16.77(4.52) | | | 17.20(4.42) | 15.95(4.70) | | 0.3092 |
| **CCS angina class—No. (%)^§^** | | | | | | | | 0.8667 |
| II | 54 | | 35 | | | 19 | |  |
| III | 8 | | 6 | | | 2 | |  |
| **Plasma lipid level (mmol/L)(Mean(Std))** | | | | | | | |  |
| Total cholesterol | 3.98(1.12) | | | 4.08(1.25) | | 3.78(0.80) | | 0.3299 |
| LDL-cholesterol | 2.41(0.90) | | | 2.50(0.95) | | 2.23(0.78) | | 0.2540 |
| HDL-cholesterol | 1.21(0.36) | | | 1.21(0.31) | | 1.20(0.45) | | 0.8704 |
| Triglyceride | 1.56(0.93) | | | 1.68(1.04) | | 1.33(0.62) | | 0.1568 |
| **hs-C-reactive protein (mg/L)** | 2.07(3.62) | | | 2.10(4.04) | | 2.00(2.69 ) | | 0.9150 |
| **Platelet aggregation rate^#^** | 38.43(28.94) | | | 41.44(28.41) | | 33.00(30.61) | | 0.4826 |

† P value was calculated by t-test.

§“-testrefers to Canadian Cardiovascular Society.

# The 34 patients from Xuanwu Hospital did not receive the test of platelet aggregation rate.

**Table S13. The differentially expressed genes (mRNA and mi-RNA) before and after treatment with Danhong injection compared with control group**

| **ID** | **gene_symbol** | **Time_Diff** | **FoldChange**  **(groupB/groupA)** | **log2FoldChange(groupB/groupA)** | **Up/Down-Regulation** | **P-value** |
| --- | --- | --- | --- | --- | --- | --- |
| 1 | hsa-miR-1246 | day0-VS-day30 | 2.36 | 1.2382 | Up | 0.0262 |
| 2 | hsa-miR-1273f | day0-VS-day14 | 0.46 | -1.1316 | Down | 0.0424 |
| 3 | hsa-miR-1273f | day0-VS-day30 | 0.26 | -1.9257 | Down | 0.0206 |
| 4 | hsa-miR-3136-3p | day0-VS-day14 | 0.41 | -1.2845 | Down | 0.0105 |
| 5 | hsa-miR-3136-3p | day0-VS-day30 | 0.36 | -1.4842 | Down | 0.0078 |
| 6 | hsa-miR-3166 | day0-VS-day14 | 2.49 | 1.3151 | Up | 0.0060 |
| 7 | hsa-miR-3180-5p | day0-VS-day30 | 2.96 | 1.5654 | Up | 0.0203 |
| 8 | hsa-miR-3200-5p | day0-VS-day30 | 2.14 | 1.0990 | Up | 0.0311 |
| 9 | hsa-miR-374c-5p | day0-VS-day30 | 0.47 | -1.0939 | Down | 0.0353 |
| 10 | hsa-miR-3941 | day0-VS-day30 | 2.91 | 1.5400 | Up | 0.0070 |
| 11 | hsa-miR-4430 | day0-VS-day14 | 1.66 | 0.7323 | Up | 0.0425 |
| 12 | hsa-miR-4461 | day0-VS-day30 | 0.46 | -1.1341 | Down | 0.0267 |
| 13 | hsa-miR-548ad-5p | day0-VS-day30 | 0.48 | -1.0703 | Down | 0.0380 |
| 14 | hsa-miR-548az-5p | day0-VS-day14 | 2.54 | 1.3473 | Up | 0.0147 |
| 15 | hsa-miR-6848-5p | day0-VS-day30 | 2.1 | 1.0738 | Up | 0.0328 |
| 16 | hsa-miR-6874-5p | day0-VS-day14 | 2.33 | 1.2184 | Up | 0.0372 |
| 17 | hsa-miR-7151-3p | day0-VS-day14 | 2.9 | 1.5346 | Up | 0.0428 |
| 18 | hsa-miR-7151-3p | day0-VS-day30 | 3.4 | 1.7663 | Up | 0.0428 |
| 19 | novel_mir_240 | day0-VS-day14 | 2.87 | 1.5225 | Up | 0.0174 |
| 20 | novel_mir_56 | day0-VS-day14 | 3.13 | 1.6474 | Up | 0.0353 |
| 21 | novel_mir_612 | day0-VS-day30 | 3.8 | 1.9249 | Up | 0.0340 |
| 22 | novel_mir_906 | day0-VS-day14 | 1.55 | 0.6342 | Up | 0.0476 |
| 23 | novel_mir_95 | day0-VS-day30 | 4.68 | 2.2268 | Up | 0.0292 |
| 24 | novel_mir_97 | day0-VS-day30 | 0.39 | -1.3619 | Down | 0.0379 |
| 25 | ACADL | day0-VS-day30 | 1.85 | 0.8886 | Up | 0.0132 |
| 26 | ACOXL | day0-VS-day30 | 1.88 | 0.9100 | Up | 0.0146 |
| 27 | ADAMTS14 | day0-VS-day30 | 0.64 | -0.6420 | Down | 0.0389 |
| 28 | ADAMTS7 | day0-VS-day30 | 2.69 | 1.4297 | Up | 0.0229 |
| 29 | ADH1A | day0-VS-day14 | 0.47 | -1.0776 | Down | 0.0281 |
| 30 | ADORA3 | day0-VS-day30 | 1.53 | 0.6176 | Up | 0.0303 |
| 31 | APLP1 | day0-VS-day30 | 2.1 | 1.0682 | Up | 0.0195 |
| 32 | AQP5 | day0-VS-day30 | 1.95 | 0.9647 | Up | 0.0242 |
| 33 | AQP7P1 | day0-VS-day14 | 2.19 | 1.1296 | Up | 0.0309 |
| 34 | ARHGEF15 | day0-VS-day30 | 2.3 | 1.2013 | Up | 0.0153 |
| 35 | ART1 | day0-VS-day14 | 1.89 | 0.9160 | Up | 0.0205 |
| 36 | BSND | day0-VS-day14 | 2.03 | 1.0186 | Up | 0.0214 |
| 37 | C12orf40 | day0-VS-day30 | 0.38 | -1.3778 | Down | 0.0078 |
| 38 | C19orf26 | day0-VS-day30 | 2.29 | 1.1968 | Up | 0.0100 |
| 39 | C1orf95 | day0-VS-day30 | 0.54 | -0.8792 | Down | 0.0485 |
| 40 | C6orf165 | day0-VS-day14 | 0.53 | -0.9179 | Down | 0.0121 |
| 41 | C6orf223 | day0-VS-day30 | 2.29 | 1.1967 | Up | 0.0129 |
| 42 | CCDC42 | day0-VS-day30 | 0.61 | -0.7187 | Down | 0.0301 |
| 43 | CCDC80 | day0-VS-day30 | 2.39 | 1.2591 | Up | 0.0077 |
| 44 | CCER2 | day0-VS-day30 | 0.64 | -0.6329 | Down | 0.0220 |
| 45 | CEACAM7 | day0-VS-day30 | 0.51 | -0.9688 | Down | 0.0350 |
| 46 | CT55 | day0-VS-day30 | 0.55 | -0.8566 | Down | 0.0327 |
| 47 | DIO2 | day0-VS-day30 | 2.1 | 1.0703 | Up | 0.0307 |
| 48 | DPY19L2P2 | day0-VS-day14 | 0.54 | -0.8995 | Down | 0.0238 |
| 49 | DPY19L2P2 | day0-VS-day30 | 0.56 | -0.8405 | Down | 0.0238 |
| 50 | DYRK3 | day0-VS-day14 | 1.6 | 0.6761 | Up | 0.0448 |
| 51 | EDA2R | day0-VS-day30 | 0.62 | -0.6844 | Down | 0.0216 |
| 52 | ERAS | day0-VS-day30 | 1.89 | 0.9150 | Up | 0.0315 |
| 53 | FAM221B | day0-VS-day14 | 0.57 | -0.8049 | Down | 0.0382 |
| 54 | GCOM1 | day0-VS-day30 | 1.58 | 0.6581 | Up | 0.0422 |
| 55 | GDF6 | day0-VS-day30 | 0.51 | -0.9627 | Down | 0.0499 |
| 56 | GIPC2 | day0-VS-day30 | 0.49 | -1.0330 | Down | 0.0264 |
| 57 | GLUD1P7 | day0-VS-day14 | 0.41 | -1.2760 | Down | 0.0485 |
| 58 | GPRC5A | day0-VS-day30 | 2.45 | 1.2922 | Up | 0.0103 |
| 59 | HGC6.3 | day0-VS-day30 | 0.49 | -1.0428 | Down | 0.0387 |
| 60 | HIST1H1B | day0-VS-day30 | 2.74 | 1.4529 | Up | 0.0050 |
| 61 | HIST1H2BM | day0-VS-day30 | 1.79 | 0.8433 | Up | 0.0287 |
| 62 | IDH1-AS1 | day0-VS-day30 | 1.78 | 0.8351 | Up | 0.0455 |
| 63 | IL20RA | day0-VS-day14 | 0.58 | -0.7755 | Down | 0.0274 |
| 64 | IL20RA | day0-VS-day30 | 0.43 | -1.2054 | Down | 0.0115 |
| 65 | IL36A | day0-VS-day14 | 0.42 | -1.2683 | Down | 0.0031 |
| 66 | IL36A | day0-VS-day30 | 0.63 | -0.6686 | Down | 0.0273 |
| 67 | KCNC2 | day0-VS-day14 | 1.9 | 0.9279 | Up | 0.0497 |
| 68 | LINC00515 | day0-VS-day14 | 2.86 | 1.5155 | Up | 0.0013 |
| 69 | LINC00515 | day0-VS-day30 | 2.45 | 1.2931 | Up | 0.0153 |
| 70 | LOC100630918 | day0-VS-day14 | 0.66 | -0.5944 | Down | 0.0210 |
| 71 | LOC286190 | day0-VS-day30 | 0.65 | -0.6316 | Down | 0.0341 |
| 72 | LOC645752 | day0-VS-day14 | 2 | 1.0015 | Up | 0.0475 |
| 73 | LOC649330 | day0-VS-day30 | 1.59 | 0.6729 | Up | 0.0281 |
| 74 | LURAP1L | day0-VS-day30 | 0.44 | -1.1908 | Down | 0.0485 |
| 75 | MAFA | day0-VS-day14 | 0.43 | -1.2243 | Down | 0.0169 |
| 76 | MAG | day0-VS-day14 | 0.44 | -1.1908 | Down | 0.0060 |
| 77 | MAG | day0-VS-day30 | 0.57 | -0.8174 | Down | 0.0181 |
| 78 | MEGF11 | day0-VS-day14 | 1.67 | 0.7357 | Up | 0.0288 |
| 79 | MEGF11 | day0-VS-day30 | 1.66 | 0.7282 | Up | 0.0479 |
| 80 | MIR194-1 | day0-VS-day14 | 1.52 | 0.6051 | Up | 0.0413 |
| 81 | MIR320C1 | day0-VS-day14 | 0.65 | -0.6220 | Down | 0.0428 |
| 82 | MIR339 | day0-VS-day14 | 0.54 | -0.9017 | Down | 0.0348 |
| 83 | MIR4481 | day0-VS-day30 | 2.17 | 1.1166 | Up | 0.0100 |
| 84 | MIR4658 | day0-VS-day30 | 2.12 | 1.0841 | Up | 0.0269 |
| 85 | MIR4712 | day0-VS-day30 | 0.36 | -1.4885 | Down | 0.0308 |
| 86 | MIR5010 | day0-VS-day14 | 1.89 | 0.9158 | Up | 0.0401 |
| 87 | MIR5194 | day0-VS-day30 | 1.87 | 0.9050 | Up | 0.0212 |
| 88 | MIR630 | day0-VS-day14 | 2.5 | 1.3214 | Up | 0.0169 |
| 89 | MIR630 | day0-VS-day30 | 2.21 | 1.1408 | Up | 0.0464 |
| 90 | MRAP2 | day0-VS-day14 | 0.57 | -0.8049 | Down | 0.0331 |
| 91 | NKX2-1 | day0-VS-day30 | 1.99 | 0.9899 | Up | 0.0461 |
| 92 | NXF5 | day0-VS-day30 | 0.52 | -0.9298 | Down | 0.0410 |
| 93 | OPN1MW | day0-VS-day30 | 0.42 | -1.2388 | Down | 0.0159 |
| 94 | OR1J1 | day0-VS-day30 | 2.64 | 1.3997 | Up | 0.0045 |
| 95 | OR2C1 | day0-VS-day14 | 2.04 | 1.0252 | Up | 0.0157 |
| 96 | PCDH17 | day0-VS-day30 | 2.01 | 1.0052 | Up | 0.0437 |
| 97 | PDZRN3 | day0-VS-day30 | 1.88 | 0.9071 | Up | 0.0422 |
| 98 | PIP5K1P1 | day0-VS-day14 | 0.58 | -0.7838 | Down | 0.0225 |
| 99 | PKNOX2 | day0-VS-day14 | 0.46 | -1.1250 | Down | 0.0151 |
| 100 | PRSS1 | day0-VS-day30 | 0.57 | -0.8120 | Down | 0.0417 |
| 101 | PTTG2 | day0-VS-day30 | 2.12 | 1.0841 | Up | 0.0267 |
| 102 | RARRES1 | day0-VS-day14 | 2.36 | 1.2396 | Up | 0.0191 |
| 103 | RNU1-1 | day0-VS-day30 | 2.62 | 1.3909 | Up | 0.0406 |
| 104 | RNU1-2 | day0-VS-day30 | 2.62 | 1.3909 | Up | 0.0406 |
| 105 | RNU1-27P | day0-VS-day30 | 2.62 | 1.3909 | Up | 0.0406 |
| 106 | RNU1-28P | day0-VS-day30 | 2.62 | 1.3909 | Up | 0.0406 |
| 107 | RNU1-3 | day0-VS-day30 | 2.62 | 1.3909 | Up | 0.0406 |
| 108 | RNU1-4 | day0-VS-day30 | 2.62 | 1.3909 | Up | 0.0406 |
| 109 | RNU5D-1 | day0-VS-day30 | 2.55 | 1.3529 | Up | 0.0209 |
| 110 | RNVU1-18 | day0-VS-day30 | 2.62 | 1.3909 | Up | 0.0406 |
| 111 | RPL13AP20 | day0-VS-day30 | 2.4 | 1.2648 | Up | 0.0295 |
| 112 | RPPH1 | day0-VS-day30 | 2.18 | 1.1262 | Up | 0.0153 |
| 113 | RTN4RL2 | day0-VS-day30 | 1.61 | 0.6894 | Up | 0.0373 |
| 114 | SCARA3 | day0-VS-day30 | 1.81 | 0.8567 | Up | 0.0206 |
| 115 | SELV | day0-VS-day30 | 1.89 | 0.9165 | Up | 0.0247 |
| 116 | SERPINC1 | day0-VS-day14 | 1.72 | 0.7790 | Up | 0.0491 |
| 117 | SHC4 | day0-VS-day30 | 0.53 | -0.9258 | Down | 0.0437 |
| 118 | SLIT3 | day0-VS-day30 | 1.89 | 0.9196 | Up | 0.0231 |
| 119 | SNORA21 | day0-VS-day30 | 0.52 | -0.9467 | Down | 0.0290 |
| 120 | SNORA2A | day0-VS-day14 | 0.58 | -0.7768 | Down | 0.0436 |
| 121 | SNORA76A | day0-VS-day30 | 0.55 | -0.8621 | Down | 0.0499 |
| 122 | SNORD116-1 | day0-VS-day14 | 2.05 | 1.0343 | Up | 0.0056 |
| 123 | SNORD19B | day0-VS-day30 | 0.58 | -0.7738 | Down | 0.0282 |
| 124 | SNORD35A | day0-VS-day30 | 0.65 | -0.6288 | Down | 0.0498 |
| 125 | SNORD36C | day0-VS-day14 | 0.65 | -0.6301 | Down | 0.0221 |
| 126 | SNORD36C | day0-VS-day30 | 0.58 | -0.7783 | Down | 0.0057 |
| 127 | SP6 | day0-VS-day14 | 0.4 | -1.3236 | Down | 0.0052 |
| 128 | TBC1D29 | day0-VS-day30 | 2.04 | 1.0280 | Up | 0.0248 |
| 129 | TBC1D3B | day0-VS-day14 | 2.4 | 1.2659 | Up | 0.0346 |
| 130 | TCP10 | day0-VS-day30 | 0.49 | -1.0290 | Down | 0.0433 |
| 131 | TMC2 | day0-VS-day14 | 1.62 | 0.6953 | Up | 0.0452 |
| 132 | TMEM108 | day0-VS-day30 | 0.6 | -0.7410 | Down | 0.0477 |
| 133 | TMEM132B | day0-VS-day14 | 2.19 | 1.1314 | Up | 0.0226 |
| 134 | TOX3 | day0-VS-day30 | 1.79 | 0.8369 | Up | 0.0076 |
| 135 | TPTE | day0-VS-day30 | 0.47 | -1.0836 | Down | 0.0186 |
| 136 | ZNF541 | day0-VS-day30 | 0.64 | -0.6433 | Down | 0.0123 |

**Table S14. The Z-_summary_ value and the name of the 25 targeted pharmacological modules of Danhong injection versus placebo at Day 30**

| **Module color** | **Module size** | **Z-summary value** | **Module name** | **Degree in the targeted module network** | **Hub gene** | | **Most significant enriched GO-BP** | | **Most significant enriched KEGG pathway** | |
| --- | --- | --- | --- | --- | --- | --- | --- | --- | --- | --- |
|  |  |  |  |  | **name** | **degree** | **name** | **P value** | **name** | **P value** |
| Magenta | 140 | -4.4035 | Signal transduction-PI3K-Akt pathway | 24 | / | / | GO:0007165~signal transduction | 0.0305 | hsa04151:PI3K-Akt signaling pathway | 0.0028 |
| Tan | 70 | -3.4736 | Mesenchyme migration | 23 | / | / | GO:0090131~mesenchyme migration | 0.0116 | / | / |
| Cyan | 54 | -3.2231 | Detection of chemical stimulus involved in sensory perception | 23 | / | / | GO:0050907~detection of chemical stimulus involved in sensory perception | 0.0056 | / | / |
| Salmon | 57 | -3.1072 | Inflammatory response-Salmonella infection | 24 | / | / | GO:0006954~inflammatory response | 0.0001 | hsa05132:Salmonella infection | 2.3889E-5 |
| Darkgreen | 43 | -2.9934 | Fertilization-metabolic pathways | 22 | / | / | GO:0009566~fertilization | 0.0443 | hsa01100:Metabolic pathways | 0.03798 |
| Lightyellow | 45 | -2.9102 | G-protein coupled receptor signaling pathway-salivary secretion | 23 | / | / | GO:0007186~G-protein coupled receptor signaling pathway | 0.0111 | hsa04970:Salivary secretion | 0.03688 |
| Royalblue | 45 | -2.7266 | miR-1909-3p-PRR35 | 23 | miR-1909-3p、PRR35 | 44 | / | / | / | / |
| Lightcyan | 48 | -2.5957 | Regulation of G-protein coupled receptor protein signaling pathway | 22 | / | / | GO:0008277~regulation of G-protein coupled receptor protein signaling pathway | 0.0343 | / | / |
| Darkred | 43 | -2.5738 | miR-5586-5p-OR52N5 | 22 | miR-5586-5p、OR52N5 | 42 | / | / | / | / |
| White | 33 | -2.4898 | miR-206-OR2F1 | 22 | miR-206、OR2F1 | 32 | / | / | / | / |
| Darkorange | 36 | -2.4835 | Homophilic cell adhesion via plasma membrane adhesion molecules | 22 | / | / | GO:0007156~homophilic cell adhesion via plasma membrane adhesion molecules | 0.0055 | / | / |
| Pink | 148 | -2.4697 | Prepulse inhibition | 24 | / | / | GO:0060134~prepulse inhibition | 0.0268 | / | / |
| Darkturquoise | 41 | -2.4298 | miR-4709-5p- COL6A5 | 23 |  |  | / | / | / | / |
| Orange | 37 | -2.4188 | visual perception | 21 | / | / | GO:0007601~visual perception | 0.0213 | / | / |
| Lightgreen | 46 | -2.3658 | Osteoclast differentiation | 23 | / | / | / | / | hsa04380:Osteoclast differentiation | 0.0070 |
| Saddlebrown | 28 | -2.2169 | miR-4795-5p-LOC100506469 | 20 | miR-4795-5p、LOC100506469 | 27 | / | / | / | / |
| Darkolivegreen | 21 | -2.1020 | Antigen processing and presentation | 20 | / | / | GO:0019882~antigen processing and presentation | 0.0291 | / | / |
| Steelblue | 24 | -1.9231 | miR-4766-5p-ADCY10P1 | 23 | miR-4766-5p、ADCY10P1 | 23 | / | / | / | / |
| Paleturquoise | 22 | -1.6369 | Camera-type eye development | 21 | / | / | GO:0043010~camera-type eye development | 0.0189 | / | / |
| Grey60 | 48 | -1.3086 | Innate immune response | 24 | / | / | GO:0045087~innate immune response | 0.0061 | // | / |
| Violet | 21 | -1.2516 | Negative regulation of epithelial cell proliferation | 17 | / | / | GO:0050680~negative regulation of epithelial cell proliferation | 0.0457 | / | / |
| Sienna3 | 15 | -1.1164 | MIR568-SPEM1 | 13 | MIR568、SPEM1 | 14 | / | / | / | / |
| Yellowgreen | 14 | -0.9632 | miR-4670-3p-ZNF702P | 13 | miR-4670-3p、ZNF702P | 13 | / | / | / | / |
| Purple | 73 | -0.8462 | Regulation of signal transduction | 24 | / | / | GO:0009966~regulation of signal transduction | 0.0247 | / | / |
| Skyblue | 29 | -0.1165 | Response to calcium ion-Complement and coagulation cascades | 20 | / | / | GO:0051592~response to calcium ion | 0.0024 | hsa04610:Complement and coagulation cascades | 0.0061 |

**Table S15. The targeted modular map of 25 functional modules treated with DHI**

| **MODULE1** | **MODULE 2** | **No_Link** | **Connectivity Score** |
| --- | --- | --- | --- |
| Detection of chemical stimulus involved in sensory perception | Fertilization-metabolic pathways | 34 | 0.0146 |
| Detection of chemical stimulus involved in sensory perception | Antigen processing and presentation | 2 | 0.0018 |
| Detection of chemical stimulus involved in sensory perception | Homophilic cell adhesion via plasma membrane adhesion molecules | 38 | 0.0195 |
| Detection of chemical stimulus involved in sensory perception | miR-5586-5p-OR52N5 | 163 | 0.0702 |
| Detection of chemical stimulus involved in sensory perception | miR-4709-5p- COL6A5 | 6 | 0.0027 |
| Detection of chemical stimulus involved in sensory perception | Innate immune response | 136 | 0.0525 |
| Detection of chemical stimulus involved in sensory perception | Regulation of G-protein coupled receptor protein signaling pathway | 69 | 0.0266 |
| Detection of chemical stimulus involved in sensory perception | Osteoclast differentiation | 185 | 0.0745 |
| Detection of chemical stimulus involved in sensory perception | G-protein coupled receptor signaling pathway-salivary secretion | 65 | 0.0267 |
| Detection of chemical stimulus involved in sensory perception | Signal transduction-PI3K-Akt pathway | 302 | 0.0399 |
| Detection of chemical stimulus involved in sensory perception | visual perception | 24 | 0.0120 |
| Detection of chemical stimulus involved in sensory perception | Camera-type eye development | 44 | 0.0370 |
| Detection of chemical stimulus involved in sensory perception | Prepulse inhibition | 329 | 0.0412 |
| Detection of chemical stimulus involved in sensory perception | Regulation of signal transduction | 43 | 0.0109 |
| Detection of chemical stimulus involved in sensory perception | miR-1909-3p-PRR35 | 4 | 0.0016 |
| Detection of chemical stimulus involved in sensory perception | miR-4795-5p-LOC100506469 | 2 | 0.0013 |
| Detection of chemical stimulus involved in sensory perception | Inflammatory response-Salmonella infection | 118 | 0.0383 |
| Detection of chemical stimulus involved in sensory perception | Response to calcium ion-Complement and coagulation cascades | 85 | 0.0543 |
| Detection of chemical stimulus involved in sensory perception | miR-4766-5p-ADCY10P1 | 26 | 0.0201 |
| Detection of chemical stimulus involved in sensory perception | Mesenchyme migration | 567 | 0.1500 |
| Detection of chemical stimulus involved in sensory perception | Negative regulation of epithelial cell proliferation | 33 | 0.0291 |
| Detection of chemical stimulus involved in sensory perception | miR-206-OR2F1 | 22 | 0.0123 |
| Detection of chemical stimulus involved in sensory perception | miR-4670-3p-ZNF702P | 2 | 0.0026 |
| Fertilization-metabolic pathways | Antigen processing and presentation | 51 | 0.0565 |
| Fertilization-metabolic pathways | Homophilic cell adhesion via plasma membrane adhesion molecules | 24 | 0.0155 |
| Fertilization-metabolic pathways | miR-5586-5p-OR52N5 | 55 | 0.0297 |
| Fertilization-metabolic pathways | miR-4709-5p- COL6A5 | 67 | 0.0380 |
| Fertilization-metabolic pathways | Innate immune response | 33 | 0.0160 |
| Fertilization-metabolic pathways | Regulation of G-protein coupled receptor protein signaling pathway | 45 | 0.0218 |
| Fertilization-metabolic pathways | G-protein coupled receptor signaling pathway-salivary secretion | 41 | 0.0212 |
| Fertilization-metabolic pathways | Signal transduction-PI3K-Akt pathway | 243 | 0.0404 |
| Fertilization-metabolic pathways | visual perception | 88 | 0.0553 |
| Fertilization-metabolic pathways | Camera-type eye development | 21 | 0.0222 |
| Fertilization-metabolic pathways | Prepulse inhibition | 671 | 0.1054 |
| Fertilization-metabolic pathways | Regulation of signal transduction | 424 | 0.1351 |
| Fertilization-metabolic pathways | miR-1909-3p-PRR35 | 65 | 0.0336 |
| Fertilization-metabolic pathways | miR-4795-5p-LOC100506469 | 44 | 0.0365 |
| Fertilization-metabolic pathways | Inflammatory response-Salmonella infection | 43 | 0.0175 |
| Fertilization-metabolic pathways | Response to calcium ion-Complement and coagulation cascades | 1 | 0.0008 |
| Fertilization-metabolic pathways | miR-4766-5p-ADCY10P1 | 1 | 0.0010 |
| Fertilization-metabolic pathways | Mesenchyme migration | 131 | 0.0435 |
| Fertilization-metabolic pathways | Negative regulation of epithelial cell proliferation | 1 | 0.0011 |
| Fertilization-metabolic pathways | miR-206-OR2F1 | 43 | 0.0303 |
| Fertilization-metabolic pathways | miR-4670-3p-ZNF702P | 6 | 0.0100 |
| Antigen processing and presentation | Homophilic cell adhesion via plasma membrane adhesion molecules | 96 | 0.1270 |
| Antigen processing and presentation | miR-5586-5p-OR52N5 | 18 | 0.0199 |
| Antigen processing and presentation | miR-4709-5p- COL6A5 | 63 | 0.0732 |
| Antigen processing and presentation | Innate immune response | 57 | 0.0565 |
| Antigen processing and presentation | Regulation of G-protein coupled receptor protein signaling pathway | 10 | 0.0099 |
| Antigen processing and presentation | Osteoclast differentiation | 24 | 0.0248 |
| Antigen processing and presentation | G-protein coupled receptor signaling pathway-salivary secretion | 34 | 0.0360 |
| Antigen processing and presentation | Signal transduction-PI3K-Akt pathway | 35 | 0.0119 |
| Antigen processing and presentation | Camera-type eye development | 21 | 0.0455 |
| Antigen processing and presentation | Prepulse inhibition | 591 | 0.1902 |
| Antigen processing and presentation | Regulation of signal transduction | 193 | 0.1259 |
| Antigen processing and presentation | miR-1909-3p-PRR35 | 35 | 0.0370 |
| Antigen processing and presentation | miR-4795-5p-LOC100506469 | 2 | 0.0034 |
| Antigen processing and presentation | Inflammatory response-Salmonella infection | 2 | 0.0017 |
| Antigen processing and presentation | miR-4766-5p-ADCY10P1 | 16 | 0.0317 |
| Antigen processing and presentation | Mesenchyme migration | 2 | 0.0014 |
| Antigen processing and presentation | miR-206-OR2F1 | 49 | 0.0707 |
| Antigen processing and presentation | miR-4670-3p-ZNF702P | 28 | 0.0952 |
| Homophilic cell adhesion via plasma membrane adhesion molecules | miR-5586-5p-OR52N5 | 1 | 0.0006 |
| Homophilic cell adhesion via plasma membrane adhesion molecules | miR-4709-5p- COL6A5 | 40 | 0.0271 |
| Homophilic cell adhesion via plasma membrane adhesion molecules | Innate immune response | 108 | 0.0625 |
| Homophilic cell adhesion via plasma membrane adhesion molecules | Regulation of G-protein coupled receptor protein signaling pathway | 350 | 0.2025 |
| Homophilic cell adhesion via plasma membrane adhesion molecules | Osteoclast differentiation | 92 | 0.0556 |
| Homophilic cell adhesion via plasma membrane adhesion molecules | G-protein coupled receptor signaling pathway-salivary secretion | 67 | 0.0414 |
| Homophilic cell adhesion via plasma membrane adhesion molecules | Signal transduction-PI3K-Akt pathway | 162 | 0.0321 |
| Homophilic cell adhesion via plasma membrane adhesion molecules | visual perception | 58 | 0.0435 |
| Homophilic cell adhesion via plasma membrane adhesion molecules | Camera-type eye development | 1 | 0.0013 |
| Homophilic cell adhesion via plasma membrane adhesion molecules | Prepulse inhibition | 419 | 0.0786 |
| Homophilic cell adhesion via plasma membrane adhesion molecules | Regulation of signal transduction | 48 | 0.0183 |
| Homophilic cell adhesion via plasma membrane adhesion molecules | miR-1909-3p-PRR35 | 111 | 0.0685 |
| Homophilic cell adhesion via plasma membrane adhesion molecules | miR-4795-5p-LOC100506469 | 27 | 0.0268 |
| Homophilic cell adhesion via plasma membrane adhesion molecules | Inflammatory response-Salmonella infection | 109 | 0.0531 |
| Homophilic cell adhesion via plasma membrane adhesion molecules | Response to calcium ion-Complement and coagulation cascades | 51 | 0.0489 |
| Homophilic cell adhesion via plasma membrane adhesion molecules | miR-4766-5p-ADCY10P1 | 77 | 0.0891 |
| Homophilic cell adhesion via plasma membrane adhesion molecules | Mesenchyme migration | 188 | 0.0746 |
| Homophilic cell adhesion via plasma membrane adhesion molecules | miR-206-OR2F1 | 311 | 0.2618 |
| Homophilic cell adhesion via plasma membrane adhesion molecules | miR-4670-3p-ZNF702P | 19 | 0.0377 |
| miR-5586-5p-OR52N5 | miR-4709-5p- COL6A5 | 29 | 0.0164 |
| miR-5586-5p-OR52N5 | Innate immune response | 149 | 0.0722 |
| miR-5586-5p-OR52N5 | Regulation of G-protein coupled receptor protein signaling pathway | 16 | 0.0078 |
| miR-5586-5p-OR52N5 | Osteoclast differentiation | 62 | 0.0313 |
| miR-5586-5p-OR52N5 | G-protein coupled receptor signaling pathway-salivary secretion | 45 | 0.0233 |
| miR-5586-5p-OR52N5 | Signal transduction-PI3K-Akt pathway | 210 | 0.0349 |
| miR-5586-5p-OR52N5 | visual perception | 3 | 0.0019 |
| miR-5586-5p-OR52N5 | Camera-type eye development | 18 | 0.0190 |
| miR-5586-5p-OR52N5 | Prepulse inhibition | 169 | 0.0266 |
| miR-5586-5p-OR52N5 | Regulation of signal transduction | 128 | 0.0408 |
| miR-5586-5p-OR52N5 | miR-1909-3p-PRR35 | 46 | 0.0238 |
| miR-5586-5p-OR52N5 | miR-4795-5p-LOC100506469 | 28 | 0.0233 |
| miR-5586-5p-OR52N5 | Inflammatory response-Salmonella infection | 72 | 0.0294 |
| miR-5586-5p-OR52N5 | MIR568-SPEM1 | 41 | 0.0636 |
| miR-5586-5p-OR52N5 | Response to calcium ion-Complement and coagulation cascades | 25 | 0.0200 |
| miR-5586-5p-OR52N5 | miR-4766-5p-ADCY10P1 | 41 | 0.0397 |
| miR-5586-5p-OR52N5 | Mesenchyme migration | 28 | 0.0093 |
| miR-5586-5p-OR52N5 | miR-206-OR2F1 | 51 | 0.0359 |
| miR-4709-5p- COL6A5 | Innate immune response | 53 | 0.0269 |
| miR-4709-5p- COL6A5 | Regulation of G-protein coupled receptor protein signaling pathway | 121 | 0.0615 |
| miR-4709-5p- COL6A5 | Osteoclast differentiation | 128 | 0.0679 |
| miR-4709-5p- COL6A5 | G-protein coupled receptor signaling pathway-salivary secretion | 3 | 0.0016 |
| miR-4709-5p- COL6A5 | Signal transduction-PI3K-Akt pathway | 139 | 0.0242 |
| miR-4709-5p- COL6A5 | visual perception | 61 | 0.0402 |
| miR-4709-5p- COL6A5 | Camera-type eye development | 24 | 0.0266 |
| miR-4709-5p- COL6A5 | Prepulse inhibition | 385 | 0.0634 |
| miR-4709-5p- COL6A5 | Regulation of signal transduction | 140 | 0.0468 |
| miR-4709-5p- COL6A5 | miR-1909-3p-PRR35 | 25 | 0.0136 |
| miR-4709-5p- COL6A5 | Inflammatory response-Salmonella infection | 112 | 0.0479 |
| miR-4709-5p- COL6A5 | MIR568-SPEM1 | 15 | 0.0244 |
| miR-4709-5p- COL6A5 | Response to calcium ion-Complement and coagulation cascades | 18 | 0.0151 |
| miR-4709-5p- COL6A5 | miR-4766-5p-ADCY10P1 | 29 | 0.0295 |
| miR-4709-5p- COL6A5 | Mesenchyme migration | 28 | 0.0098 |
| miR-4709-5p- COL6A5 | Negative regulation of epithelial cell proliferation | 13 | 0.0151 |
| miR-4709-5p- COL6A5 | miR-206-OR2F1 | 179 | 0.1323 |
| miR-4709-5p- COL6A5 | miR-4670-3p-ZNF702P | 26 | 0.0453 |
| Innate immune response | Regulation of G-protein coupled receptor protein signaling pathway | 308 | 0.1337 |
| Innate immune response | Osteoclast differentiation | 114 | 0.0516 |
| Innate immune response | G-protein coupled receptor signaling pathway-salivary secretion | 48 | 0.0222 |
| Innate immune response | Signal transduction-PI3K-Akt pathway | 341 | 0.0507 |
| Innate immune response | visual perception | 49 | 0.0276 |
| Innate immune response | Camera-type eye development | 21 | 0.0199 |
| Innate immune response | Prepulse inhibition | 388 | 0.0546 |
| Innate immune response | Regulation of signal transduction | 66 | 0.0188 |
| Innate immune response | miR-1909-3p-PRR35 | 210 | 0.0972 |
| Innate immune response | miR-4795-5p-LOC100506469 | 405 | 0.3013 |
| Innate immune response | Inflammatory response-Salmonella infection | 230 | 0.0841 |
| Innate immune response | MIR568-SPEM1 | 16 | 0.0222 |
| Innate immune response | Response to calcium ion-Complement and coagulation cascades | 15 | 0.0108 |
| Innate immune response | miR-4766-5p-ADCY10P1 | 56 | 0.0486 |
| Innate immune response | Mesenchyme migration | 235 | 0.0699 |
| Innate immune response | Negative regulation of epithelial cell proliferation | 44 | 0.0437 |
| Innate immune response | miR-206-OR2F1 | 182 | 0.1149 |
| Innate immune response | miR-4670-3p-ZNF702P | 19 | 0.0283 |
| Regulation of G-protein coupled receptor protein signaling pathway | Osteoclast differentiation | 106 | 0.0480 |
| Regulation of G-protein coupled receptor protein signaling pathway | G-protein coupled receptor signaling pathway-salivary secretion | 116 | 0.0537 |
| Regulation of G-protein coupled receptor protein signaling pathway | Signal transduction-PI3K-Akt pathway | 208 | 0.0310 |
| Regulation of G-protein coupled receptor protein signaling pathway | visual perception | 42 | 0.0236 |
| Regulation of G-protein coupled receptor protein signaling pathway | Prepulse inhibition | 123 | 0.0173 |
| Regulation of G-protein coupled receptor protein signaling pathway | Regulation of signal transduction | 40 | 0.0114 |
| Regulation of G-protein coupled receptor protein signaling pathway | miR-1909-3p-PRR35 | 246 | 0.1139 |
| Regulation of G-protein coupled receptor protein signaling pathway | miR-4795-5p-LOC100506469 | 111 | 0.0826 |
| Regulation of G-protein coupled receptor protein signaling pathway | Inflammatory response-Salmonella infection | 106 | 0.0387 |
| Regulation of G-protein coupled receptor protein signaling pathway | MIR568-SPEM1 | 14 | 0.0194 |
| Regulation of G-protein coupled receptor protein signaling pathway | Response to calcium ion-Complement and coagulation cascades | 28 | 0.0201 |
| Regulation of G-protein coupled receptor protein signaling pathway | miR-4766-5p-ADCY10P1 | 60 | 0.0521 |
| Regulation of G-protein coupled receptor protein signaling pathway | Mesenchyme migration | 32 | 0.0095 |
| Regulation of G-protein coupled receptor protein signaling pathway | Negative regulation of epithelial cell proliferation | 13 | 0.0129 |
| Regulation of G-protein coupled receptor protein signaling pathway | miR-206-OR2F1 | 140 | 0.0884 |
| Osteoclast differentiation | G-protein coupled receptor signaling pathway-salivary secretion | 50 | 0.0242 |
| Osteoclast differentiation | Signal transduction-PI3K-Akt pathway | 167 | 0.0259 |
| Osteoclast differentiation | visual perception | 3 | 0.0018 |
| Osteoclast differentiation | Camera-type eye development | 25 | 0.0247 |
| Osteoclast differentiation | Prepulse inhibition | 237 | 0.0348 |
| Osteoclast differentiation | Regulation of signal transduction | 90 | 0.0268 |
| Osteoclast differentiation | miR-1909-3p-PRR35 | 51 | 0.0246 |
| Osteoclast differentiation | miR-4795-5p-LOC100506469 | 110 | 0.0854 |
| Osteoclast differentiation | Inflammatory response-Salmonella infection | 426 | 0.1625 |
| Osteoclast differentiation | MIR568-SPEM1 | 15 | 0.0217 |
| Osteoclast differentiation | Response to calcium ion-Complement and coagulation cascades | 24 | 0.0180 |
| Osteoclast differentiation | miR-4766-5p-ADCY10P1 | 36 | 0.0326 |
| Osteoclast differentiation | Mesenchyme migration | 310 | 0.0963 |
| Osteoclast differentiation | Negative regulation of epithelial cell proliferation | 30 | 0.0311 |
| Osteoclast differentiation | miR-206-OR2F1 | 29 | 0.0191 |
| Osteoclast differentiation | miR-4670-3p-ZNF702P | 36 | 0.0559 |
| G-protein coupled receptor signaling pathway-salivary secretion | Signal transduction-PI3K-Akt pathway | 318 | 0.0505 |
| G-protein coupled receptor signaling pathway-salivary secretion | visual perception | 42 | 0.0252 |
| G-protein coupled receptor signaling pathway-salivary secretion | Camera-type eye development | 67 | 0.0677 |
| G-protein coupled receptor signaling pathway-salivary secretion | Prepulse inhibition | 518 | 0.0778 |
| G-protein coupled receptor signaling pathway-salivary secretion | Regulation of signal transduction | 125 | 0.0381 |
| G-protein coupled receptor signaling pathway-salivary secretion | miR-1909-3p-PRR35 | 137 | 0.0677 |
| G-protein coupled receptor signaling pathway-salivary secretion | miR-4795-5p-LOC100506469 | 64 | 0.0508 |
| G-protein coupled receptor signaling pathway-salivary secretion | Inflammatory response-Salmonella infection | 43 | 0.0168 |
| G-protein coupled receptor signaling pathway-salivary secretion | Response to calcium ion-Complement and coagulation cascades | 29 | 0.0222 |
| G-protein coupled receptor signaling pathway-salivary secretion | miR-4766-5p-ADCY10P1 | 1 | 0.0009 |
| G-protein coupled receptor signaling pathway-salivary secretion | Mesenchyme migration | 144 | 0.0457 |
| G-protein coupled receptor signaling pathway-salivary secretion | Negative regulation of epithelial cell proliferation | 3 | 0.0032 |
| G-protein coupled receptor signaling pathway-salivary secretion | miR-206-OR2F1 | 37 | 0.0249 |
| G-protein coupled receptor signaling pathway-salivary secretion | miR-4670-3p-ZNF702P | 24 | 0.0381 |
| Signal transduction-PI3K-Akt pathway | visual perception | 162 | 0.0313 |
| Signal transduction-PI3K-Akt pathway | Camera-type eye development | 178 | 0.0578 |
| Signal transduction-PI3K-Akt pathway | Prepulse inhibition | 1305 | 0.0630 |
| Signal transduction-PI3K-Akt pathway | Regulation of signal transduction | 471 | 0.0461 |
| Signal transduction-PI3K-Akt pathway | miR-1909-3p-PRR35 | 260 | 0.0413 |
| Signal transduction-PI3K-Akt pathway | miR-4795-5p-LOC100506469 | 117 | 0.0298 |
| Signal transduction-PI3K-Akt pathway | Inflammatory response-Salmonella infection | 569 | 0.0713 |
| Signal transduction-PI3K-Akt pathway | MIR568-SPEM1 | 21 | 0.0100 |
| Signal transduction-PI3K-Akt pathway | Response to calcium ion-Complement and coagulation cascades | 116 | 0.0286 |
| Signal transduction-PI3K-Akt pathway | miR-4766-5p-ADCY10P1 | 40 | 0.0119 |
| Signal transduction-PI3K-Akt pathway | Mesenchyme migration | 719 | 0.0734 |
| Signal transduction-PI3K-Akt pathway | Negative regulation of epithelial cell proliferation | 438 | 0.1490 |
| Signal transduction-PI3K-Akt pathway | miR-206-OR2F1 | 217 | 0.0470 |
| Signal transduction-PI3K-Akt pathway | miR-4670-3p-ZNF702P | 129 | 0.0658 |
| visual perception | Camera-type eye development | 16 | 0.0197 |
| visual perception | Prepulse inhibition | 348 | 0.0636 |
| visual perception | Regulation of signal transduction | 38 | 0.0141 |
| visual perception | miR-1909-3p-PRR35 | 54 | 0.0324 |
| visual perception | miR-4795-5p-LOC100506469 | 100 | 0.0965 |
| visual perception | Inflammatory response-Salmonella infection | 57 | 0.0270 |
| visual perception | Response to calcium ion-Complement and coagulation cascades | 100 | 0.0932 |
| visual perception | miR-4766-5p-ADCY10P1 | 38 | 0.0428 |
| visual perception | Mesenchyme migration | 69 | 0.0266 |
| visual perception | Negative regulation of epithelial cell proliferation | 58 | 0.0746 |
| visual perception | miR-206-OR2F1 | 2 | 0.0016 |
| Camera-type eye development | Prepulse inhibition | 152 | 0.0467 |
| Camera-type eye development | Regulation of signal transduction | 5 | 0.0031 |
| Camera-type eye development | miR-1909-3p-PRR35 | 11 | 0.0111 |
| Camera-type eye development | miR-4795-5p-LOC100506469 | 84 | 0.1364 |
| Camera-type eye development | Inflammatory response-Salmonella infection | 48 | 0.0383 |
| Camera-type eye development | Response to calcium ion-Complement and coagulation cascades | 58 | 0.0909 |
| Camera-type eye development | miR-4766-5p-ADCY10P1 | 16 | 0.0303 |
| Camera-type eye development | Mesenchyme migration | 139 | 0.0903 |
| Camera-type eye development | miR-206-OR2F1 | 40 | 0.0551 |
| Camera-type eye development | miR-4670-3p-ZNF702P | 1 | 0.0032 |
| Prepulse inhibition | Regulation of signal transduction | 8322 | 0.7703 |
| Prepulse inhibition | miR-1909-3p-PRR35 | 612 | 0.0919 |
| Prepulse inhibition | miR-4795-5p-LOC100506469 | 105 | 0.0253 |
| Prepulse inhibition | Inflammatory response-Salmonella infection | 270 | 0.0320 |
| Prepulse inhibition | MIR568-SPEM1 | 165 | 0.0743 |
| Prepulse inhibition | Response to calcium ion-Complement and coagulation cascades | 267 | 0.0622 |
| Prepulse inhibition | miR-4766-5p-ADCY10P1 | 131 | 0.0369 |
| Prepulse inhibition | Mesenchyme migration | 383 | 0.0370 |
| Prepulse inhibition | Negative regulation of epithelial cell proliferation | 270 | 0.0869 |
| Prepulse inhibition | miR-206-OR2F1 | 145 | 0.0297 |
| Prepulse inhibition | miR-4670-3p-ZNF702P | 169 | 0.0816 |
| Regulation of signal transduction | miR-1909-3p-PRR35 | 119 | 0.0362 |
| Regulation of signal transduction | miR-4795-5p-LOC100506469 | 51 | 0.0250 |
| Regulation of signal transduction | Inflammatory response-Salmonella infection | 97 | 0.0233 |
| Regulation of signal transduction | MIR568-SPEM1 | 33 | 0.0301 |
| Regulation of signal transduction | Response to calcium ion-Complement and coagulation cascades | 114 | 0.0538 |
| Regulation of signal transduction | miR-4766-5p-ADCY10P1 | 62 | 0.0354 |
| Regulation of signal transduction | Mesenchyme migration | 297 | 0.0581 |
| Regulation of signal transduction | Negative regulation of epithelial cell proliferation | 97 | 0.0633 |
| Regulation of signal transduction | miR-206-OR2F1 | 17 | 0.0071 |
| Regulation of signal transduction | miR-4670-3p-ZNF702P | 9 | 0.0088 |
| miR-1909-3p-PRR35 | miR-4795-5p-LOC100506469 | 62 | 0.0492 |
| miR-1909-3p-PRR35 | Inflammatory response-Salmonella infection | 288 | 0.1123 |
| miR-1909-3p-PRR35 | MIR568-SPEM1 | 15 | 0.0222 |
| miR-1909-3p-PRR35 | Response to calcium ion-Complement and coagulation cascades | 32 | 0.0245 |
| miR-1909-3p-PRR35 | miR-4766-5p-ADCY10P1 | 26 | 0.0241 |
| miR-1909-3p-PRR35 | Mesenchyme migration | 4 | 0.0013 |
| miR-1909-3p-PRR35 | Negative regulation of epithelial cell proliferation | 14 | 0.0148 |
| miR-1909-3p-PRR35 | miR-206-OR2F1 | 110 | 0.0741 |
| miR-4795-5p-LOC100506469 | Inflammatory response-Salmonella infection | 158 | 0.0990 |
| miR-4795-5p-LOC100506469 | MIR568-SPEM1 | 12 | 0.0286 |
| miR-4795-5p-LOC100506469 | miR-4766-5p-ADCY10P1 | 32 | 0.0476 |
| miR-4795-5p-LOC100506469 | Mesenchyme migration | 172 | 0.0878 |
| miR-4795-5p-LOC100506469 | miR-206-OR2F1 | 4 | 0.0043 |
| Inflammatory response-Salmonella infection | MIR568-SPEM1 | 59 | 0.0690 |
| Inflammatory response-Salmonella infection | Response to calcium ion-Complement and coagulation cascades | 27 | 0.0163 |
| Inflammatory response-Salmonella infection | miR-4766-5p-ADCY10P1 | 139 | 0.1016 |
| Inflammatory response-Salmonella infection | Mesenchyme migration | 63 | 0.0158 |
| Inflammatory response-Salmonella infection | Negative regulation of epithelial cell proliferation | 51 | 0.0426 |
| Inflammatory response-Salmonella infection | miR-206-OR2F1 | 103 | 0.0548 |
| Inflammatory response-Salmonella infection | miR-4670-3p-ZNF702P | 14 | 0.0175 |
| MIR568-SPEM1 | miR-4766-5p-ADCY10P1 | 1 | 0.0028 |
| MIR568-SPEM1 | Mesenchyme migration | 65 | 0.0619 |
| Response to calcium ion-Complement and coagulation cascades | miR-4766-5p-ADCY10P1 | 46 | 0.0661 |
| Response to calcium ion-Complement and coagulation cascades | Mesenchyme migration | 74 | 0.0365 |
| Response to calcium ion-Complement and coagulation cascades | Negative regulation of epithelial cell proliferation | 163 | 0.2677 |
| Response to calcium ion-Complement and coagulation cascades | miR-206-OR2F1 | 22 | 0.0230 |
| miR-4766-5p-ADCY10P1 | Mesenchyme migration | 156 | 0.0929 |
| miR-4766-5p-ADCY10P1 | Negative regulation of epithelial cell proliferation | 25 | 0.0496 |
| miR-4766-5p-ADCY10P1 | miR-206-OR2F1 | 27 | 0.0341 |
| Mesenchyme migration | Negative regulation of epithelial cell proliferation | 154 | 0.1048 |
| Mesenchyme migration | miR-206-OR2F1 | 203 | 0.0879 |
| Negative regulation of epithelial cell proliferation | miR-206-OR2F1 | 13 | 0.0188 |

**Table S16. The enriched BP-GOs and KEGG pathways of the targeted modules of DHI**

| **ID** | **Module color** | **Category** | **Term** | **Gene-Count** | **Ratio** | **Genes** | **P-Value** |
| --- | --- | --- | --- | --- | --- | --- | --- |
| 1 | cyan | GOTERM_BP_DIRECT | GO:0050907~detection of chemical stimulus involved in sensory perception | 3 | 7.3171 | OR4F16, OR4F29, OR4F3 | 0.0056 |
| 2 | darkgreen | GOTERM_BP_DIRECT | GO:0009566~fertilization | 2 | 4.8780 | SPINK2, SPATA22 | 0.0443 |
| 3 | darkolivegreen | GOTERM_BP_DIRECT | GO:0019882~antigen processing and presentation | 2 | 10.0000 | HLA-H, RAET1L | 0.0291 |
| 4 | darkorange | GOTERM_BP_DIRECT | GO:0007156~homophilic cell adhesion via plasma membrane adhesion molecules | 3 | 14.2857 | PCDHB5, PCDHB2, PCDH19 | 0.0055 |
| 5 | darkorange | GOTERM_BP_DIRECT | GO:0016339~calcium-dependent cell-cell adhesion via plasma membrane cell adhesion molecules | 2 | 9.5238 | PCDHB5, PCDHB2 | 0.0198 |
| 6 | darkorange | GOTERM_BP_DIRECT | GO:0007416~synapse assembly | 2 | 9.5238 | PCDHB5, PCDHB2 | 0.0427 |
| 7 | grey60 | GOTERM_BP_DIRECT | GO:0045087~innate immune response | 5 | 11.3636 | LCN2, CRISP3, CAMP, DEFA3, DMBT1 | 0.0061 |
| 8 | grey60 | GOTERM_BP_DIRECT | GO:0002227~innate immune response in mucosa | 3 | 6.8182 | CAMP, DEFA3, LTF | 0.0008 |
| 9 | grey60 | GOTERM_BP_DIRECT | GO:0019731~antibacterial humoral response | 3 | 6.8182 | CAMP, DEFA3, LTF | 0.0026 |
| 10 | grey60 | GOTERM_BP_DIRECT | GO:0050900~leukocyte migration | 3 | 6.8182 | OLR1, CEACAM8, CEACAM6 | 0.0187 |
| 11 | grey60 | GOTERM_BP_DIRECT | GO:0042742~defense response to bacterium | 3 | 6.8182 | CAMP, DEFA3, HP | 0.0258 |
| 12 | grey60 | GOTERM_BP_DIRECT | GO:0006898~receptor-mediated endocytosis | 3 | 6.8182 | OLR1, HP, DMBT1 | 0.0407 |
| 13 | grey60 | GOTERM_BP_DIRECT | GO:0019732~antifungal humoral response | 2 | 4.5455 | CAMP, LTF | 0.0171 |
| 14 | lightcyan | GOTERM_BP_DIRECT | GO:0008277~regulation of G-protein coupled receptor protein signaling pathway | 2 | 7.6923 | GPR158, RGS4 | 0.0343 |
| 15 | lightyellow | GOTERM_BP_DIRECT | GO:0007186~G-protein coupled receptor signaling pathway | 5 | 14.7059 | RGS7BP, CGB2, OR1L4, TAAR1, ADRA1D | 0.0111 |
| 16 | magenta | GOTERM_BP_DIRECT | GO:0007165~signal transduction | 13 | 10.1563 | EGFR, CAP2, GULP1, GNG12, KCNIP3, LINGO1, SYDE1, PDE1C, SMOC1, PDE1A, ANTXR1, NR2F2, GRB7 | 0.0305 |
| 17 | magenta | GOTERM_BP_DIRECT | GO:0007155~cell adhesion | 9 | 7.0313 | RND3, FAT1, COL12A1, BCAN, CHST4, COL1A1, CX3CL1, CYR61, THY1 | 0.0045 |
| 18 | magenta | GOTERM_BP_DIRECT | GO:0001501~skeletal system development | 8 | 6.2500 | PTHLH, ARSE, COL3A1, COL12A1, BCAN, COL1A1, COL5A2, TLL1 | 0.0000 |
| 19 | magenta | GOTERM_BP_DIRECT | GO:0007267~cell-cell signaling | 8 | 6.2500 | PTHLH, SSTR1, GHRH, FAT1, EFNA2, CHST4, SHH, CYR61 | 0.0006 |
| 20 | magenta | GOTERM_BP_DIRECT | GO:0043066~negative regulation of apoptotic process | 8 | 6.2500 | EGFR, DHRS2, FMN2, SFRP1, RGN, WT1, SHH, CYR61 | 0.0147 |
| 21 | magenta | GOTERM_BP_DIRECT | GO:0030198~extracellular matrix organization | 7 | 5.4688 | COL4A2, BGN, COL3A1, BCAN, COL1A1, COL5A2, CYR61 | 0.0009 |
| 22 | magenta | GOTERM_BP_DIRECT | GO:0008284~positive regulation of cell proliferation | 7 | 5.4688 | PTHLH, TRPM4, EGFR, SFRP1, GHRH, LIFR, SHH | 0.0487 |
| 23 | magenta | GOTERM_BP_DIRECT | GO:0048706~embryonic skeletal system development | 5 | 3.9063 | HOXC6, DLX1, SULF1, COL1A1, SHH | 0.0000 |
| 24 | magenta | GOTERM_BP_DIRECT | GO:0030574~collagen catabolic process | 5 | 3.9063 | COL4A2, COL3A1, COL12A1, COL1A1, COL5A2 | 0.0005 |
| 25 | magenta | GOTERM_BP_DIRECT | GO:0030336~negative regulation of cell migration | 5 | 3.9063 | SFRP1, SULF1, CX3CL1, SHH, THY1 | 0.0021 |
| 26 | magenta | GOTERM_BP_DIRECT | GO:0007411~axon guidance | 5 | 3.9063 | EPHA5, ARX, SEMA3F, EFNA2, SHH | 0.0127 |
| 27 | magenta | GOTERM_BP_DIRECT | GO:0030335~positive regulation of cell migration | 5 | 3.9063 | EGFR, SEMA3F, COL1A1, GRB7, CYR61 | 0.0205 |
| 28 | magenta | GOTERM_BP_DIRECT | GO:0030199~collagen fibril organization | 4 | 3.1250 | COL3A1, COL12A1, COL1A1, COL5A2 | 0.0014 |
| 29 | magenta | GOTERM_BP_DIRECT | GO:0030900~forebrain development | 4 | 3.1250 | DKK1, SSTR1, NR2F2, SHH | 0.0022 |
| 30 | magenta | GOTERM_BP_DIRECT | GO:0071230~cellular response to amino acid stimulus | 4 | 3.1250 | EGFR, COL3A1, COL1A1, COL5A2 | 0.0024 |
| 31 | magenta | GOTERM_BP_DIRECT | GO:0071456~cellular response to hypoxia | 4 | 3.1250 | FMN2, STC2, SFRP1, MGARP | 0.0172 |
| 32 | magenta | GOTERM_BP_DIRECT | GO:0016337~single organismal cell-cell adhesion | 4 | 3.1250 | EGFR, KIRREL, FAT1, THY1 | 0.0196 |
| 33 | magenta | GOTERM_BP_DIRECT | GO:0071392~cellular response to estradiol stimulus | 3 | 2.3438 | EGFR, SFRP1, SSTR1 | 0.0132 |
| 34 | magenta | GOTERM_BP_DIRECT | GO:0030177~positive regulation of Wnt signaling pathway | 3 | 2.3438 | SFRP1, SULF1, SHH | 0.0176 |
| 35 | magenta | GOTERM_BP_DIRECT | GO:0043010~camera-type eye development | 3 | 2.3438 | EFEMP1, WT1, SHH | 0.0215 |
| 36 | magenta | GOTERM_BP_DIRECT | GO:0061337~cardiac conduction | 3 | 2.3438 | TRPM4, KCNK15, KCNIP3 | 0.0268 |
| 37 | magenta | GOTERM_BP_DIRECT | GO:0071560~cellular response to transforming growth factor beta stimulus | 3 | 2.3438 | COL4A2, SFRP1, COL1A1 | 0.0314 |
| 38 | magenta | GOTERM_BP_DIRECT | GO:0007173~epidermal growth factor receptor signaling pathway | 3 | 2.3438 | EGFR, EFEMP1, GRB7 | 0.0400 |
| 39 | magenta | GOTERM_BP_DIRECT | GO:0021766~hippocampus development | 3 | 2.3438 | EPHA5, DLX1, BCAN | 0.0400 |
| 40 | magenta | GOTERM_BP_DIRECT | GO:0017148~negative regulation of translation | 3 | 2.3438 | IGF2BP1, GRB7, WT1 | 0.0427 |
| 41 | magenta | GOTERM_BP_DIRECT | GO:0006477~protein sulfation | 2 | 1.5625 | HS3ST5, CHST4 | 0.0280 |
| 42 | magenta | GOTERM_BP_DIRECT | GO:0019896~axonal transport of mitochondrion | 2 | 1.5625 | UCHL1, MGARP | 0.0335 |
| 43 | magenta | GOTERM_BP_DIRECT | GO:0060074~synapse maturation | 2 | 1.5625 | SEZ6L2, PALM | 0.0389 |
| 44 | magenta | GOTERM_BP_DIRECT | GO:0060346~bone trabecula formation | 2 | 1.5625 | SFRP1, COL1A1 | 0.0444 |
| 45 | magenta | GOTERM_BP_DIRECT | GO:0090244~Wnt signaling pathway involved in somitogenesis | 2 | 1.5625 | DKK1, SFRP1 | 0.0498 |
| 46 | magenta | GOTERM_BP_DIRECT | GO:0032836~glomerular basement membrane development | 2 | 1.5625 | SULF1, WT1 | 0.0498 |
| 47 | orange | GOTERM_BP_DIRECT | GO:0007601~visual perception | 3 | 11.5385 | CRYGD, OPN1LW, CRYBB3 | 0.0213 |
| 48 | orange | GOTERM_BP_DIRECT | GO:0060292~long term synaptic depression | 2 | 7.6923 | GRIA1, SHANK2 | 0.0180 |
| 49 | paleturquoise | GOTERM_BP_DIRECT | GO:0043010~camera-type eye development | 2 | 12.5000 | PAX2, MAB21L1 | 0.0189 |
| 50 | paleturquoise | GOTERM_BP_DIRECT | GO:0009653~anatomical structure morphogenesis | 2 | 12.5000 | EN1, MAB21L1 | 0.0430 |
| 51 | paleturquoise | GOTERM_BP_DIRECT | GO:0050907~detection of chemical stimulus involved in sensory perception | 2 | 12.5000 | OR4X2, OR4A47 | 0.0444 |
| 52 | pink | GOTERM_BP_DIRECT | GO:0060134~prepulse inhibition | 2 | 2.3810 | GRIN1, CTNNA2 | 0.0268 |
| 53 | purple | GOTERM_BP_DIRECT | GO:0009966~regulation of signal transduction | 2 | 6.6667 | PPP1R2P9, CNKSR3 | 0.0247 |
| 54 | salmon | GOTERM_BP_DIRECT | GO:0006954~inflammatory response | 6 | 15.7895 | CCL20, CCL3L1, CXCL3, CXCL2, IL1B, IL1A | 0.0001 |
| 55 | salmon | GOTERM_BP_DIRECT | GO:0006955~immune response | 5 | 13.1579 | CCL20, CXCL3, CXCL2, IL1B, IL1A | 0.0020 |
| 56 | salmon | GOTERM_BP_DIRECT | GO:0007186~G-protein coupled receptor signaling pathway | 5 | 13.1579 | CCL20, CCL3L1, CXCL3, CXCL2, AREG | 0.0276 |
| 57 | salmon | GOTERM_BP_DIRECT | GO:0030593~neutrophil chemotaxis | 4 | 10.5263 | CCL20, CCL3L1, CXCL3, IL1B | 0.0001 |
| 58 | salmon | GOTERM_BP_DIRECT | GO:0070098~chemokine-mediated signaling pathway | 4 | 10.5263 | CCL20, CCL3L1, CXCL3, CXCL2 | 0.0001 |
| 59 | salmon | GOTERM_BP_DIRECT | GO:0019221~cytokine-mediated signaling pathway | 3 | 7.8947 | IL20RA, IL1B, IL1A | 0.0126 |
| 60 | salmon | GOTERM_BP_DIRECT | GO:0007267~cell-cell signaling | 3 | 7.8947 | CCL20, IL1B, AREG | 0.0432 |
| 61 | salmon | GOTERM_BP_DIRECT | GO:0001660~fever generation | 2 | 5.2632 | IL1B, IL1A | 0.0039 |
| 62 | salmon | GOTERM_BP_DIRECT | GO:0071639~positive regulation of monocyte chemotactic protein-1 production | 2 | 5.2632 | IL1B, IL1A | 0.0091 |
| 63 | salmon | GOTERM_BP_DIRECT | GO:0035234~ectopic germ cell programmed cell death | 2 | 5.2632 | IL1B, IL1A | 0.0104 |
| 64 | salmon | GOTERM_BP_DIRECT | GO:0045086~positive regulation of interleukin-2 biosynthetic process | 2 | 5.2632 | IL1B, IL1A | 0.0156 |
| 65 | salmon | GOTERM_BP_DIRECT | GO:0090023~positive regulation of neutrophil chemotaxis | 2 | 5.2632 | CXCL3, CXCL2 | 0.0284 |
| 66 | salmon | GOTERM_BP_DIRECT | GO:0045840~positive regulation of mitotic nuclear division | 2 | 5.2632 | IL1B, IL1A | 0.0335 |
| 67 | salmon | GOTERM_BP_DIRECT | GO:0010575~positive regulation of vascular endothelial growth factor production | 2 | 5.2632 | IL1B, IL1A | 0.0348 |
| 68 | salmon | GOTERM_BP_DIRECT | GO:0048247~lymphocyte chemotaxis | 2 | 5.2632 | CCL20, CCL3L1 | 0.0361 |
| 69 | salmon | GOTERM_BP_DIRECT | GO:0097192~extrinsic apoptotic signaling pathway in absence of ligand | 2 | 5.2632 | IL1B, IL1A | 0.0436 |
| 70 | salmon | GOTERM_BP_DIRECT | GO:2001240~negative regulation of extrinsic apoptotic signaling pathway in absence of ligand | 2 | 5.2632 | IL1B, IL1A | 0.0474 |
| 71 | skyblue | GOTERM_BP_DIRECT | GO:0051592~response to calcium ion | 3 | 10.7143 | FGG, FGA, MTTP | 0.0024 |
| 72 | skyblue | GOTERM_BP_DIRECT | GO:0002576~platelet degranulation | 3 | 10.7143 | TF, FGG, FGA | 0.0073 |
| 73 | skyblue | GOTERM_BP_DIRECT | GO:0007596~blood coagulation | 3 | 10.7143 | FGG, FGA, SERPINA5 | 0.0219 |
| 74 | skyblue | GOTERM_BP_DIRECT | GO:0072378~blood coagulation, fibrin clot formation | 2 | 7.1429 | FGG, FGA | 0.0050 |
| 75 | skyblue | GOTERM_BP_DIRECT | GO:0090277~positive regulation of peptide hormone secretion | 2 | 7.1429 | FGG, FGA | 0.0100 |
| 76 | skyblue | GOTERM_BP_DIRECT | GO:0031639~plasminogen activation | 2 | 7.1429 | FGG, FGA | 0.0112 |
| 77 | skyblue | GOTERM_BP_DIRECT | GO:0034116~positive regulation of heterotypic cell-cell adhesion | 2 | 7.1429 | FGG, FGA | 0.0137 |
| 78 | skyblue | GOTERM_BP_DIRECT | GO:0051258~protein polymerization | 2 | 7.1429 | FGG, FGA | 0.0161 |
| 79 | skyblue | GOTERM_BP_DIRECT | GO:0043623~cellular protein complex assembly | 2 | 7.1429 | FGG, FGA | 0.0223 |
| 80 | skyblue | GOTERM_BP_DIRECT | GO:0042730~fibrinolysis | 2 | 7.1429 | FGG, FGA | 0.0260 |
| 81 | skyblue | GOTERM_BP_DIRECT | GO:0045921~positive regulation of exocytosis | 2 | 7.1429 | FGG, FGA | 0.0260 |
| 82 | skyblue | GOTERM_BP_DIRECT | GO:2000352~negative regulation of endothelial cell apoptotic process | 2 | 7.1429 | FGG, FGA | 0.0345 |
| 83 | skyblue | GOTERM_BP_DIRECT | GO:1900026~positive regulation of substrate adhesion-dependent cell spreading | 2 | 7.1429 | FGG, FGA | 0.0393 |
| 84 | skyblue | GOTERM_BP_DIRECT | GO:0045907~positive regulation of vasoconstriction | 2 | 7.1429 | FGG, FGA | 0.0393 |
| 85 | skyblue | GOTERM_BP_DIRECT | GO:1902042~negative regulation of extrinsic apoptotic signaling pathway via death domain receptors | 2 | 7.1429 | FGG, FGA | 0.0405 |
| 86 | skyblue | GOTERM_BP_DIRECT | GO:0050714~positive regulation of protein secretion | 2 | 7.1429 | FGG, FGA | 0.0441 |
| 87 | tan | GOTERM_BP_DIRECT | GO:0090131~mesenchyme migration | 2 | 3.5088 | ACTC1, FOXF1 | 0.0116 |
| 88 | tan | GOTERM_BP_DIRECT | GO:0097105~presynaptic membrane assembly | 2 | 3.5088 | PTPRD, LRP4 | 0.0207 |
| 89 | tan | GOTERM_BP_DIRECT | GO:0055008~cardiac muscle tissue morphogenesis | 2 | 3.5088 | ACTC1, ANKRD1 | 0.0275 |
| 90 | tan | GOTERM_BP_DIRECT | GO:0030048~actin filament-based movement | 2 | 3.5088 | ACTC1, MYH14 | 0.0388 |
| 91 | violent | GOTERM_BP_DIRECT | GO:0050680~negative regulation of epithelial cell proliferation | 2 | 9.5238 | GPC3, NKX3-1 | 0.0457 |
| 92 | violent | GOTERM_BP_DIRECT | GO:0001523~retinoid metabolic process | 2 | 9.5238 | TTR, GPC3 | 0.0497 |
| 93 | darkgreen | KEGG_PATHWAY | hsa01100:Metabolic pathways | 5 | 12.1951 | GCK, ASMT, PRPS1L1, UROC1, CYP4F2 | 0.0380 |
| 94 | lightgreen | KEGG_PATHWAY | hsa04380:Osteoclast differentiation | 3 | 8.1081 | TNF, LILRB5, LILRA3 | 0.0070 |
| 95 | lightyellow | KEGG_PATHWAY | hsa04970:Salivary secretion | 2 | 5.8824 | CST5, ADRA1D | 0.0369 |
| 96 | magenta | KEGG_PATHWAY | hsa04151:PI3K-Akt signaling pathway | 8 | 6.2500 | EGFR, COL4A2, EFNA2, COL3A1, GNG13, COL1A1, GNG12, COL5A2 | 0.0028 |
| 97 | magenta | KEGG_PATHWAY | hsa04974:Protein digestion and absorption | 6 | 4.6875 | COL4A2, COL3A1, COL12A1, PGA3, COL1A1, COL5A2 | 0.0001 |
| 98 | magenta | KEGG_PATHWAY | hsa04510:Focal adhesion | 6 | 4.6875 | EGFR, COL4A2, COL3A1, COL1A1, FLNC, COL5A2 | 0.0057 |
| 99 | magenta | KEGG_PATHWAY | hsa04512:ECM-receptor interaction | 4 | 3.1250 | COL4A2, COL3A1, COL1A1, COL5A2 | 0.0127 |
| 100 | magenta | KEGG_PATHWAY | hsa05032:Morphine addiction | 4 | 3.1250 | PDE1C, PDE1A, GNG13, GNG12 | 0.0143 |
| 101 | magenta | KEGG_PATHWAY | hsa05146:Amoebiasis | 4 | 3.1250 | COL4A2, COL3A1, COL1A1, COL5A2 | 0.0215 |
| 102 | salmon | KEGG_PATHWAY | hsa05132:Salmonella infection | 5 | 13.1579 | CCL3L1, CXCL3, CXCL2, IL1B, IL1A | 0.0000 |
| 103 | salmon | KEGG_PATHWAY | hsa05323:Rheumatoid arthritis | 5 | 13.1579 | CCL20, CCL3L1, IL1B, ATP6V1G3, IL1A | 0.0000 |
| 104 | salmon | KEGG_PATHWAY | hsa04060:Cytokine-cytokine receptor interaction | 5 | 13.1579 | CCL20, IL20RA, CCL3L1, IL1B, IL1A | 0.0012 |
| 105 | salmon | KEGG_PATHWAY | hsa04668:TNF signaling pathway | 4 | 10.5263 | CCL20, CXCL3, CXCL2, IL1B | 0.0014 |
| 106 | salmon | KEGG_PATHWAY | hsa04062:Chemokine signaling pathway | 4 | 10.5263 | CCL20, CCL3L1, CXCL3, CXCL2 | 0.0069 |
| 107 | salmon | KEGG_PATHWAY | hsa05134:Legionellosis | 3 | 7.8947 | CXCL3, CXCL2, IL1B | 0.0059 |
| 108 | skyblue | KEGG_PATHWAY | hsa04610:Complement and coagulation cascades | 3 | 10.7143 | FGG, FGA, SERPINA5 | 0.0061 |

**Table S17. The corralation between the 90 DEGs at Day 30 and theΔAF**

| DEG name | Cor | p-value |
| --- | --- | --- |
| TPTE | -0.457 | 0.004 |
| ACOXL | -0.279 | 0.095 |
| ADAMTS7 | -0.238 | 0.156 |
| DIO2 | -0.187 | 0.268 |
| SNORD19B | -0.171 | 0.311 |
| CCER2 | -0.165 | 0.329 |
| APLP1 | -0.159 | 0.347 |
| GIPC2 | -0.143 | 0.398 |
| PDZRN3 | -0.142 | 0.401 |
| C1orf95 | -0.127 | 0.455 |
| RPPH1 | -0.116 | 0.495 |
| hsa.miR.3941 | -0.110 | 0.518 |
| ERAS | -0.098 | 0.564 |
| LOC286190 | -0.095 | 0.577 |
| OPN1MW | -0.094 | 0.580 |
| TBC1D29 | -0.093 | 0.585 |
| RNVU1.18 | -0.092 | 0.586 |
| RNU1.27P | -0.092 | 0.586 |
| RNU1.28P | -0.092 | 0.586 |
| RNU1.3 | -0.092 | 0.586 |
| RNU1.2 | -0.092 | 0.586 |
| RNU1.1 | -0.092 | 0.586 |
| RNU1.4 | -0.092 | 0.586 |
| NXF5 | -0.092 | 0.590 |
| hsa.miR.548ad.5p | -0.091 | 0.592 |
| GCOM1 | -0.091 | 0.593 |
| MAG | -0.089 | 0.601 |
| hsa.miR.6848.5p | -0.088 | 0.605 |
| RPL13AP20 | -0.079 | 0.642 |
| C19orf26 | -0.074 | 0.661 |
| MEGF11 | -0.072 | 0.671 |
| SLIT3 | -0.071 | 0.676 |
| CCDC80 | -0.064 | 0.706 |
| PRSS1 | -0.059 | 0.728 |
| IL36A | -0.059 | 0.731 |
| IDH1.AS1 | -0.058 | 0.733 |
| LOC649330 | -0.054 | 0.753 |
| LURAP1L | -0.045 | 0.792 |
| SCARA3 | -0.042 | 0.803 |
| RTN4RL2 | -0.039 | 0.817 |
| hsa.miR.3200.5p | -0.039 | 0.819 |
| MIR5194 | -0.031 | 0.854 |
| hsa.miR.1246 | -0.030 | 0.858 |
| PTTG2 | -0.030 | 0.861 |
| RNU5D.1 | -0.019 | 0.910 |
| GDF6 | -0.017 | 0.922 |
| OR1J1 | -0.014 | 0.935 |
| ADORA3 | -0.002 | 0.989 |
| HIST1H1B | -0.001 | 0.996 |
| SHC4 | 0.003 | 0.988 |
| hsa.miR.4461 | 0.009 | 0.957 |
| C12orf40 | 0.012 | 0.945 |
| SNORD35A | 0.029 | 0.866 |
| MIR4658 | 0.033 | 0.847 |
| LINC00515 | 0.034 | 0.843 |
| MIR4481 | 0.050 | 0.770 |
| SNORD36C | 0.053 | 0.758 |
| MIR4712 | 0.074 | 0.663 |
| SELV | 0.076 | 0.655 |
| novel_mir_612 | 0.077 | 0.651 |
| GPRC5A | 0.081 | 0.635 |
| IL20RA | 0.087 | 0.609 |
| hsa.miR.374c.5p | 0.087 | 0.608 |
| DPY19L2P2 | 0.091 | 0.591 |
| HIST1H2BM | 0.106 | 0.531 |
| TOX3 | 0.107 | 0.529 |
| ADAMTS14 | 0.112 | 0.509 |
| C6orf223 | 0.118 | 0.487 |
| SNORA21 | 0.122 | 0.472 |
| ACADL | 0.135 | 0.425 |
| ARHGEF15 | 0.139 | 0.410 |
| hsa.miR.3180.5p | 0.141 | 0.405 |
| novel_mir_95 | 0.142 | 0.403 |
| MIR630 | 0.175 | 0.301 |
| NKX2.1 | 0.196 | 0.246 |
| ZNF541 | 0.199 | 0.237 |
| EDA2R | 0.200 | 0.236 |
| CCDC42 | 0.221 | 0.189 |
| hsa.miR.7151.3p | 0.246 | 0.142 |
| AQP5 | 0.254 | 0.130 |
| SNORA76A | 0.262 | 0.117 |
| TMEM108 | 0.276 | 0.099 |
| novel_mir_97 | 0.292 | 0.079 |
| PCDH17 | 0.342 | 0.039 |
| hsa.miR.1273f | NA | NA |
| hsa.miR.3136.3p | NA | NA |
| HGC6.3 | NA | NA |
| CEACAM7 | NA | NA |
| CT55 | NA | NA |
| TCP10 | NA | NA |

**Table. S18. The enrichment on the GO biological process for the effective therapeutic module**

| **GOTERM_BP_DIRECT** | **P-value** | **Genes** | **Fold Enrichment** | **Bonferroni** | **Benjamini** | **FDR** |
| --- | --- | --- | --- | --- | --- | --- |
| GO:0051923~sulfation | 0.0169 | SULT1A2, SULT1E1 | 113.0774 | 0.9601 | 0.9601 | 19.0813 |
| GO:0030195~negative regulation of blood coagulation | 0.0184 | SERPINE2, VTN | 103.6543 | 0.9703 | 0.8276 | 20.6245 |
| GO:0050427~3'-phosphoadenosine 5'-phosphosulfate metabolic process | 0.0230 | SULT1A2, SULT1E1 | 82.923457 | 0.9877 | 0.7689 | 25.0802 |
